# Towards the sequence-specific multivalent molecular recognition of cyclodextrin oligomers

**DOI:** 10.3762/bjoc.10.253

**Published:** 2014-10-20

**Authors:** Michael Kurlemann, Bart Jan Ravoo

**Affiliations:** 1 Organic Chemistry Institute, Westfälische Wilhelms-Universität Münster, Corrensstrasse 40, 48149 Münster, Germany

**Keywords:** cooperativity, cyclodextrins, molecular recognition, multivalency, sequence specificity

## Abstract

Sequence-specific multivalent molecular recognition has been recognized to play a major role in biological processes. Furthermore, sequence-specific recognition motifs have been used in various artificial systems in the last years, e.g., to emulate biological processes or to build up new materials with highly specific recognition domains. In this article, we present the preparation of cyclodextrin (CD)-based strands and complementary and non-complementary strands modified with guest molecules and the investigation of their complexation behavior towards each other by isothermal titration calorimetry (ITC). As complementary binding motifs *n*-butyl and α-CD and adamantane and β-CD were selected. It was found that it is possible to realize sequence-specific molecular recognition by the use of host–guest chemistry, but the recognition motifs as well as the linkages have to be chosen very carefully. In the case of trivalent systems one adamantane moiety must be included to induce preferred formation of 1:1 adducts. Due to the too weak interaction between *n*-butyl and α-CD these systems have a negative chelate cooperativity and open adducts are preferentially formed. As soon as two adamantane moieties are present, the complementary systems have a positive chelate cooperativity and double-stranded structures are favored over open adducts. In this system the *n*-butyl moiety provides insufficient discrimination towards α- and β-CD and no sequence specificity is observed. By the combination of three adamantane moieties sequence specificity can be generated. Exclusively with the complementary CD sequence double-stranded structures are formed, with non-complementary strands aggregates of higher stoichiometry are generated.

## Introduction

Multivalency is the interaction of a receptor and a ligand with at least two recognition motifs on each binding partner [1]. In recent years multivalency has been recognized to play a major role in almost all biological processes, e.g., the recognition of cells by other cells, bacteria or viruses, the adhesion of cells or signal transduction pathways [2]. By the combination of multiple, rather weak non-covalent interactions stable yet reversible systems are generated, which are responsive to external stimuli. These advantages have made synthetic multivalent systems interesting for a broad field of applications. In the case of medicinal applications multivalent molecules have been used as inhibitors of toxins or viruses and for imaging and targeted drug delivery [3]. Hydrogels which are built up by multivalent host–guest interactions and vesicles of amphiphilic host molecules have been intensively studied for their ability to function as drug delivery systems as well [4–9]. Additionally, such vesicles can be modified with bio-active ligands and serve as model systems to mimic biological processes on cell membranes [10–11]. In the field of materials science multivalency has been used to create functional polymers [12–14] and self-assembled electronic [15–20] or biofunctional materials [21–27]. Even the molecular recognition of macroscopic gel blocks by multivalent host–guest interactions has been realized [28–32].

Besides the number of receptor–ligand interactions their spatial distribution is crucial for the highly selective molecular recognition as well. The most important natural example of sequence specific, multivalent molecular recognition is the hybridization of complementary DNA strands via the base pairing of adenosine and thymine respectively guanine and cytosine. Within the last years these binding motifs have been transferred to artificial systems like peptide nucleic acids (PNA) [33] and extensively used to mimic biological processes [34–35] or to generate functional materials [36]. Host–guest chemistry has been studied in the field of sequence-specific molecular recognition as well. The selective recognition of short peptides made of natural amino acids with aromatic side chains by different host moieties like coordination cages [37] and cucurbiturils [38–39] has been demonstrated. For cyclodextrins (CD) a similar approach is reported, but by using CD strands and different model peptides of natural and artificial amino acids no significant selectivity was observed [40]. In this work we present an alternative approach to realize the hybridization of complementary strands mediated by multivalent host–guest interaction. We used α- and β-CD because of their well-known and regiospecific modifiability for the preparation of di- and trivalent host sequences and investigated their binding behavior towards complementary and non-complementary di- and trivalent guest sequences which were modified with *n*-butyl and 1-adamantyl moieties. Such structures can be used for the self-assembly of complicated molecular architectures. Furthermore, the results foster the understanding of the basic principles of sequence-specific molecular recognition, which is ubiquitous in nature.

## Results and Discussion

**Figure 1:**
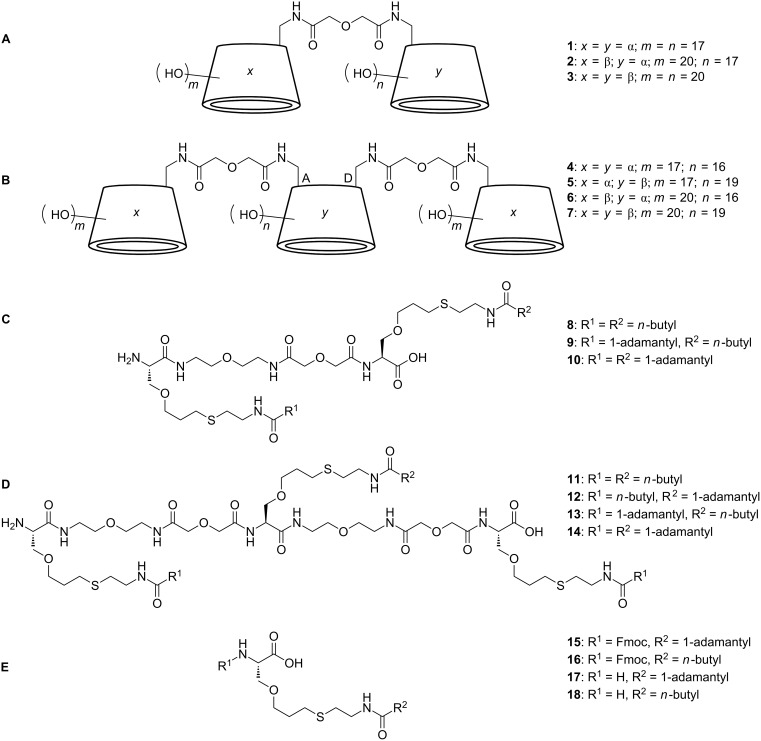
Chemical structures of the di- (A) and trivalent (B) CD sequences, the di- (C) and trivalent (D) guest sequences and the monomeric serine derivatives (E).

**Figure 2:**
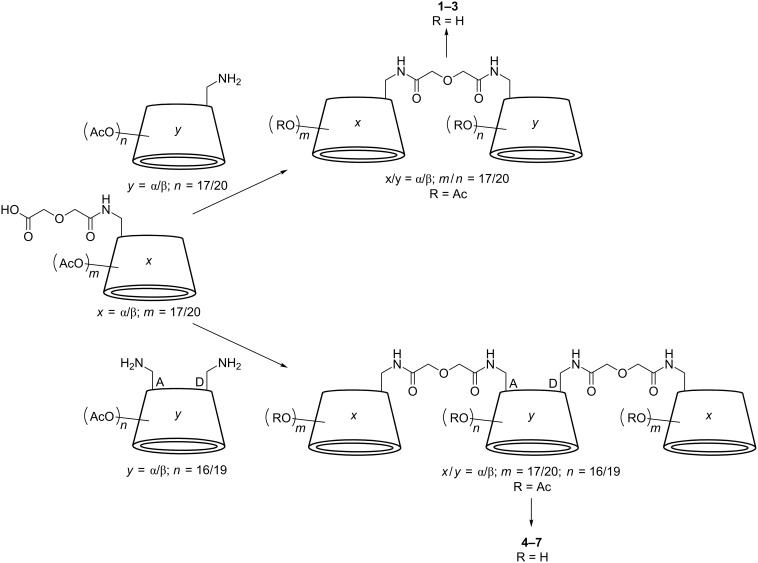
Synthesis of the di- and trivalent CD sequences **1**–**7** (for detailed reaction conditions see Supporting Information File 1).

**Figure 3:**
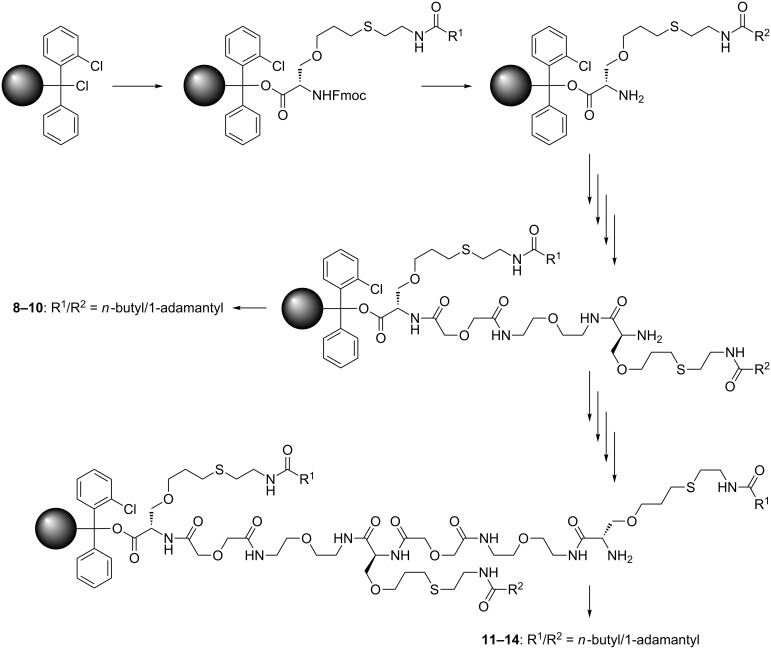
Solid phase peptide synthesis of the di- and trivalent guest strands **8** – **14 (**for detailed reaction conditions see Supporting Information File 1).

The divalent CD sequences **1**–**3** (Figure 1A) were synthesized by the amide coupling of peracetylated α- and β-CD, bearing an amine respectively a carboxylic acid function at the primary side, followed by complete deprotection under Zemplén conditions (Figure 2). The trivalent CD sequences **4**–**7** (Figure 1B) were prepared by amide coupling of peracetylated 6*^A,D^*-diamine functionalized α- and β-CD with monocarboxylic acid functionalized α- and β-CD, again followed by complete deprotection under Zemplén conditions (Figure 2). Based on MALDI mass spectra of the protected and unprotected cyclodextrin strands impurities by monomeric building blocks respectively dimeric species in the case of trivalent strands can be excluded (see Supporting Information File 1). The di- and trivalent guest strands **8**–**14** (Figure 1C, D) were synthesized by solid phase peptide synthesis using a standard Fmoc-protocol (Figure 3). Therefore the serine derivatives **15** and **16** (Figure 1E) and a water-soluble linker molecule were used. The purity of the guest strands is estimated to be >95% based on ^1^H NMR spectra (see Supporting Information File 1). The syntheses of the multivalent host and guest strands are described in detail in Supporting Information File 1.

**Figure 4:**
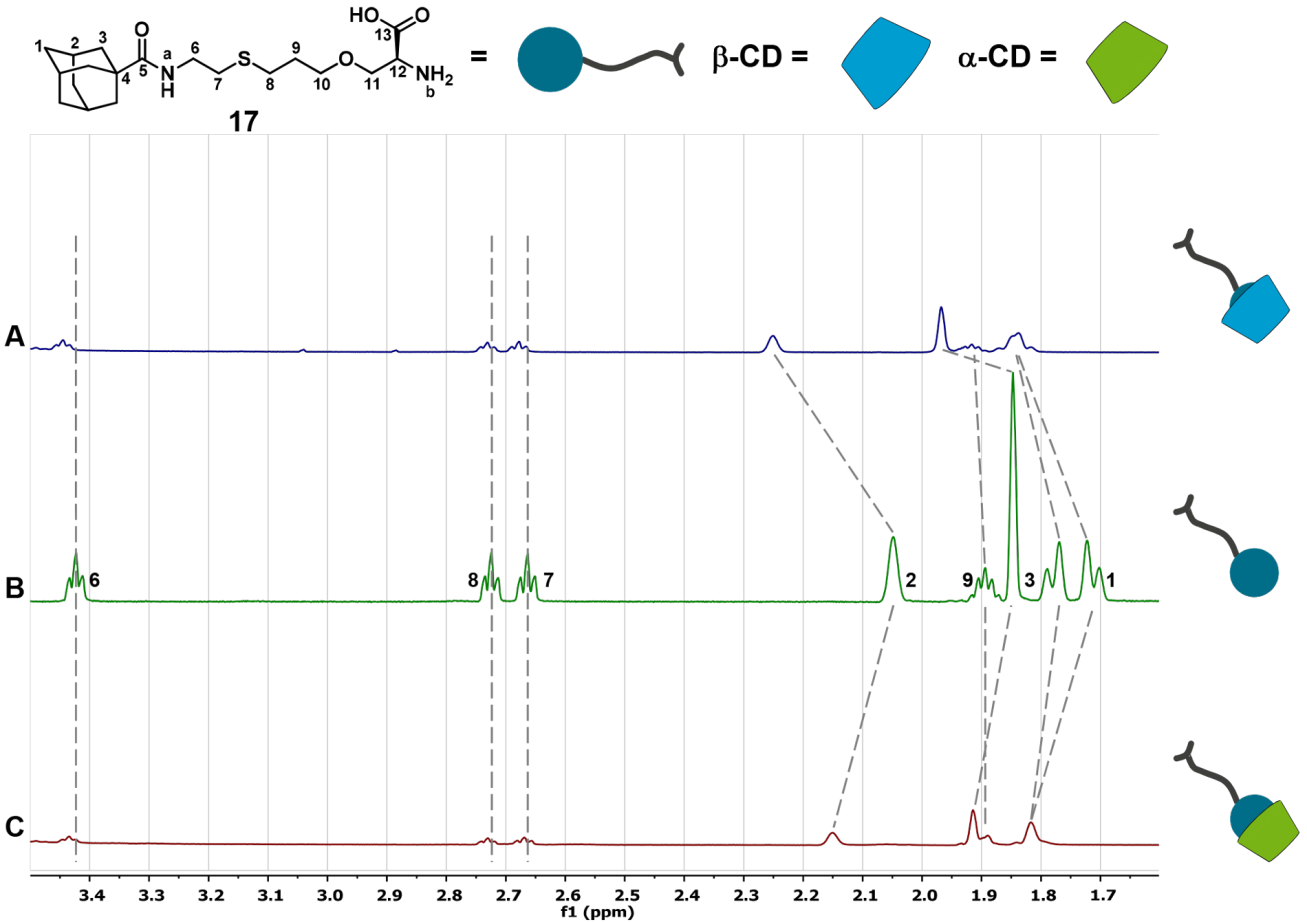
^1^H NMR spectra of **17** (B) and its inclusion complexes with β- (A) and α-CD (C), measured in D_2_O (600 MHz, 25 °C). A) [17] = 1.0 mM, [β-CD] = 10 mM. B) [17] = 1.0 mM. C) [17] = 1.0 mM, [α-CD] = 10 mM.

**Figure 5:**
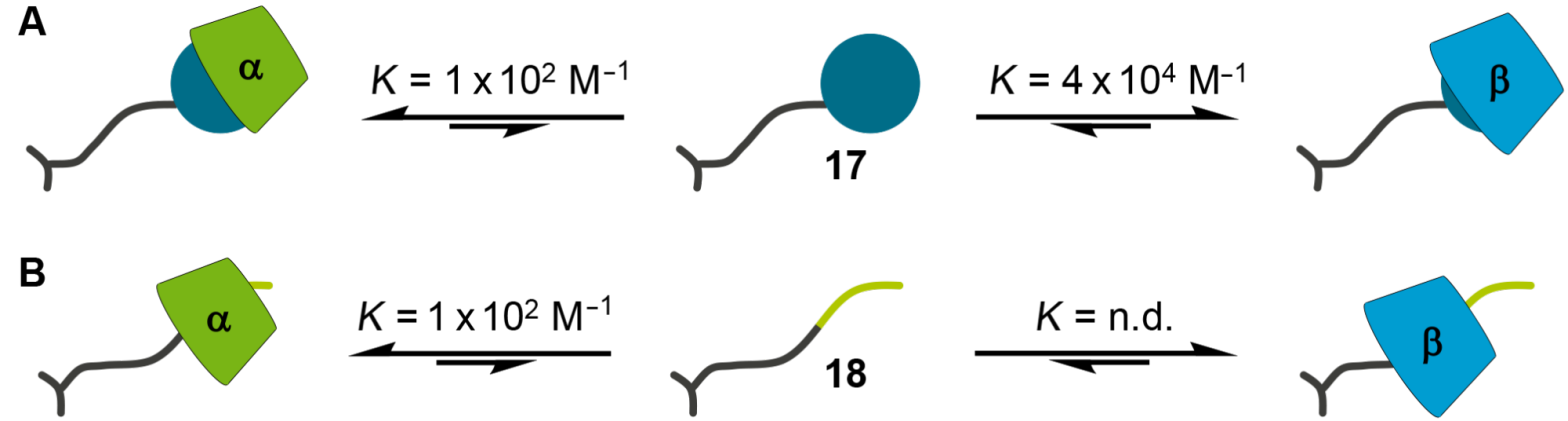
Schematic drawing of the complexation of the serine derivatives **17** (A) and **18** (B) with α- and β-CD. The shown binding constants were determined by ITC, the structures by NMR. n.d. = not detectable by ITC.

First of all, the selectivity of the complexation of the unprotected serine derivatives **17** and **18** towards α- and β-CD was investigated by ITC experiments. The structures of the host–guest complexes were elucidated by NMR spectroscopy. The 1-adamantane-functionalized serine **17** shows complexation of α- and β-CD, forming 1:1 complexes. In both cases the adamantane moiety binds into the CD cavity, which is confirmed by NMR measurements of **17** and 1:10 mixtures of **17** with α- and β-CD (Figure 4). After addition of 10 equivalents of α- or β-CD the signals of the adamantane’s protons are significantly shifted to higher ppm values compared to the signals of pure **17**. The other signals show almost no variation. The interaction of **17** with β-CD is enthalpically as well as entropically favored, while the interaction of **17** with α-CD is exclusively driven by the complexation enthalpy (Table 1). All thermodynamic data are in agreement with literature-known data of comparable systems [41]. Because the interaction between **17** and β-CD has a ca. 400-fold higher binding constant than the interaction of **17** and α-CD the preferred complexation behavior towards β-CD can be expected (Figure 5A).

**Table 1:** Thermodynamic parameters of the host–guest interactions of the serine derivatives **17** and **18** with α- and β-CD.

Serine derivative	**17**		**18**	
CD	α-CD	β-CD	α-CD	β-CD

*n*	1.00	0.94	1.00	–
*K* [M^−1^]	1.06 × 10^2^	3.97 × 10^4^	9.71 × 10^1^	–
Δ*G* [kJ/mol]	−11.6	−26.2	−11.3	–
Δ*H* [kJ/mol]	−19.7	−24.3	−14.9	–
Δ*S* [J/molK]	−27.5	6.5	−11.9	–

**Figure 6:**
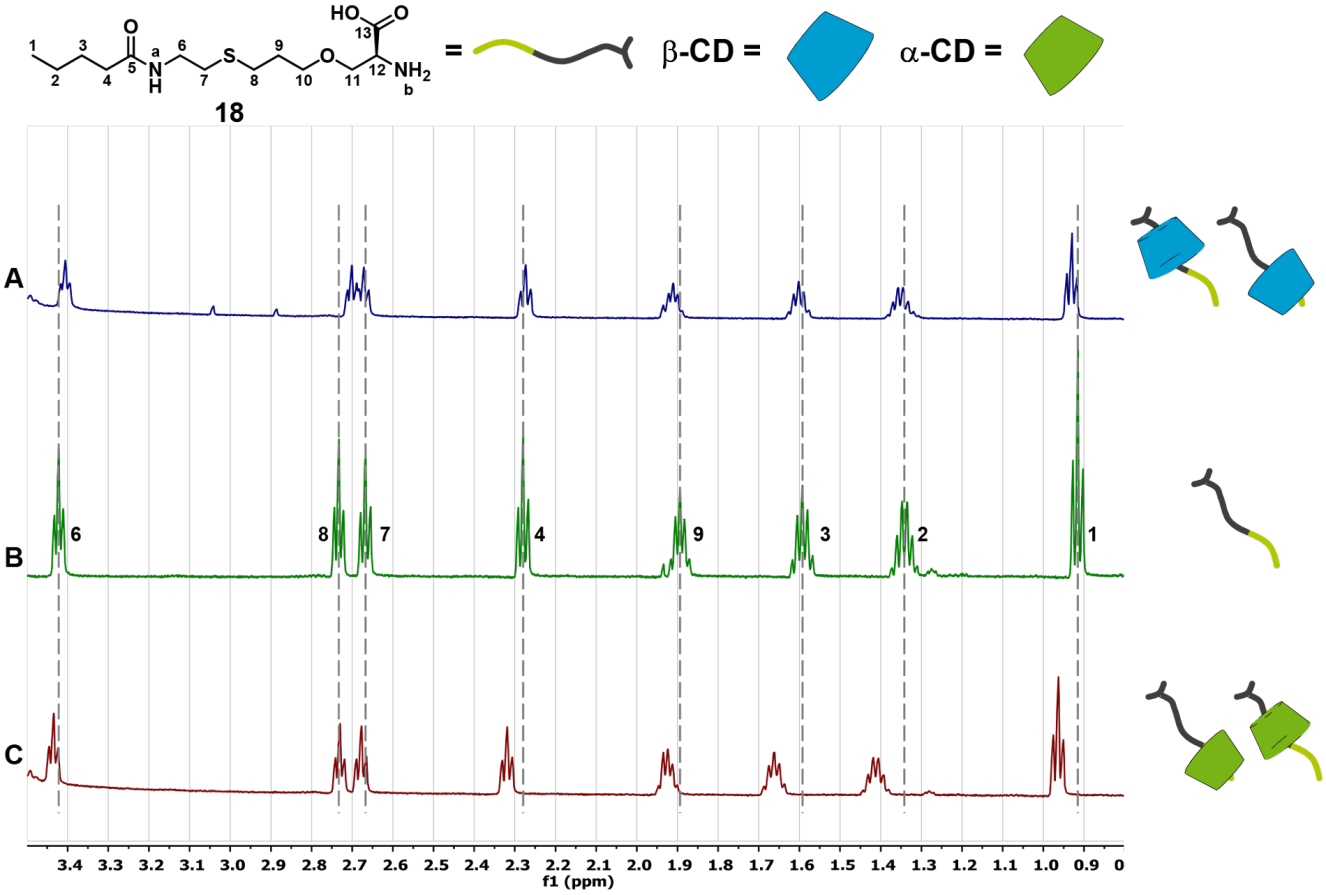
^1^H NMR spectra of **18** (B) and its inclusion complexes with β- (A) and α-CD (C), measured in D_2_O (600 MHz, 25 °C). A) [18] = 1.0 mM, [β-CD] = 10 mM. B) [18] = 1.0 mM. C) [18] = 1.0 mM, [α-CD] = 10 mM.

The *n*-butyl derivate **18** also interacts with both α- and β-CD. In the case of α-CD the formation of a 1:1 adduct with a binding constant of ca. 10^2^ M^−1^ is observed. The complexation is driven by a negative complexation enthalpy (Table 1) and leads to the inclusion of the *n*-butyl moiety of **18**. This is confirmed by NMR spectra, where the protons of the *n*-butyl unit show strong shifting to higher ppm values after addition of 10 equivalents of α-CD (Figure 6B and C). The interaction of **18** with β-CD cannot be quantified based on the ITC measurement with 1 mM of **18** and 10 mM of β-CD. Nevertheless, the NMR spectra of the 1:10 mixture of **18** and β-CD show small variations in the chemical shifts of the protons compared to the spectra of pure **18**, giving a strong hint that complexation takes place (Figure 6A and B). Taking the results of both experiments into account, a binding constant lower than 10^2^ M^−1^ can be assumed for the interaction between **18** and β-CD. Additional measurements with 10-fold increased concentrations of both **18** and β-CD, which is assumed to be necessary to determine binding constants lower than 10^2^ M^−1^, were not possible because of too low solubility of the components. Thereby, the preferred binding of **18** towards α-CD is observed (Figure 5B).

All in all, the monovalent guest molecules **17** and **18** show discrimination towards α- and β-CD in their complexation behavior: **17** prefers to complex β-CD, **18** prefers to complex α-CD.

**Figure 7:**
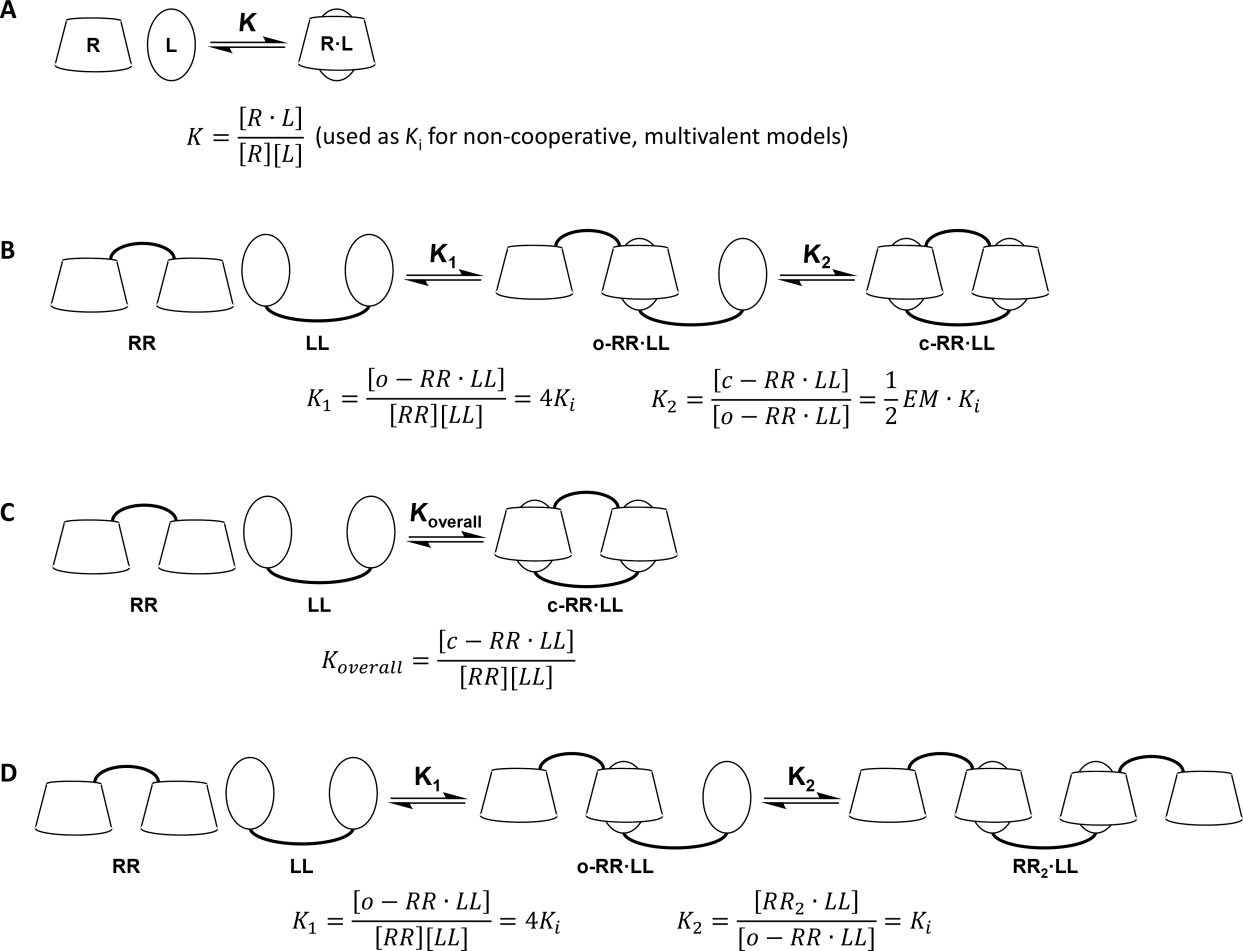
Selected binding models for the analysis of ITC data. (A) Monovalent receptor (R)-ligand (L) interaction. (B) Multivalent interaction of a divalent receptor (RR) and a divalent ligand (LL). (C) 1:1 overall interaction of a divalent receptor (RR) and a divalent ligand (LL). (D) 2:1 interaction of a divalent receptor (RR) and a divalent ligand (LL). EM = effective molarity. See Supporting Information File 2 for details.

In the next step the divalent guest strands **8**, **9** and **10** were investigated regarding their complexation behavior towards the CD dimers **1**, **2** and **3**. Analysis of the ITC data was done using different binding models, based on the host–guest stoichiometry of each system (Figure 7 and Supporting Information File 2) [42–43].

**Figure 8:**
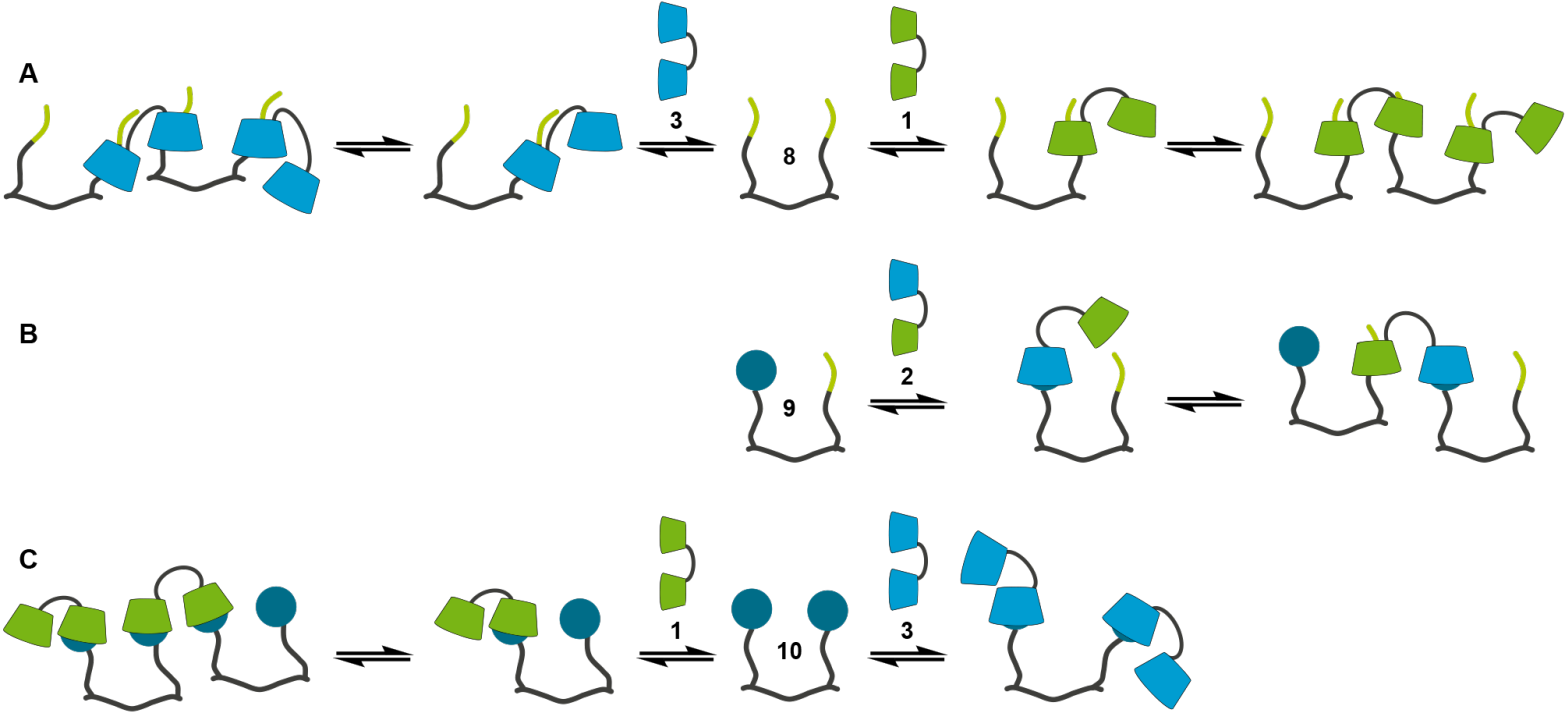
Schematic drawing of the interactions of the divalent guest molecules **8** (A), **9** (B) and **10** (C) with complementary (right) and non-complementary (left) divalent CD strands.

The doubly *n*-butyl substituted strand **8** forms with both the complementary α–α dimer **1** and the non-complementary β–β dimer **3** 1:1 aggregates. Analysis of the ITC data with a multivalent binding model gives effective molarities (EM) of 0.33 mM for the system **1**/**8** and 0.22 mM for the system **3**/**8** (Table 2). In combination with the intrinsic binding constants *K*_i_ of both systems the specific chelate cooperativities can be calculated by multiplication of EM and *K*_i_. With these values a decision about the structures of the 1:1 aggregates, which can exist as open aggregates or closed cyclic systems, can be made. For the system **1**/**8** the intrinsic binding constant is taken from the interaction between **18** and α-CD and set to 10^2^ M^−1^. In case of the system **3**/**8** the monovalent interaction between **18** and β-CD cannot be quantified by ITC measurements. Therefore, the binding constant was overestimated to be 10^2^ M^−1^ as well. Based on these assumptions the chelate cooperativity is 0.03 for the system **1**/**8** and 0.02 for the system **3**/**8**. Both values are significantly lower than 1. This indicates negative chelate cooperativity and preferred formation of open 1:1 adducts for both systems. Depending on the concentration of the host and the guest strands supramolecular polymerization can occur in these systems (Figure 8A). Additionally, the ITC data of the systems **1**/**8** and **3**/**8** were analyzed by a 1:1 overall binding model. Here all complexation steps are combined in one set of thermodynamic parameters. With this method similar binding constants and thermodynamic parameters are calculated for both interactions, so that no sequence-specificity in the complexation behavior of the divalent guest strand **8** is observed (Table 2).

**Table 2:** Thermodynamic parameters of the interactions of the divalent guest strands **8**, **9** and **10** with complementary and non-complementary divalent CD strands.

guest strand	**8**		**9**	**10**	
CD strand	**1**	**3**	**2**	**1**	**3**

*n*	1.00	1.00	1.06	1.00	2.37
*K* [M^−1^]	3.05 × 10^2 a^	3.69 × 10^2 a^	3.11 × 10^4 a^	3.12 × 10^2 a^	1.69 × 10^5 b^
Δ*G* [kJ/mol]	−14.2 ^a^	−14.7 ^a^	−25.6 ^a^	−14.2 ^a^	−29.8 ^b^
Δ*H* [kJ/mol]	−13.3 ^a^	−4.2 ^a^	−25.5 ^a^	−12.0 ^a^	−16.5 ^b^
Δ*S* [J/molK]	3.0 ^a^	35.0 ^a^	0.3 ^a^	7.5 ^a^	44.7 ^b^
EM [mM]	0.33 ^c^	0.22 ^c^	0.25 ^d^	0 ^c^	–

^a^1:1 overall binding model, ^b^2:1 binding model, intrinsic value, ^c^multivalent binding model, ^d^estimated value.

The heterodivalent guest strand **9** forms a 1:1 aggregate with the complementary CD strand **2**. Due to the combination of two different recognition motifs in this system the multivalent analysis of the ITC data is more complicated than for the previously discussed homodivalent systems. Instead of calculating the EM it was estimated to be 0.25 mM because of the structural similarities of the system **2**/**9** with the systems **1**/**8** and **3**/**8**. With a view to the much higher binding constant of **17** towards β-CD compared to all other possible host–guest interactions, it is obvious that this inclusion complex is formed first. In the second step the intramolecular complexation between the *n*-butyl moiety of **9** and the α-CD of **2**, (*K*_i_ ≈ 10^2^ M^−1^) has to appear to build a double stranded structure. Here again a negative chelate cooperativity is present (*K*_i_·EM ≈ 0.03), preventing the intramolecular complexation. The formation of open 1:1 aggregates is further confirmed by the analysis of the ITC data by a 1:1 model of overall complexation. The overall binding constant as well as the overall thermodynamic parameters of the 1:1 analysis are very similar to the values observed for the complexation of **17** with β-CD (Table 2). Therefore, the interaction between **2** and **9** is only based on the interaction between the adamantane moiety of **9** and the β-CD of **2**. The interaction between the *n*-butyl moiety and the α-CD is negligible. As already mentioned for the systems **1**/**8** and **3**/**8** in the system **2**/**9** supramolecular polymerization is possible, depending on the concentrations of the host and the guest strand (Figure 8B).

The homodivalent guest strand **8** with two adamantane moieties shows an alternating complexation behavior towards the complementary CD dimer **3**. The ITC data suggest that instead of 1:1 adducts 2:1 host–guest systems are formed. Therefore, the analysis was done using a 2:1 binding model, assuming two non-cooperative and independent complexations. This method gives intrinsic thermodynamic parameters (Table 2). The intrinsic binding constant is ca. 2 × 10^5^ M^−1^. This value is 4-fold higher than the binding constant between **17** and β-CD, caused by the symmetry effect of the interaction between two homodivalent systems [1]. The intrinsic complexation enthalpy and entropy show that both complexations are enthalpically as well as entropically driven. The interaction of **8** with the non-complementary CD dimer **1** results in the formation of open 1:1 adducts. Analysis of the corresponding ITC data with a multivalent binding model yields an EM of 0, indicating that no intramolecular complexation takes place. Analysis of the data with a 1:1 overall binding model gives a binding constant of ca. 3 × 10^2^ M^−1^, which is higher than the binding constant between **17** and α-CD due to the symmetry effect. The complexation enthalpy and entropy differ from the monovalent interaction α-CD/**17** because of the bridging of the host and the guest molecules (Figure 8C).

In summary, for none of the divalent strands sequence-specific binding can be observed. This is due to negative chelate cooperativities of the systems which are caused by too low intrinsic binding constants on the one hand and too low EM on the other hand. While the intrinsic binding constant can easily be increased by the variation of the guest moieties, especially for the interaction with α-CD, the EM cannot be increased that easy. Here the linkers of the guest and the host moieties have a crucial influence on the structure of the divalent molecules and the host–guest complexes before the intramolecular complexation [1]. Changes in the linkers’ structures can result in different steric environments during the interactions, but the exact effects cannot be predicted and have to be solved by theoretical calculations and simulations.

**Figure 9:**
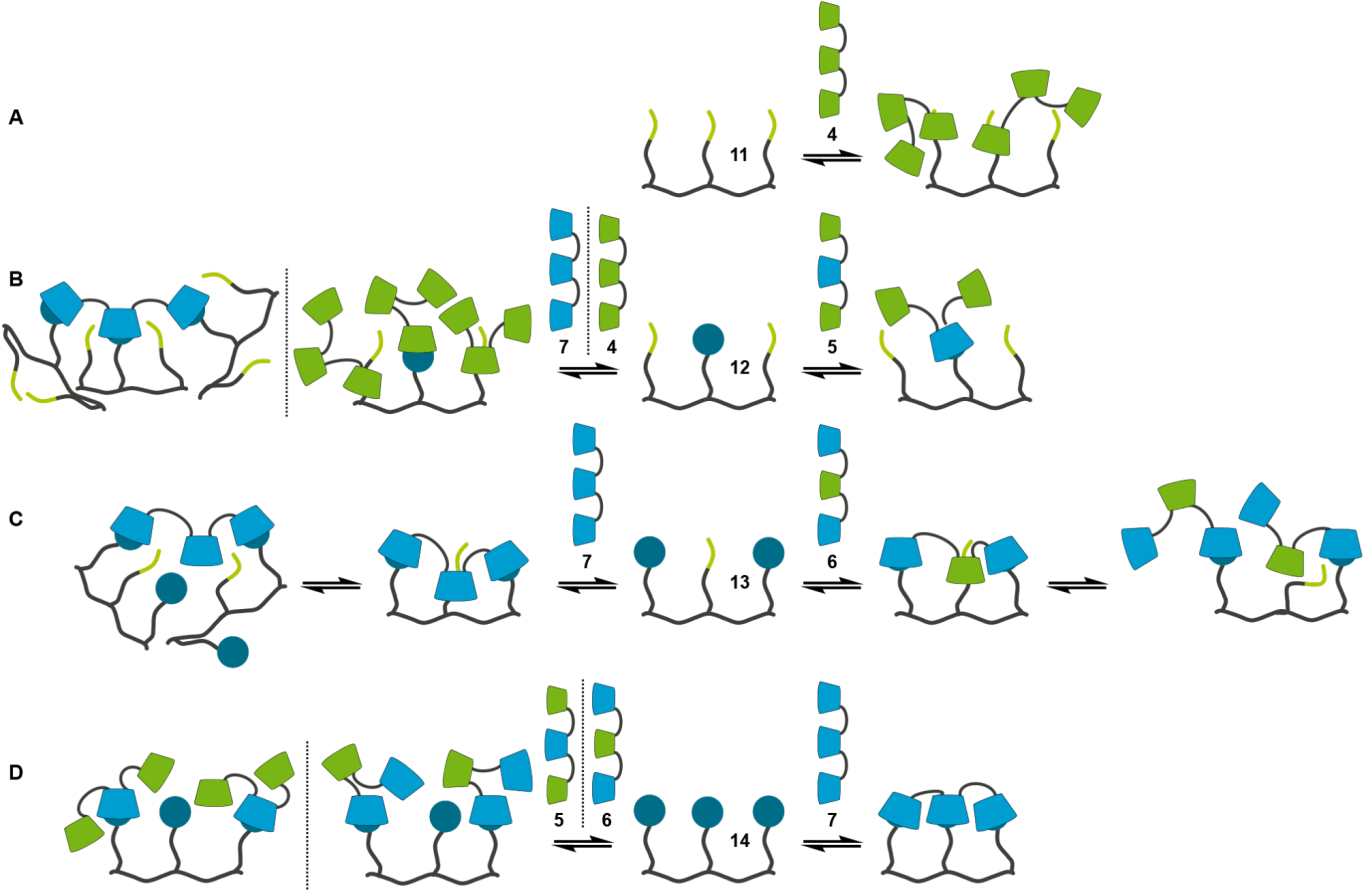
Schematic drawing of the interactions of the trivalent guest molecules **11** (A), **12** (B), **13** (C) and **14** (D) with complementary (right) and non-complementary (left) trivalent CD strands.

In the last step the interactions of the trivalent guest strands **11**, **12**, **13** and **14** with complementary and non-complementary trivalent CD strands were investigated. The three times *n*-butyl-substituted guest strand **11** forms a 2:1 host–guest adduct with the complementary CD strand **4**. The intrinsic binding constant and the intrinsic thermodynamic parameters (Table 3), taken from a 2:1 binding model, are in agreement with the values of the monomeric interaction between **18** and α-CD. This indicates that the 2:1 host–guest adduct is formed by two independent, non-cooperative interactions between two *n*-butyl moieties of **11** and α-CD each of one CD trimer **4** (Figure 9A). The interaction of **11** with the non-complementary CD strand **7** leads to a host–guest aggregate which cannot be characterized by the data of the corresponding ITC measurement.

**Table 3:** Thermodynamic parameters of the interactions of the trivalent guest strands **11**, **12**, **13** and **14** with complementary and non-complementary divalent CD strands.

guest strand	**11**	**12**			**13**		**14**	
CD strand	**4**	**5**	**4**	**7**	**6**	**7**	**7**	**6**

*n*	2.38	0.93	2.70	0.31	1.22	0.77	1.21	2.10
*K* [M^−1^]	1.56 × 10^2 a^	2.53 × 10^4 b^	2.23 × 10^3 c^	1.74 × 10^4 d^	3.91 × 10^5 b^	5.47 × 10^5 b^	1.39 × 10^6 b^	5.26 × 10^4 a^
Δ*G* [kJ/mol]	−12.5 ^a^	−25.1 ^b^	−19.1 ^c^	−24.2 ^d^	−31.9 ^b^	−32.7 ^b^	−35.1 ^b^	−26.9 ^a^
Δ*H* [kJ/mol]	−15.0 ^a^	−27.7 ^b^	−2.6 ^c^	−24.3 ^d^	−35.3 ^b^	−52.8 ^b^	−57.4 ^b^	−36.0 ^a^
Δ*S* [J/molK]	−8.5 ^a^	−8.6 ^b^	55.4 ^c^	−0.5 ^d^	−11.4 ^b^	−67.5 ^b^	−75.1 ^b^	−30.4 ^a^
EM [mM]	–	0.25 ^e^	–	–	0.07 ^f^	0.12 ^f^	0.25 ^e^	–

^a^2:1 binding model, intrinsic value, ^b^1:1 overall binding model, ^c^3:1 binding model, intrinsic value, ^d^1:3 binding model, intrinsic value, ^e^estimated value, ^f^simplified, multivalent binding model.

For the trivalent guest strand **12** bearing one adamantane moiety the selective formation of a 1:1 host–guest adduct with the complementary CD strand **5** is observed. Because of the complexity of a multivalent binding model for a heterotrivalent system the EM of the interaction between **5** and **12** was not calculated but estimated from the structural similar divalent systems **1**/**8** and **3**/**8** and set to be 0.25 mM. Taking into account the complexation behavior of the monovalent systems and the results from the heterodivalent system **2**/**9** it is obvious that during the interaction between **5** and **12** first the complexation between the adamantane of **12** and the β-CD of **5** takes place. The following intramolecular complexation of one of the *n*-butyl moieties by one of the α-CD (*K*_i_ ≈ 10^2^ M^−1^) does not occur due to a negative chelate cooperativity (*K*_i_·EM ≈ 0.03). Therefore only one inclusion complex is formed, leading to an open 1:1 adduct of **5**/**12**. This assumption is confirmed by the modeling of the ITC data with a 1:1 overall binding model. Here a binding constant of ca. 3 × 10^4^ M^−1^ is calculated. This value as well as the overall thermodynamic parameters (Table 3) are in good agreement with the values of the interaction between **17** and β-CD. In contrast to the 1:1 adduct of the complementary strands **12** forms a 3:1 host–guest system with the non-complementary CD strand **4** and a 1:3 host–guest system with the non-complementary CD strand **7**. The ITC data of the interaction between **4** and **12** can be modeled with a 3:1 model assuming that three independent, non-cooperative complexations with the same intrinsic binding constant occur (Table 3). With a view to the almost identical binding constants between α-CD and **17** respectively **18** the assumption of same binding constants for the interactions between α-CD and the adamantane respectively *n*-butyl moieties of **12** is no limitation and the simplified 3:1 model is a valid approach to analyse the ITC data. The intrinsic binding constant of ca. 2 × 10^3^ M^−1^ is one order of magnitude higher than the comparable monovalent binding constants of α-CD/**17** and α-CD/**18**. This is caused by the symmetry effect, which is even higher due to the negligence of different molecular recognition motifs for the simplified modelling. The thermodynamic parameters surprisingly indicate that every inclusion process is mainly driven by the complexation entropy although four molecules are combined in one aggregate. The analysis of the ITC data of the system **7**/**12** was done with a 1:3 binding model where only the interaction between the adamantane moiety of **12** and the β-CD of **7** was taken into account. This approach yields an intrinsic binding constant and intrinsic thermodynamic parameters (Table 3) which are consistent with the values of the interaction between β-CD and **17**. Therefore, three independent, non-cooperative complexations of the adamantane moieties of three equivalents of **12** by the β-CD of **7** can be assumed (Figure 9B).

The heterotrivalent guest strand **13** with two adamantane moieties is the first strand which shows formation of double-stranded aggregates. The ITC data of the interaction between **13** and the complementary CD strand **6** can be analyzed with a 1:1 overall binding model resulting in stoichiometry of 1.22 and an overall binding constant of ca. 4 × 10^5^ M^−1^ (Table 3). This is one order of magnitude higher than the binding constant between **17** and β-CD, which can be attributed to the combination of two adamantane-β-CD interactions in one value. Taking into account that in the prior discussed heteromultivalent systems the interactions between *n*-butyl moieties and α-CD can be neglected, this is presumably valid for the system **6**/**13** as well. Therefore, the complicated heteromultivalent system can be simplified to be the interaction of a homodivalent guest-strand with two adamantane moieties with a homodivalent CD strand of two β-CD and the ITC data can be analyzed with a multivalent binding model with two non-cooperative complexations. This analysis gives an EM of ca. 0.07 mM. Taking the binding constant between β-CD and **17** as intrinsic value (*K*_i_ ≈ 4 × 10^4^ M^−1^), the system **6**/**13** has a chelate cooperativity around 3. Because this value is greater than 1 a positive chelate cooperativity is observed for the interaction of **6** and **13**, favoring the intramolecular complexation and the formation of a double-stranded structure. For the interaction of **13** and the non-complementary strand **7** the same approach of analyzing the data can be done. The 1:1 overall binding model gives a binding constant of ca. 5 × 10^5^ M^−1^, which is similar to the value of the complementary system **6**/**13**. Neglecting the interaction between the *n*-butyl moiety of **13** and the β-CD of **6** the simplified multivalent binding model can be used for modelling the ITC data, resulting in an EM of 0.12 mM. Thus, the system **7**/**13** shows positive chelate cooperativity as well (*K*_i_·EM ≈ 5) and double-stranded structures are preferentially formed. All in all, the guest strand **13** shows preferred formation of double-stranded 1:1 adducts, but not in a sequence-specific way. This is caused by the too weak discrimination of the *n*-butyl moiety of **13** towards the α- and β-CD units of **6** respectively **7** (Figure 9C).

Finally, the homotrivalent guest strand **14** shows sequence-specific molecular recognition. In combination with the complementary CD strand **7** the ITC data show the formation of 1:1 adducts. These exist preferentially as closed double-stranded structures because of a positive chelate cooperativity. Assuming that the EM of the system **7**/**14** is similar to the EM of the structural related divalent systems (EM ≈ 0.25 mM) and taking the binding constant between **17** and β-CD as intrinsic value (*K*_i_ ≈ 4 × 10^4^ M^−1^) the chelate cooperativity is around 10. This means that the intramolecular complexation, which leads to double-stranded structures, is favored over the formation of open 1:1 adducts. These assumptions are confirmed by the analysis of the ITC data with a 1:1 overall binding model. Here a binding constant of ca. 10^6^ M^−1^ is obtained (Table 3). This is one order of magnitude higher than the overall binding constant of the systems **6**/**13** and **7**/**13** and indicates that three complexations between β-CD and adamantane are combined in one value. In contrast to that both the non-complementary systems **5**/**14** and **6**/**14** show the formation of 2:1 host–guest adducts. The corresponding ITC data can be analyzed using 2:1 models where the complexation of the adamantane moieties by α-CD, which has a much lower binding constant than the interaction between adamantane and β-CD, is neglected. The intrinsic binding constants, which are obtained by this method, are ca. 5 × 10^4^ M^−1^ for the system **6**/**14** and ca. 2 × 10^3^ M^−1^ for the system **5**/**14** (Table 3). The clear difference is caused by statistical as well as steric reasons. In the case of the interaction between **6**, which has two β-CD, and **14** more complexations between one adamantane moiety and one β-CD are possible in comparison to the interaction between **5**, which has only one β-CD, and **14**. This difference influences the symmetry effect and leads to different intrinsic binding constants. Additionally, the steric circumstances of the 2:1 host−guest adducts **5**/**14** and **6**/**14** differ from each other. The CD strand **6** has terminally located β-CD units while the β-CD of **5** is located in the centre of the sequence. Thereby the 2:1 adduct of **6** and **14** can avoid sterical hindrance of the uncomplexed cyclodextrins more easily than the 2:1 adduct of **5** and **14**, resulting in a higher intrinsic binding constant (Figure 9D). These results demonstrate, that the homotrivalent guest strand **14** shows sequence-specificity in its molecular recognition. With the complementary CD strand a closed 1:1 adduct is preferentially formed, with non-complementary CD strands host–guest adducts of higher stoichiometry are favored.

## Conclusion

In this work we present our first successful attempt to realize the sequence-specific multivalent molecular recognition of cyclodextrin sequences and complementary strands modified with guest moieties. To this end we selected the interactions between adamantane and β-CD respectively *n*-butyl and α-CD as molecular recognition motifs. In the case of monovalent interactions the expected discrimination was observed. The adamantine-substituted serine **17** prefers to complex β-CD, while the *n*-butyl modified serine **18** prefers the complexation of α-CD. Going to divalent systems neither the formation of closed 1:1 adducts nor is the desired sequence specific molecular recognition observed due to negative chelate cooperativities of all systems. Finally, the trivalent guest strands show a specific 1:1 interaction with the complementary CD strand as soon as one adamantyl moiety is included. The monoadamantyl substituted guest strand **12** forms an open 1:1 adduct with the complementary CD trimer **5**. Because of negative chelate cooperativity only the inclusion complex between the adamantyl moiety of **12** and the β-CD of **5** is formed. If two adamantyl substituents are present in the guest strands, positive chelate cooperativity is generated. As long as the concentrations of the single strands are lower than the system specific effective molarity 1:1 double-strands are preferentially built up. The diadamantyl-substituted guest strand **13** shows no sequence specific molecular recognition, although double stranded structures are formed. With both the complementary cyclodextrin trimer **6** and the non-complementary cyclodextrin trimer **7** cyclic 1:1-adducts are preferred, because the *n*-butyl substituent of **13** provides no sufficient discrimination in the complexation of α- and β-CD. The trivalent homoadamantyl substituted guest strand **14** shows sequence specific molecular recognition. Cyclic 1:1 adducts are exclusively generated with the complementary cyclodextrin trimer **7**. With non-complementary cyclodextrin trimers host–guest systems of higher stoichiometry are formed. Based on these results further development of the guest strands can be done to create even heteromultivalent systems which show highly selective molecular recognition and can be used for the defined self-assembly of molecular structures.

## Experimental

**Materials:** Throughout this work, chemicals were used as received from Acros Organics (Thermo Fischer Scientific Inc., Waltham, Massachusetts, USA), Aldrich (Sigma-Aldrich Corp., St. Louis, Missouri, USA), Alfa Aesar (Alfa Aesar, Ward Hill, Massachusetts, USA), Carbolution Chemicals (Carbolution Chemicals GmbH, Saarbrücken, Germany), Fluka (Sigma-Aldrich Corp., St. Louis, Missouri, USA), Iris Biotech GmbH (Iris Biotech GmbH, Marktredwitz, Germany), Merck (Merck KGaA, Darmstadt, Germany), Novabiochem (Merck KGaA, Darmstadt, Germany) or Wacker (Wacker Chemie AG, München, Germany). The synthesis and analysis of the molecules **1**–**18** is reported in Supporting Information File 1.

**Methods:** ITC measurements were performed with a TA Instruments Nano ITC Low Volume (Waters Corp., Milford, Massachusetts, USA) with a cell volume of 170 µL using ITCRun Version 2.1.7.0 Firmware version 1.31 (TA Instruments, Waters Corp., Milford, Massachusetts, USA) as software. All titrations were done using a 50 µL syringe and 20 injections of 2.5 µL at a temperature of 25 °C and a stirring rate of 350 rpm. All samples were prepared in 100 mM phosphate buffer pH 7.4 and degassed for 10 minutes before use. The data were analysed using NanoAnalyse Data Analysis version 2.36 (TA Instruments, Waters Corp., Milford, Massachusetts, USA), Microsoft^®^ Excel version 14.07113.5005 as part of Microsoft^®^ Office Professional Plus 2010 (Microsoft Corp., Redmond, Washington, USA) and OriginPro 9.1.0G (OriginLab Corp., Northampton, Massachusetts, USA). Before analysis all data were corrected by substraction of a dilution measurement of the titrated component into pure solvent. Concentrations of the components and all ITC data are shown in Supporting Information File 2.

NMR measurements for elucidation of the structures of the host–guest complexes were recorded on an Agilent DD2 600 (Agilent Technologies, Santa Clara, California, USA). Samples were prepared by dissolving 10 equivalents of CD in the corresponding volume of a 1 mM stock solution of the guest molecules in D_2_O. Analysis of the data was done using MNova 9.0.0 (Mestrelab Research S. L., Santiago de Compostela, Spain). The spectra were referenced to the residual solvent signal.

## Supporting Information

File 1 Synthetic procedures and analytical data of **1–18**.

File 2 ITC Measurements.
