# Supplementary material for: Towards the sequence-specific multivalent molecular recognition of cyclodextrin oligomers
Source: Beilstein J Org Chem. 2014 Oct 20;10:2428–40. doi: 10.3762/bjoc.10.253 (PMC4222322; doi:10.3762/bjoc.10.253)
Supplement: File 1 — Synthetic procedures and analytical data of 1–18. [file Beilstein_J_Org_Chem-10-2428-s001.pdf]

SUPPORTING INFORMATION

Towards the sequence-specific multivalent molecular recognition of cyclodextrin oligomers

Michael Kurlemann and Bart Jan Ravoo*

Organic Chemistry Institute, Westfälische Wilhelms-Universität Münster, Corrensstrasse 40, 48149 Münster, Germany

Email: b.j.ravoo@uni-muenster.de

FILE 1: SYNTHETIC PROCEDURES AND ANALYTICAL DATA OF 1-18

Synthesis

General

Throughout this work, chemicals were used as received from *Acros Organics* (*Thermo Fischer Scientific Inc.*, Waltham, Massachusetts, USA), *Aldrich* (*Sigma-Aldrich Corp.*, St. Louis, Missouri, USA), *Alfa Aesar* (*Alfa Aesar*, Ward Hill, Massachusetts, USA), *Carbolution Chemicals* (*Carbolution Chemicals GmbH*, Saarbrücken, Germany), *Fluka* (*Sigma-Aldrich Corp.*, St. Louis, Missouri, USA), *Iris Biotech GmbH* (*Iris Biotech GmbH*, Marktredwitz, Germany), *Merck* (*Merck KGaA*, Darmstadt, Germany), *Novabiochem* (*Merck KGaA*, Darmstadt, Germany) or *Wacker* (*Wacker Chemie AG*, München, Germany). All reactions under inert conditions were performed according to standard *Schlenk* techniques. Dried solvents were obtained from analytical grade solvents according to standard drying methods as follows: DCM p.a. was distilled over CaH_2, DMF p.a. and toluene were stored over 4 Å molecular sieves. Analytical thin layer chromatography was done on silica coated aluminum foils with fluorescence indicator *Silica gel 60 F254* (*Merck KGaA*, Darmstadt, Germany). Visualization of the spots was done by UV light of 254 nm with a *Dual Wavelength UV Lamp (254 nm and 366 nm)* (*CAMAG*, Muttenz, Switzerland) or by dipping into a basic permanganate solution or a mixture of ethanol and sulfuric acid (90%/10%, v/v) and heating afterwards. Preparative silica gel column chromatography was done using *Geduran® Si 60* (*Merck KGaA*, Darmstadt, Germany) with a grain size of 0.040 – 0.063 mm. For preparative size exclusion chromatography *Sephadex® LH20* (*GE Healthcare*, Chalfont St. Giles, Buckinghamshire, UK) with methanol (p.a.) as eluent or *Bio-Beads® S-X1* (*Bio-Rad Laboratories, Inc.*, Hercules, California, USA) with THF (p.a., distilled before use) as eluent were used. The elongation steps of SPPS were performed with a *SP 400 peptide synthesizer* (*Labortec AG*, Bubendorf, Switzerland). Lyophilization was done using an *Alpha 1-2 LD plus*

freeze dryer (*Martin Christ GmbH*, Osterode, Germany). Before lyophilization all substances were dissolved in ddH_2O and frozen under rotation using liquid nitrogen.

NMR spectra were recorded on one of the following instruments: *Bruker DPX300* (*Bruker Corporation*, Billerica, Massachusetts, USA), *Bruker AV300* (*Bruker Corporation*, Billerica, Massachusetts, USA), *Bruker AV400* (*Bruker Corporation*, Billerica, Massachusetts, USA), *Varian VNMRS 500* (*Varian Inc.*, Palo Alto, California, USA) or *Agilent DD2 600* (*Agilent Technologies*, Santa Clara, California, USA). The data were analysed using *MNova 9.0.0* (*Mestrelab Research S. L.*, Santiago de Compostela, Spain). All measurements were performed at room temperature in deuterated solvents. The chemical shifts δ are given in *ppm* and referenced to the residual solvent peak. The coupling constants are noted in Hz and the observed multiplicities of the signals are labeled as singlet (s), duplet (d), triplet (t), quartet (q), pentet (p), hexet (h), heptet (hept), broad (br) and multiplet (m). Complex structures were further analysed using 2D NMR techniques, namely DEPT, GCOSY, GHSQC and GHMBC spectra. If not otherwise mentioned, all spectra were recorded at 298 K. Electrospray ionization (ESI) mass spectra were measured using a *Quattro LCZ* (*Waters-Micromass*, Manchester, UK) with nanospray inlet or a *LTQ Orbitrap LTQ XL* (*Thermo-Fisher Scientific*, Bremen, Germany) with loop inlet. For exact mass determination by ESI mass spectra a *MicroTof* (*Bruker Daltonics*, Bremen, Germany) with a loop inlet was used. The calibration was done immediately before the measurement using sodium formiate clusters. MALDI mass spectra were recorded on a *Autoflex Speed* (*Bruker Daltonics*, Bremen, Germany). For ionization a *SmartBeam*^{TM} *NdYAG-Laser* with a wavelength of 355 nm was used.

Synthesis of the di- and trivalent cyclodextrin sequences

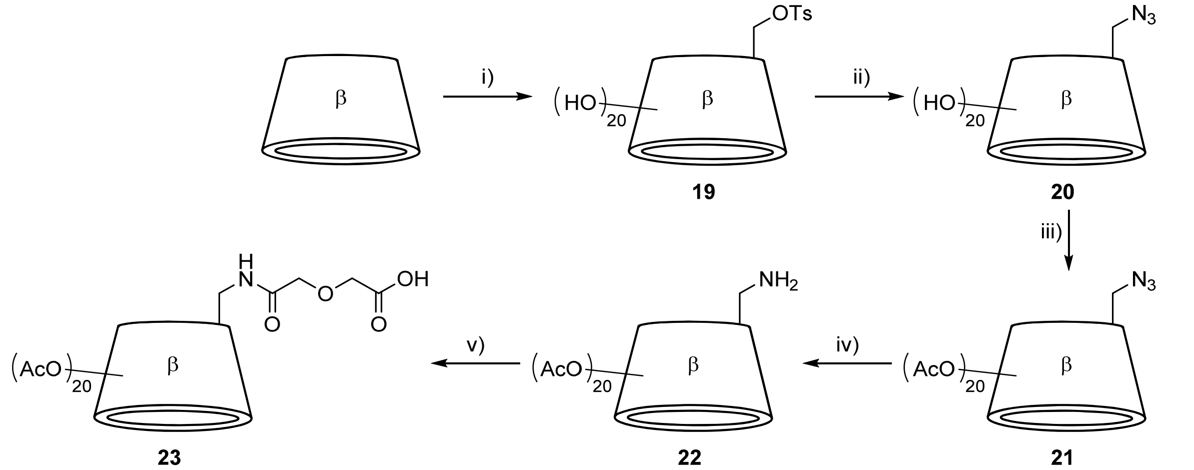

Figure S1: Synthesis of **23**: i) β-CD, *p*-toluenesulfonyl chlorid, NaOH, H_2O, 5 h, -10 °C, 30%; ii) **19**, NaN_3, H_2O, 24 h, 80 °C, 77%; iii) **20**, Ac_2O, pyridine, 17 h, 80 °C, 99%; iv) **21**, Pd/C, H_2, DCM/MeOH, 21 h, r.t., 71%; v) **22**, diglycolic anhydride, DCM, 24 h, r.t., 96%.

19[1], **20**[2] and **21**[3] were prepared as described in literature.

Mono-(2,3-di-*O*-acetyl-6-deoxy-6-amino)-hexakis-(2,3,6-tri-*O*-acetyl)-β-cyclodextrin (**22**)

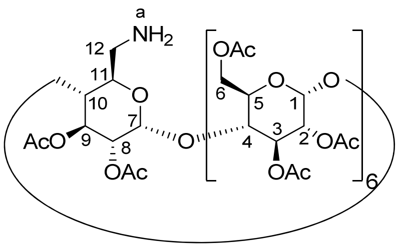

21 (1.01 g, 0.51 mmol) was dissolved in DCM/methanol (50%/50%, v/v, 20 mL). After bubbling argon through the solution for 15 min Pd/C (10% Pd, 250 mg) was added and stirred under H_2 atmosphere for 21 h at room temperature. The suspension was filtered through a pad of *Celite*®, evaporated and purified by column chromatography (silica, EtOAc → EtOAc/MeOH 9:1) to isolate the desired product as white solid.

Yield: 71% (714 mg, 0.36 mmol).

Empirical formula (MW in g/mol): $C_{82}H_{111}NO_{54}$ (1974.73).

R_f-value: 0.40 (silica, EtOAc/MeOH 9:1).

^{1}H NMR (300 MHz, $CDCl_3$) δ 5.34 - 5.22 (m, 7H, H-3/9), 5.18 (d, J = 3.9 Hz, 1H, H-7), 5.13 - 5.00 (m, 6H, H-1), 4.87 - 4.74 (m, 6H, H-2), 4.72 (d, J = 3.8 Hz, 1H, H-8), 4.63 - 4.48 (m, 6H, H-6), 4.33 - 4.19 (m, 6H, H-6'), 4.19 - 4.08 (m, 6H, H-5), 4.01 - 3.82 (m, 2H, H-10, 11), 3.77 - 3.62 (m, 6H, H-4), 3.19 (d, J = 13.8 Hz, 1H, H-12), 3.06 (d, J = 14.2 Hz, 1H, H-12'), 2.19 - 1.96 (m, 60H, CH_3, OAc), 1.24 (t, J = 7.1 Hz, 2H, H-a).

^{13}C{^{1}H} NMR (151 MHz, $CDCl_3$) δ 170.82 - 170.38, 169.61 - 169.46 (C_q, OAc), 96.93, 96.90, 96.84, 96.80, 96.76, 96.73 (C-1/7), 77.06, 76.98, 76.88, 76.82, 76.74, 76.70, 76.65 (C-4/10), 72.18, 71.28, 71.14, 70.96, 70.84, 70.82, 70.78, 70.76, 70.59, 70.51, 70.47, 70.34, 69.71, 69.67, 69.65, 69.63, 69.62, 69.47 (C-2/8, 3/9, 5/11), 62.87, 62.71, 62.68, 62.58, 62.52 (C-6), 41.67 (C-12), 20.89 - 20.81 (CH_3, OAc).

MS-ESI-EM (+) (m/z): Calculated for $[C_{82}H_{111}NO_{54}H]^+$: 1974.6043; found: 1974.6071. Calculated for $[C_{82}H_{111}NO_{54}Na]^+$: 1996.5863; found: 1996.5895.

Mono-(2,3-di-*O*-acetyl-6-deoxy-6-(2-(carboxymethoxy(acetamido))))-hexakis-(2,3,6-tri-*O*-acetyl)-β-cyclodextrin (**23**)

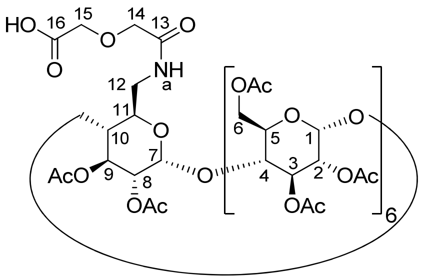

Under argon atmosphere diglycolic anhydride (160 mg, 1.38 mmol) was added to a solution of **22** (593 mg, 0.30 mmol) in DCM (p.a., 7.5 mL) and stirred for 24 h at room temperature. The reaction was quenched by addition of aqueous NH_4Cl (sat., 25 mL). After stirring for 15 min DCM (75 mL) was added and the organic phase was separated and washed with aqueous NH_4Cl (sat.)/brine (75%/25%, v/v, 100 mL) and brine (100 mL), dried over $MgSO_4$, filtered and evaporated. After drying under reduced pressure the pure product was obtained as white solid.

Yield: 96% (604 mg, 0.29 mmol).

Empirical formula (MW in g/mol): $C_{86}H_{115}NO_{58}$ (2090.81).

^{1}H NMR (600 MHz, $CDCl_3$) δ 7.64 (t, J = 6.4 Hz, 1H, H-a), 5.41 - 5.24 (m, 7H, H-3/9), 5.13 (d, J = 4.0 Hz, 1H, H-1/7), 5.11 - 5.01 (m, 6H, 5xH-1/7, 1xH-2/8), 4.96 (d, J = 4.1 Hz, 1H, H-1/7), 4.87 - 4.66 (m, 7H, 6xH-2/8, 1xH-6), 4.62 - 4.42 (m, 6H, 1xH-5/11, 5xH-6), 4.38 - 3.97 (m, 16H, 6xH-5/11, 6xH-6, H-14, 15), 3.92 - 3.79 (m, 3H, 2xH-4/10, H-12), 3.76 - 3.66 (m, 4H, H-4/10), 3.41 (dd, J = 9.2, 5.4 Hz, 1H, H-4/10), 3.13 - 3.04 (m, 1H, H-12'), 2.18 - 1.96 (m, 60H, CH_3, OAc).

^{13}C{^{1}H} NMR (151 MHz, $CDCl_3$) δ 171.98, 171.41 (C-13, 16), 171.21 - 170.52, 169.72 - 169.35 (C_q, OAc), 97.47, 97.36, 97.23, 97.13, 97.00, 96.83, 96.14 (C-1/7), 78.10,

77.62, 76.86, 76.49, 76.42, 76.22 (C-4/10), 72.04, 71.81, 71.57, 71.25, 71.03, 70.93, 70.86, 70.48, 70.41, 70.18, 70.04, 69.80, 69.70, 69.66, 69.45, 69.43, 69.10, 68.92, 67.89 (C-2/8, 3/9, 5/11, 14, 15), 63.42, 63.12, 63.09, 62.99, 62.36, 62.31 (C-6), 41.02 (C-12), 21.01 - 20.71 (CH_3, OAc).

MALDI-MS (+; DHB; EtOAc) (m/z): Calculated for $[C_{86}H_{115}NO_{58}Na]^+$: 2112.60; found: 2112.62.

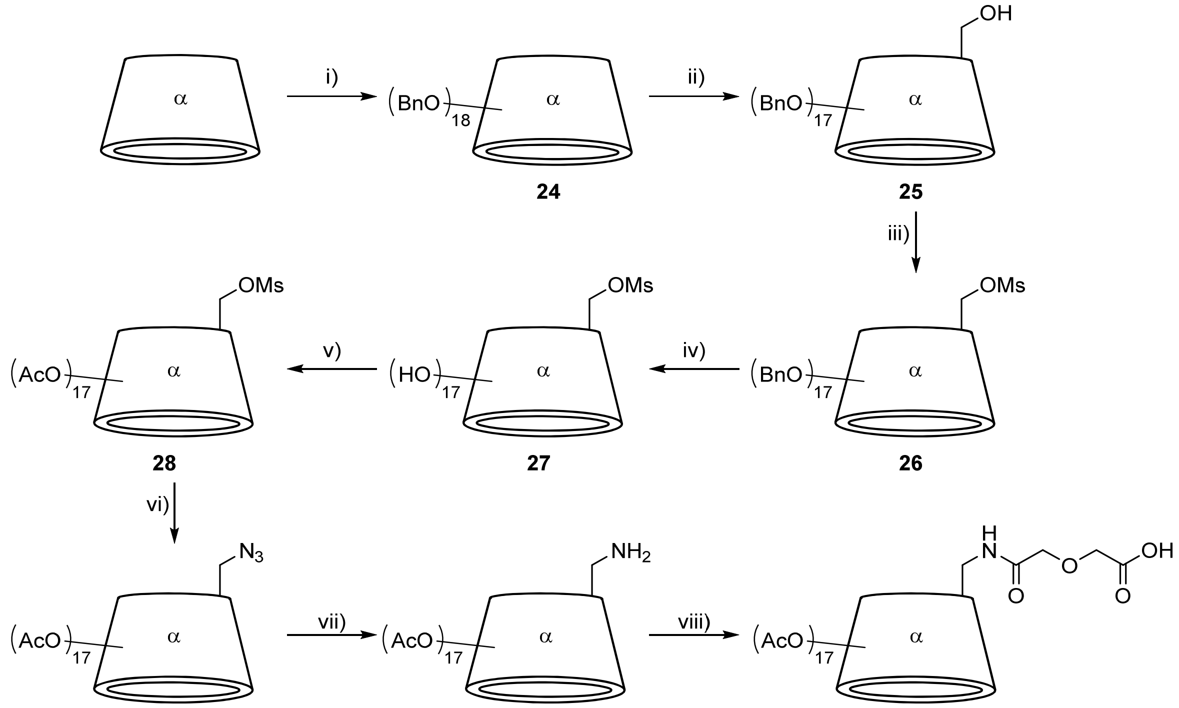

Figure S2: Synthesis von **29**: i) α-CD, NaH, benzyl chloride, DMSO, 22 h, r.t., 99%; ii) **24**, DIBAL-H, 4Å molecular sieves, toluene, 24 h, r.t., 33%; iii) **25**, mesyl chloride, pyridine, DMAP, DCM, 15 min, 0 °C, 18 h, r.t., 93%; iv) **26**, Pd/C, H_2, THF/H_2O/AcOH, 5 d, r.t., quant.; v) **27**, Ac_2O, pyridine, 16 h, 60 °C, 90%; vi) **28**, NaN_3, DMF, 17 h, 60 °C, 75%; vii) **29**, Pd/C, H_2, DCM/MeOH, 16 h, quant.; viii) **30**, diglycolic anhydride, DCM, 24 h, r.t., 89%.

24[4], **25**[5], **26** and **27**[6] were prepared as described in literature.

Mono-(2,3-di-*O*-acetyl-6-*O*-mesyl)-pentakis-(2,3,6-tri-*O*-acetyl)-α-cyclodextrin (**28**)

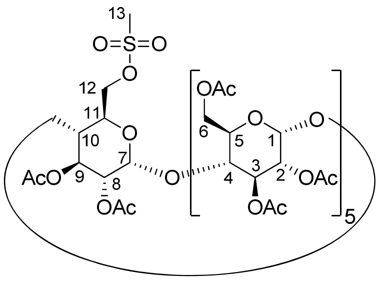

Under argon atmosphere **27** (533 mg, 0.51 mmol) was dissolved in pyridine (p.a., 8 mL) and acetic anhydride (4 mL) and stirred for 16 h at 60 °C. The reaction was quenched by addition of methanol (4 mL) and all volatile components were removed *in vacuo*. The residue was dissolved in DCM (100 mL) and washed with aqueous HCl (1 M, 100 mL), aqueous $NaHCO_3$ (sat., 100 mL) and brine (100 mL), dried over $MgSO_4$, filtered and evaporated. After drying under reduced pressure the product was obtained as light yellow solid.

Yield: 90% (812 mg, 0.46 mmol).

Empirical formula (MW in g/mol): $C_{71}H_{96}O_{49}S$ (1765.56).

^{1}H NMR (600 MHz, $CDCl_3$) δ 5.60 (dd, J = 10.4, 8.6 Hz, 1H, H-3/9), 5.57 - 5.43 (m, 5H, H-3/9), 5.09 - 5.06 (m, 2H, H-1/7), 5.06 - 5.02 (m, 3H, H-1/7), 5.02 (d, J = 3.5 Hz, 1H, H-1/7), 4.84 - 4.71 (m, 6H, H-2/8), 4.61 - 4.52 (m, 2H, H-12), 4.48 - 4.32 (m, 10H, H-6), 4.32 - 4.28 (m, 1H, H-5/11), 4.21 - 4.14 (m, 3H, H-5/11), 4.14 - 4.09 (m, 2H, H-5/11), 3.85 - 3.75 (m, 6H, H-4/10), 3.10 (s, 3H, H-13), 2.20 - 2.00 (m, 51H, CH_3, OAc).

^{13}C{^{1}H} NMR (151 MHz, $CDCl_3$) δ 170.86 - 170.57, 169.46 - 169.27 (C_q, OAc), 96.98, 96.80, 96.76, 96.64, 96.33 (C-1/7), 77.69, 77.48, 76.54 (C-4/10), 71.38, 71.33, 71.19, 71.04, 71.01, 70.92, 70.74, 70.69, 69.88, 69.62, 69.59, 69.51, 69.47, 69.37 (C-2/8, 3/9, 5/11), 68.86 (C-12), 64.04, 63.34, 63.23, 63.18, 63.15, 63.10 (C-6), 37.49 (C-13), 25.38 - 20.85 (CH_3, OAc).

MS-ESI-EM (+) (m/z): Calculated for $[C_{71}H_{96}O_{49}SNa_2]^{2+}$: 905.2263; found: 905.2281. Calculated for $[C_{71}H_{96}O_{49}SNa]^{+}$: 1787.4633; found: 1787.4647.

Mono-(2,3-di-*O*-acetyl-6-deoxy-6-azido)-pentakis-(2,3,6-tri-*O*-acetyl)-α-cyclodextrin (**29**)

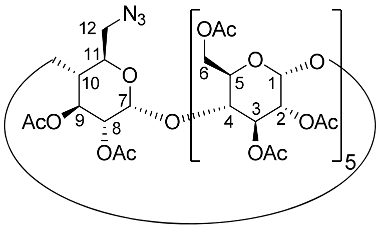

28 (648 mg, 0.37 mmol) and NaN_3 (239 mg, 3.67 mmol) were dissolved in DMF (SPPS grade, 11 mL) and stirred for 17 h at 60 °C. After removing all volatile compounds *in vacuo* the residue was taken up in DCM (75 mL) and dH_2O (75 mL). The organic phase was isolated and the aqueous phase was extracted with DCM (2 x 75 mL). The combined organic phases were washed with brine (100 mL), dried over $MgSO_4$, filtered and evaporated. The desired product was obtained after column chromatography (silica, DCM/MeOH 20:1) as white solid.

Yield: 75% (473 mg, 0.28 mmol).

Empirical formula (MW in g/mol): $C_{70}H_{93}N_3O_{46}$ (1712.48).

R_f-value: 0.30 (silica, DCM/MeOH 20:1).

^{1}H NMR (600 MHz, $CDCl_3$) δ 5.61 - 5.50 (m, 4H, H-3/9), 5.48 - 5.43 (m, 2H, H-3/9), 5.10 (d, *J* = 3.6 Hz, 1H, H-1/7), 5.09 - 5.04 (m, 3H, H-1/7), 5.01 (d, *J* = 3.5 Hz, 1H, H-1/7), 4.95 (d, *J* = 3.5 Hz, 1H, H-1/7), 4.81 - 4.72 (m, 6H, H-2/8), 4.49 - 4.30 (m, 10H, H-6), 4.25 - 4.20 (m, 1H, H-5/11), 4.18 - 4.10 (m, 5H, H-5/11), 3.87 - 3.74 (m, 6H, H-4/10), 3.74 - 3.70 (m, 2H, H-12), 2.20 - 2.13 (m, 15H, CH_3, OAc), 2.10 - 2.00 (m, 36H, CH_3, OAc).

$^{13}C\{^{1}H\}$ NMR (151 MHz, $CDCl_3$) δ 170.82 - 170.42, 169.96 - 169.25 (C_q, OAc), 97.01, 96.85, 96.72, 96.61, 96.58, 96.38 (C-1/7), 78.04, 77.76, 76.88 (C-4/10), 71.40, 71.35, 71.27, 71.19, 71.11, 71.07, 71.01, 70.85, 70.71, 70.54, 70.20, 69.71, 69.47, 69.42, 69.36 (C-2/8, 3/9, 5/11), 63.31, 63.25, 63.20, 63.15 (C-6), 51.50 (C-12), 20.97 - 20.86 (CH_3, OAc).

MS-ESI-EM (+) (m/z): Calculated for $[C_{70}H_{93}N_3O_{46}Na_2]^{2+}$: 878.74073; found: 878.74144. Calculated for $[C_{70}H_{93}N_3O_{46}Na]^{+}$: 1734.49224; found: 1734.49258.

Mono-(2,3-di-*O*-acetyl-6-deoxy-6-amino)-pentakis-(2,3,6-tri-*O*-acetyl)-α-cyclodextrin (**30**)

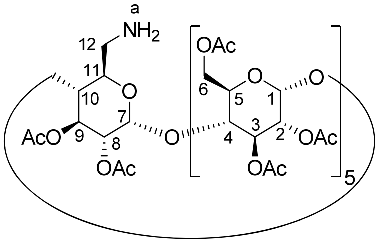

29 (473 mg, 0.28 mmol) was dissolved in DCM/methanol (50%/50%, v/v, 16 mL). After bubbling argon through the solution for 15 min Pd/C (10% Pd, 130 mg) was added and stirred under H_2 atmosphere for 16 h at room tempearture. The catalyst was removed by filtration through a pad of *Celite*® and after evaporation the pure product was obtained as white solid.

Yield: quant. (480 mg, 0.28 mmol).

Empirical formula (MW in g/mol): $C_{70}H_{95}NO_{46}$ (1686.48).

^{1}H NMR (600 MHz, $CDCl_3$) δ 5.69 - 5.37 (m, 6H, H-3/9), 5.29 (d, J = 5.4 Hz, 1H, H-1/7), 5.15 - 4.93 (m, 5H, H-1/7), 4.89 (dd, J = 10.3, 3.3 Hz, 1H, H-2/8), 4.84 - 4.69 (m, 5H, H-2/8), 4.56 - 4.08 (m, 16H, H-5/11, 6), 3.98 - 3.73 (m, 8H, H-4/10, 12), 2.23 - 1.96 (several s, 51H, CH_3, OAc).

$^{13}C\{^1H\}$ NMR (151 MHz, $CDCl_3$) δ 171.39 - 170.52, 169.43 - 169.17 (C_q, OAc), 97.43, 97.14, 96.94, 96.73, 96.57, 96.45 (C-1/7), 79.36, 78.24, 77.95, 77.71, 77.63, 76.64 (C-4/10), 71.81, 71.44, 71.32, 71.16, 71.05, 70.99, 70.90, 70.83, 70.79, 70.64, 70.53, 70.42, 70.22, 69.82, 69.51, 69.45, 69.34, 69.16, 68.70 (C-2/8, 3/9, 5/11), 63.68, 63.50, 63.40, 63.29, 63.22 (C-6), 62.17 (C-12), 21.11 - 20.84 (CH_3, OAc).

MS-ESI-EM (+) (m/z): Calculated for $[C_{70}H_{95}NO_{46}H]^+$: 1686.51980; found: 1686.52335.

Mono-(2,3-di-*O*-acetyl-6-deoxy-6-(2-(carboxymethoxy(acetamido))))-pentakis-(2,3,6-tri-*O*-acetyl)-α-cyclodextrin (**31**)

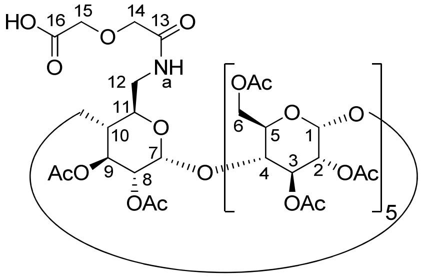

Under argon atmosphere diglycolic anhydride (120 mg, 1.03 mmol) was added to a solution of **30** (394 mg, 0.23 mmol) in DCM (p.a., 5 mL) and stirred for 24 h at room temperature. The reaction was quenched by addition of aqueous NH_4Cl (sat., 25 mL). After stirring for 15 min DCM (75 mL) was added and the organic phase was separated and washed with aqueous NH_4Cl (sat.)/brine (75%/25%, v/v, 100 mL) and brine (100 mL), dried over $MgSO_4$, filtered and evaporated. After drying under reduced pressure the pure product was obtained as white solid.

Yield: 89% (370 mg, 0.21 mmol).

Empirical formula (MW in g/mol): $C_{74}H_{99}NO_{50}$ (1802.55).

^{1}H NMR (600 MHz, $CDCl_3$) δ 7.41 (s, 1H, H-a), 5.62 - 5.40 (m, 7H, H-3/9), 5.18 (d, J = 3.8 Hz, 1H, H-1/7), 5.11 - 4.93 (m, 6H, H-1/7), 4.86 - 4.68 (m, 7H, H-2/8), 4.52 - 3.99 (m, 23H, H-5/11, 6, 14, 15), 3.89 - 3.59 (m, 9H, H-4/10, 12), 2.20 - 1.97 (m, 51H, CH_3, OAc).

$^{13}C\{^{1}H\}$ NMR (151 MHz, $CDCl_3$) δ 171.70 - 170.53, 169.52 - 169.28 (C_q, OAc, C-13, 16), 96.96, 96.69, 96.58, 96.55, 96.45 (C-1/7), 78.93, 77.25, 77.07, 76.78 (C-4/10), 71.40, 71.31, 71.25, 71.20, 71.17, 71.12, 71.06, 70.99, 70.94, 70.90, 70.86, 70.83, 70.78, 70.47, 70.22, 69.67, 69.53, 69.45, 69.41, 69.34, 69.21, 68.65 (C-2/8, 3/9, 5/11, 14, 15), 63.62, 63.48, 63.32, 63.28, 63.23, 63.18 (C-6), 40.33 (C-12), 21.05 - 20.84 (CH_3, OAc).

MS-ESI-EM (+) (m/z): Calculated for $[C_{74}H_{99}NO_{50}Na_2]^{2+}$: 923.75096; found: 923.75169. Calculated for $[C_{74}H_{99}NO_{50}Na]^{+}$: 1824.51270; found: 1824.51380.

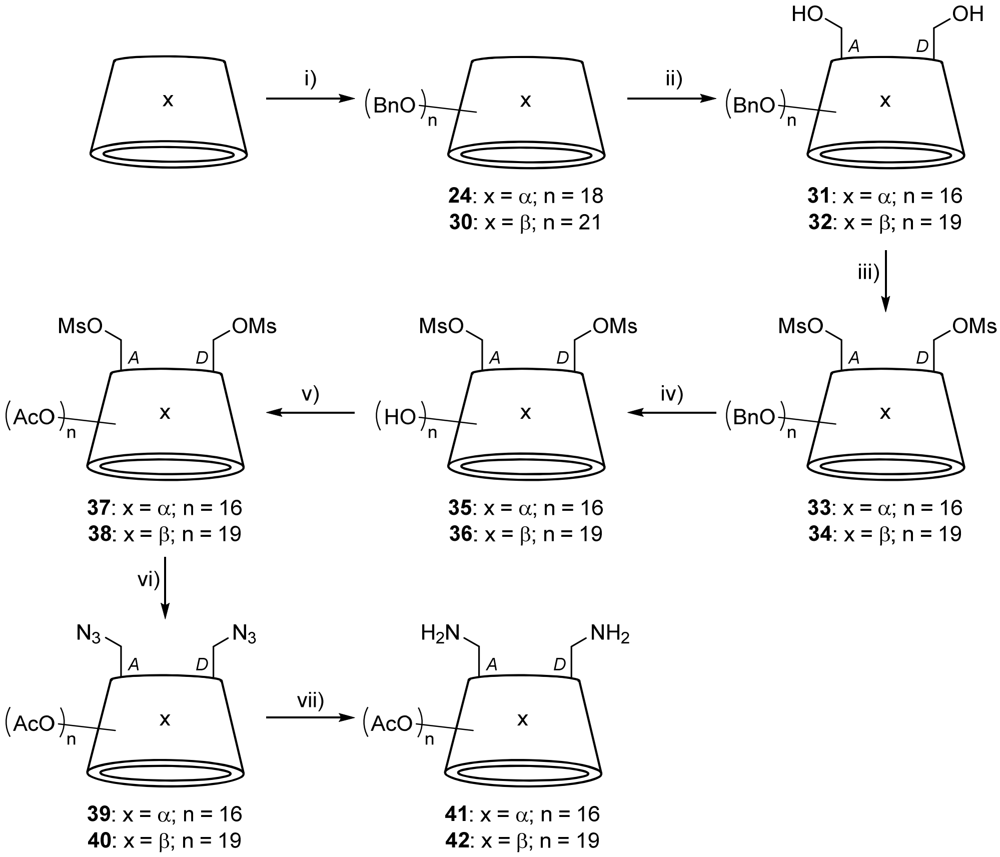

Figure S3: Synthesis of **41** and **42**: i) α-/β-CD, NaH, benzyl chloride, DMSO, 22 h, r.t., 99% (**24**)/99% (**30**); ii) **24**/**30**, DIBAL-H, 4Å molecular sieves, toluene, 24 h, r.t., 33% (**31**)/73% (**32**); iii) **31**/**32**, mesyl chloride, pyridine, DMAP, DCM, 15 min, 0 °C, 18 h, r.t., 87% (**33**)/97% (**34**); iv) **33**/**34**, Pd/C, H_2, THF/H_2O/AcOH, 5 d, r.t., quant. (**35**)/92% (**36**); v) **35**/**36**, Ac_2O, pyridine, 16 h, 60 °C, 87% (**37**)/87% (**38**); vi) **37**/**38**, NaN_3, DMF, 17 h, 80 °C, quant. (**39**)/90% (**40**); vii) **39**/**40**, Pd/C, H_2, DCM/MeOH, 5 d/3 d, r.t., 89% (**41**)/94% (**42**).

24 and **30**[4], **31** and **32**[5], **33** and **34** and **35** and **36**[6] were prepared as described in literature.

A,D-Di-(2,3-di-*O*-acetyl-6-*O*-mesyl)-tetrakis-(2,3,6-tri-*O*-acetyl)-α-cyclodextrin (**37**)

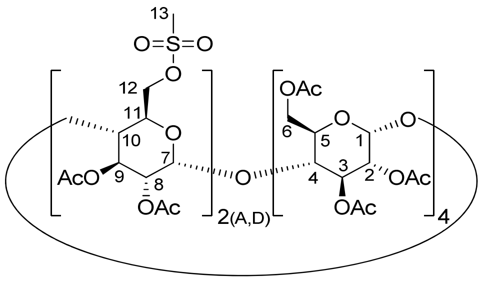

Under argon atmosphere **35** (820 mg, 0.73 mmol) was dissolved in pyridine (p.a., 10 mL) and acetic anhydride (5 mL) and stirred for 16 h at 60 °C. The reaction was quenched by addition of methanol (5 mL) and all volatile components were removed *in vacuo*. The residue was dissolved in DCM (100 mL) and washed with aqueous HCl (1 M, 100 mL), aqueous $NaHCO_3$ (sat., 100 mL) and brine (100 mL), dried over $MgSO_4$, filtered and evaporated. After drying under reduced pressure the product was obtained as light yellow solid.

Yield: 87% (1.15 g, 0.64 mmol).

Empirical formula (MW in g/mol): $C_{70}H_{96}O_{50}S_2$ (1801.61).

^{1}H NMR (600 MHz, $CDCl_3$) δ 5.59 (dd, *J* = 10.3, 8.5 Hz, 2H, H-3/9), 5.53 (dd, *J* = 10.4, 8.7 Hz, 2H, H-3/9), 5.42 (dd, *J* = 10.2, 8.5 Hz, 2H, H-3/9), 5.11 (d, *J* = 3.6 Hz, 2H, H-1/7), 5.04 (d, *J* = 3.5 Hz, 2H, H-1/7), 5.01 (d, *J* = 3.6 Hz, 2H, H-1/7), 4.84 - 4.71 (m, 6H, H-2/8), 4.63 - 4.58 (m, 4H, H-6/12), 4.43 - 4.25 (m, 10H, H-5/11, 6/12), 4.15 - 4.10 (m, 4H, H-5/11), 3.88 - 3.77 (m, 6H, H-4/10), 3.10 (s, 6H, H-13), 2.20 - 1.99 (m, 48H, CH_3, OAc).

^{13}C{^{1}H} NMR (151 MHz, $CDCl_3$) δ 170.96 - 170.50, 169.47 - 169.28 (C_q, OAc), 97.10, 96.84, 96.68 (C-1/7), 77.59, 76.32 (C-4/10), 71.75, 70.93, 70.87, 70.77, 70.52, 69.98, 69.52, 69.31 (C-2/8, 3/9, 5/11), 68.90, 63.17, 63.14 (C-6/12), 37.55 (C-13), 21.04 - 20.85 (CH_3, OAc).

MS-ESI-EM (+) (m/z): Calculated for $[C_{70}H_{96}O_{50}S_2Na_2]^+$: 923.2098; found: 923.2095. Calculated for $[C_{70}H_{96}O_{50}S_2Na]^+$: 1823.4303; found: 1823.4254.

A,D-Di-(2,3-di-*O*-acetyl-6-*O*-mesyl)-pentakis-(2,3,6-tri-*O*-acetyl)-β-cyclodextrin (**38**)

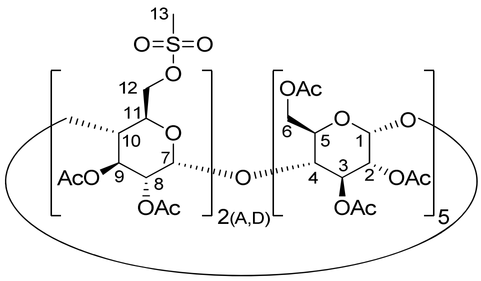

Under argon atmosphere **36** (547 mg, 0.42 mmol) was dissolved in pyridine (p.a., 8 mL) and acetic anhydride (4 mL) and stirred for 16 h at 60 °C. The reaction was quenched by addition of methanol (4 mL) and all volatile components were removed *in vacuo*. The residue was dissolved in DCM (100 mL) and washed with aqueous HCl (1 M, 100 mL), aqueous $NaHCO_3$ (sat., 100 mL) and brine (100 mL), dried over $MgSO_4$, filtered and evaporated. After drying under reduced pressure the product was obtained as light yellow solid.

Yield: 87% (768 mg, 0.37 mmol).

Empirical formula (MW in g/mol): $C_{82}H_{112}O_{58}S_2$ (2089.86).

^{1}H NMR (600 MHz, $CDCl_3$) δ 5.35 - 5.22 (m, 7H, H-3/9), 5.11 (d, $J = 3.9$ Hz, 1H, H-1/7), 5.10 - 5.06 (m, 6H, H-1/7), 4.84 - 4.71 (m, 7H, H-2/8), 4.68 - 4.41 (m, 10H, H-6/12), 4.34 - 4.21 (m, 4H, H-6/12), 4.21 - 4.15 (m, 2H, H-5/11), 4.15 - 4.08 (m, 5H, H-5/11), 3.75 - 3.67 (m, 7H, H-4/10), 3.10 (s, 3H, H-13), 3.09 (s, 3H, H-13), 2.14 - 2.00 (m, 57H, CH_3, OAc).

^{13}C{^{1}H} NMR (151 MHz, $CDCl_3$) δ 170.81 - 170.50, 169.53 - 169.42 (C_q, OAc), 97.06, 96.88, 96.85, 96.75, 96.69, 96.58 (C-1/7), 76.90, 76.62, 76.59, 76.46, 76.37, 76.24

(C-4/10), 71.09, 71.04, 70.84, 70.77, 70.74, 70.71, 70.66, 70.61, 70.49, 70.37, 70.14, 70.05, 70.01, 69.94, 69.71, 69.66, 69.63, 69.61, 69.54, 69.53 (C-2/8, 3/9, 5/11), 68.25, 68.20, 62.72, 62.71, 62.67, 62.58 (C-6/12), 37.56, 37.52 (C-13), 20.95 - 20.81 (CH_3, OAc).

MS-ESI-EM (+) (m/z): Calculated for $[C_{82}H_{112}O_{58}S_2Na_2]^{2+}$: 1067.25202; found: 1067.25296. Calculated for $[C_{82}H_{112}O_{58}S_2Na]^{+}$: 2111.51481; found: 2111.51438.

A,D-Di-(2,3-di-*O*-acetyl-6-deoxy-6-azido)-tetrakis-(2,3,6-tri-*O*-acetyl)-α-cyclodextrin (**39**)

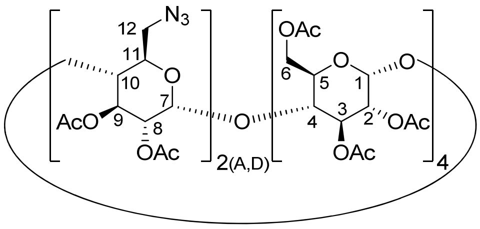

37 (900 mg, 0.50 mmol) and NaN_3 (650 mg, 9.99 mmol) were dissolved in DMF (SPPS grade, 12 mL) and stirred for 17 h at 80 °C. After removing all volatile components *in vacuo* the residue was taken up in DCM (75 mL) and dH_2O (75 mL). The organic phase was isolated and the aqueous phase was extracted with DCM (2 x 75 mL). The combined organic phases were washed with brine (100 mL), dried over $MgSO_4$, filtered and evaporated. The desired product was obtained after column chromatography (silica, DCM/MeOH 20:1) as white solid.

Yield: quant. (844 mg, 0.50 mmol).

Empirical formula (MW in g/mol): $C_{68}H_{90}N_6O_{44}$ (1695.46).

R_f-value: 0.30 (silica, DCM/MeOH 20:1).

^1H NMR (600 MHz, $CDCl_3$) δ 5.60 (dd, *J* = 10.5, 8.8 Hz, 2H, H-3/10), 5.52 (dd, *J* = 10.2, 8.4 Hz, 2H, H-3/10), 5.41 (dd, *J* = 10.1, 8.5 Hz, 2H, H-3/10), 5.12 (d, *J* = 3.6 Hz, 2H, H-1/7), 5.08 (d, *J* = 3.7 Hz, 2H, H-1/7), 4.94 (d, *J* = 3.5 Hz, 2H, H-1/7), 4.80 - 4.73 (m, 6H,

H-2/8), 4.43 - 4.32 (m, 8H, H-6), 4.18 - 4.07 (m, 6H, H-5/11), 3.85 - 3.70 (m, 10H, H-4/10, 12), 2.20 - 2.01 (m, 48H, C*H₃*, OAc).

$^{13}C\{^{1}H\}$ NMR (151 MHz, $CDCl_3$) δ 170.82 - 170.42, 169.36 - 169.22 (C_q, OAc), 96.87, 96.76, 96.62 (C-1/7), 78.45, 76.95, 76.78 (C-4/10), 71.47, 71.40, 71.34, 71.09, 70.90, 70.69, 69.97, 69.38, 69.37 (C-2/8, 3/9, 5/11), 63.25, 63.20 (C-6), 51.58 (C-12), 20.99 - 20.86 (CH_3, OAc).

MALDI-MS (+; DHB; EtOAc) (m/z): Calculated for $[C_{68}H_{90}N_6O_{44}Na]^+$: 1717.5; found: 1717.5.

A,D-Di-(2,3-di-*O*-acetyl-6-deoxy-6-azido)-pentakis-(2,3,6-tri-*O*-acetyl)-β-cyclodextrin (**40**)

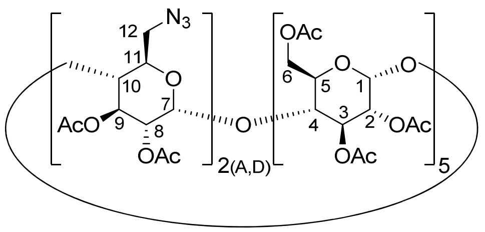

38 (546 mg, 0.27 mmol) and NaN_3 (351 mg, 5.40 mmol) were dissolved in DMF (SPPS grade, 8 mL) and stirred for 17 h at 80 °C. After removing all volatile components *in vacuo* the residue was taken up in DCM (75 mL) and dH_2O (75 mL). The organic phase was isolated and the aqueous phase was extracted with DCM (2 x 75 mL). The combined organic phases were washed with brine (100 mL), dried over $MgSO_4$, filtered and evaporated. The desired product was obtained after column chromatography (silica, DCM/MeOH 20:1) as white solid.

Yield: 90% (483 mg, 0.24 mmol).

Empirical formula (MW in g/mol): $C_{80}H_{106}N_6O_{52}$ (1983.71).

R_f-value: 0.42 (silica, DCM/MeOH 20:1).

^1H NMR (600 MHz, $CDCl_3$) δ 5.36 - 5.17 (m, 7H, H-3/9), 5.15 - 5.08 (m, 4H, H-1/7), 5.07 (d, *J* = 3.8 Hz, 1H, H-1/7), 5.04 - 4.99 (m, 2H, H-1/7), 4.85 - 4.74 (m, 7H, H-2/8), 4.63 - 4.50 (m, 4H, H-6), 4.30 - 4.14 (m, 6H, H-6), 4.14 - 4.01 (m, 7H, H-5/11), 3.80 - 3.64 (m, 11H, H-4/10, 12), 2.16 - 1.98 (m, 57H, C*H₃*, OAc).

$^{13}C\{^1H\}$ NMR (151 MHz, $CDCl_3$) δ 171.27 - 170.40, 169.59 - 169.50 (C_q, OAc), 97.25, 97.03, 97.01, 96.93, 96.86, 96.55 (C-1/7), 77.73, 76.89, 76.83, 76.75, 76.66, 76.37 (C-4/10), 71.42, 71.35, 71.27, 71.20, 70.93, 70.78, 70.60, 70.40, 70.35, 70.24, 69.80, 69.76, 69.71, 69.69, 69.48 (C-2/8, 3/9, 5/11), 62.85, 62.75, 62.60 (C-6), 50.95, 50.82 (C-12), 20.99 - 20.85 (CH_3, OAc).

MS-ESI-EM (+) (m/z): Calculated for $[C_{80}H_{106}N_6O_{52}Na]^+$: 2005.57258; found: 2005.57281.

A,D-Di-(2,3-di-*O*-acetyl-6-deoxy-6-amino)-tetrakis-(2,3,6-tri-*O*-acetyl)-α-cyclodextrin (**41**)

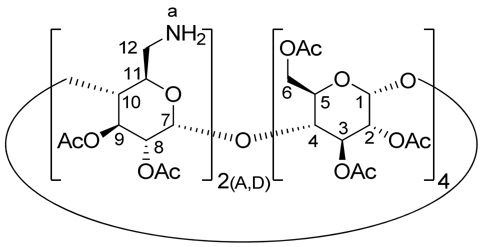

39 (844 mg, 0.50 mmol) was dissolved in DCM/methanol (50%/50%, v/v, 20 mL). After bubbling argon through the solution for 15 min Pd/C (10% Pd, 211 mg) was added and stirred under H_2 atmosphere for 5 d at room temperature. The catalyst was removed by filtration through a pad of *Celite*® and after evaporation the pure product was obtained as white solid.

Yield: 89% (732 mg, 0.45 mmol).

Empirical formula (MW in g/mol): $C_{68}H_{64}N_2O_{44}$ (1643.46).

^{1}H NMR (600 MHz, DMSO-d_6) δ 5.62 - 5.55 (m, 1H, H-1/7), 5.49 - 5.28 (m, 6H, H-3/9), 5.13 - 5.02 (m, 5H, H-1/7), 4.91 - 4.65 (m, 6H, H-2/8), 4.53 - 4.06 (m, 17H, 1xH-4/10, H-5/11, 6), 4.02 - 3.88 (m, 5H, H-4/10), 3.66 - 3.60 (m, 1H, H-12), 3.06 - 2.98 (m, 1H, H-12'), 2.13 - 1.92 (m, 48H, CH_3, OAc).

$^{13}C\{^{1}H\}$ NMR (151 MHz, DMSO-d_6) δ 170.44 - 169.70, 169.22 - 168.99 (C_q, OAc), 97.34, 97.27, 95.93 (C-1/7), 77.63, 76.74, 76.15 (C-4/10), 71.16, 70.61, 70.29, 70.17, 69.37, 69.24, 69.11, 68.91, 68.84, 68.47 (C-2/8, 3/9, 5/11), 62.72, 62.46 (C-6), 38.06 (C-12), 20.73 - 20.43 (CH_3, OAc).

MS-ESI-EM (+) (m/z): Calculated for $[C_{68}H_{64}N_2O_{44}H_2]^{2+}$: 822.26625; found: 822.26676. Calculated for $[C_{68}H_{64}N_2O_{44}H]^{+}$: 1643.52522; found: 1643.52643.

A,D-Di-(2,3-di-*O*-acetyl-6-deoxy-6-amino)-pentakis-(2,3,6-tri-*O*-acetyl)-β-cyclodextrin (**42**)

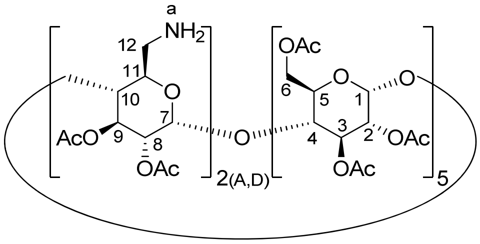

40 (457 mg, 0.23 mmol) was dissolved in DCM/methanol (50%/50%, v/v, 16 mL). After bubbling argon through the solution for 15 min Pd/C (10% Pd, 140 mg) was added and stirred under H_2 atmosphere for 3 d at room temperature. The catalyst was removed by filtration through a pad of *Celite*® and after evaporation the pure product was obtained as white solid.

Yield: 94% (419 mg, 0.22 mmol).

Empirical formula (MW in g/mol): $C_{80}H_{110}N_2O_{52}$ (1931.71).

^{1}H NMR (600 MHz, DMSO-d_6) δ 5.39 - 5.32 (m, 1H, H-1/7), 5.33 - 5.27 (m, 1H, H-1/7), 5.27 - 5.14 (m, 7H, H-3/9), 5.14 - 5.01 (m, 5H, H-1/7), 4.82 - 4.65 (m, 7H, H-2/8), 4.52 - 3.98 (m, 21H, 2xH-4/10, H-5/11, 6), 3.93 - 3.80 (m, 5H, H-4/11), 3.37 - 3.07 (m, 4H, H-12).

$^{13}C\{^{1}H\}$ NMR (151 MHz, DMSO-d_6) δ 170.24 - 169.81, 169.33 - 169.20 (C_q, OAc), 97.03, 96.73, 96.38, 96.20, 96.09, 96.06, 95.86 (C-1/7), 76.96, 76.67, 76.53, 76.46, 76.21, 76.06 (C-4/10), 70.20, 70.18, 70.09, 70.08, 69.98, 69.97, 69.96, 69.83, 69.65, 69.50, 69.45, 69.31, 69.20, 69.14, 69.13, 68.35, 68.11 (C-2/8, 3/9, 5/11), 62.68, 62.55, 62.53, 62.46, 62.27 (C-6), 38.77, 38.65 (C-12).

MS-ESI-EM (+) (m/z): Calculated for $[C_{80}H_{110}N_2O_{52}H_2]^{2+}$: 966.30851; found: 966.30924. Calculated for $[C_{80}H_{110}N_2O_{52}H]^{+}$: 1931.60974; found: 1931.61090.

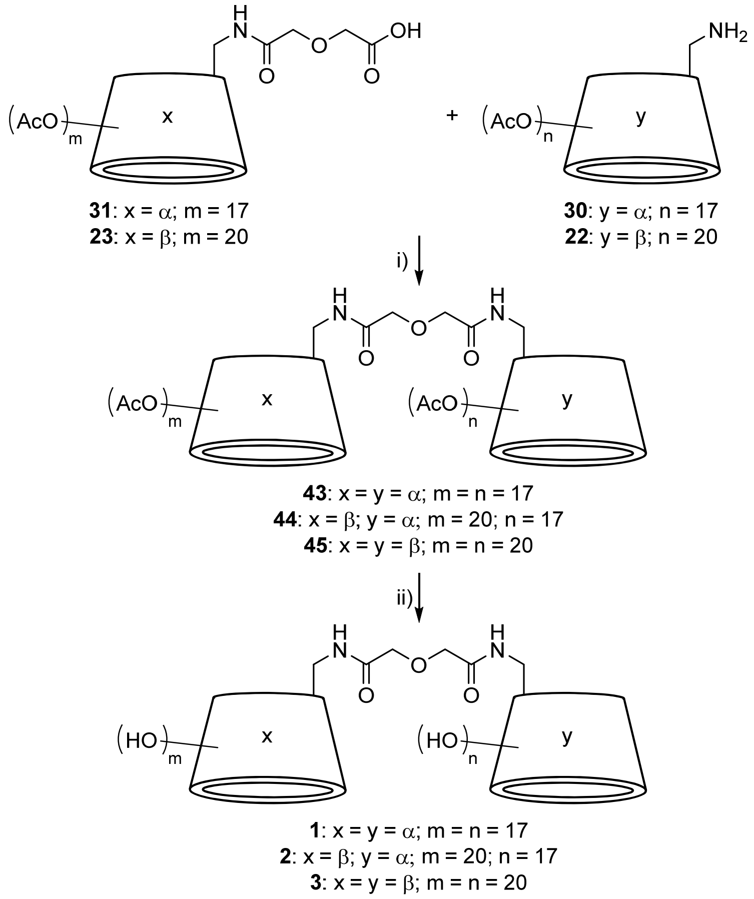

Figure S4: Synthesis of **1**, **2** and **3**: i) for **43**: **30**, **31**, Pybop®, DIPEA, DCM, 3 d, r.t., 96%; for **44**: **23**, **30**, Pybop®, DIPEA, DCM, 4 d, r.t., 66%; for **45**: **22**, **23**, Pybop®, DIPEA, DCM, 4 d, r.t., 80%; ii) **43**/**44**/**45**, NaOMe, MeOH, 24 h, r.t., quant. (**1**)/quant. (**2**)/quant. (**3**).

Because of their complex structure the following molecules are shown only schematically. The assignment of the NMR signals is based on the following numbering:

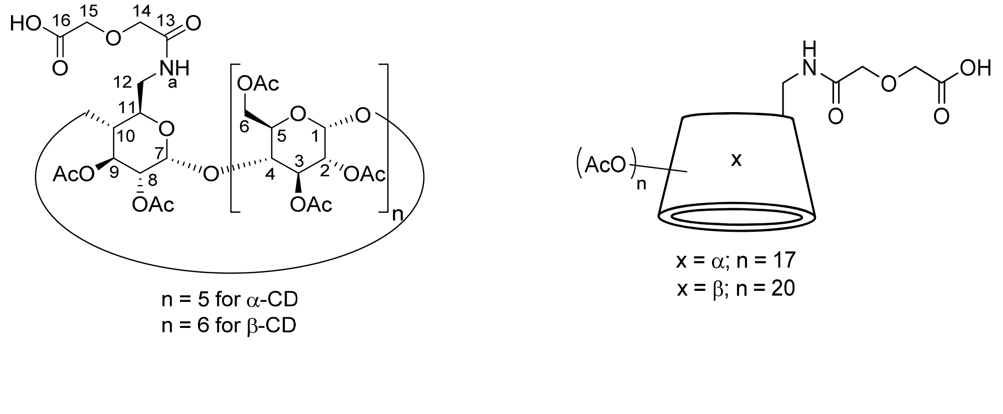

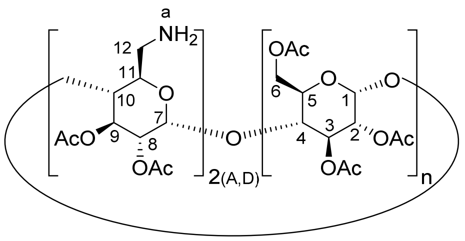

n = 4 for α-CD
n = 5 for β-CD

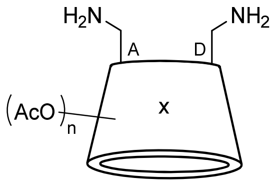

x = α; n = 16
x = β; n = 19

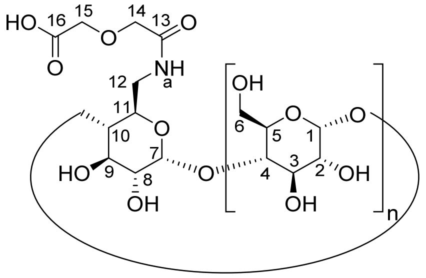

n = 5 for α-CD
n = 6 for β-CD

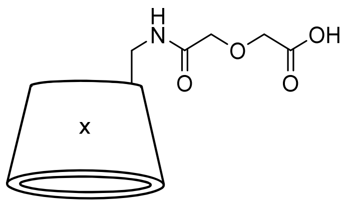

x = α; n = 17
x = β; n = 20

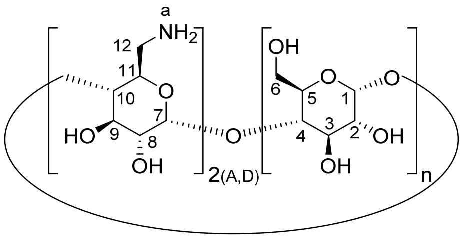

n = 4 for α-CD
n = 5 for β-CD

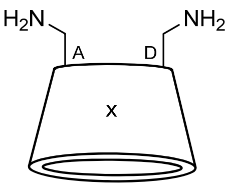

x = α; n = 16
x = β; n = 19

Peracetylated cyclodextrin sequence *α*-*α*-$(OAc)_{34}$ (**43**)

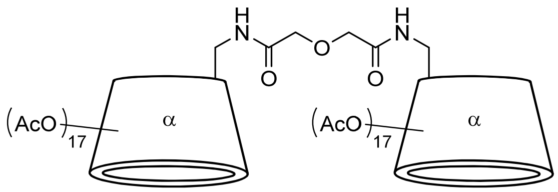

Under argon atmosphere **30** (48.1 mg, 28.5 µmol) was added to a solution of **31** (57.3 mg, 31.1 µmol), Pybop® (17 mg, 33 µmol) and DIPEA (10 µL, 57 µmol) in DCM (p.a., 1 mL). After stirring for 3 d at room temperature the solution was diluted with DCM (75 mL) and the organic phase was washed with aqueous $KHSO_4$ (1 M, 75 mL), aqueous $NaHCO_3$ (sat., 75 mL) and brine (75 mL), dried over $MgSO_4$, filtered and evaporated. The crude product was

purified by column chromatography (silica, DCM/MeOH 20:1) and size exclusion chromatography (*Sephadex*® *LH20*, MeOH (p.a.)) to obtain the pure product as light yellow solid.

Yield: 96% (104 mg, 30.0 µmol).

Empirical formula (MW in g/mol): $C_{144}H_{192}N_2O_{95}$ (3471.02).

R_f-value: 0.30 (silica, DCM/MeOH 20:1).

^{1}H NMR (600 MHz, $CDCl_3$) δ 7.12 - 7.05 (m, 2H, H-a), 5.61 - 5.32 (m, 12H, H-3/9), 5.28 (d, J = 3.8 Hz, 2H, H-1/7), 5.11 - 4.86 (m, 10H, H-1/7), 4.85 - 4.60 (m, 12H, H-2/8), 4.49 - 4.24 (m, 20H, H-6, 14), 4.22 - 4.02 (m, 12H, H-5/11), 3.88 - 3.65 (m, 14H, H-4/10, 12), 3.62 - 3.53 (m, 2H, H-12'), 2.22 - 1.88 (m, 102H, CH_3, OAc).

^{13}C{^{1}H} NMR (151 MHz, $CDCl_3$) δ 171.06 - 170.37, 169.50 - 169.17 (C-13, C_q, OAc), 96.93 - 96.10 (C-1/7), 78.91 - 76.30 (C-4/10), 71.74 - 68.85 (C-2/8, 3/9, 5/11, 14), 63.35 - 63.05 (C-6), 40.06 (C-12), 20.99 - 20.78 (CH_3, OAc).

MALDI-MS (+; DHB; EtOAc) (m/z): Calculated for $[C_{144}H_{192}N_2O_{95}Na]^+$: 3493.02; found: 3493.05.

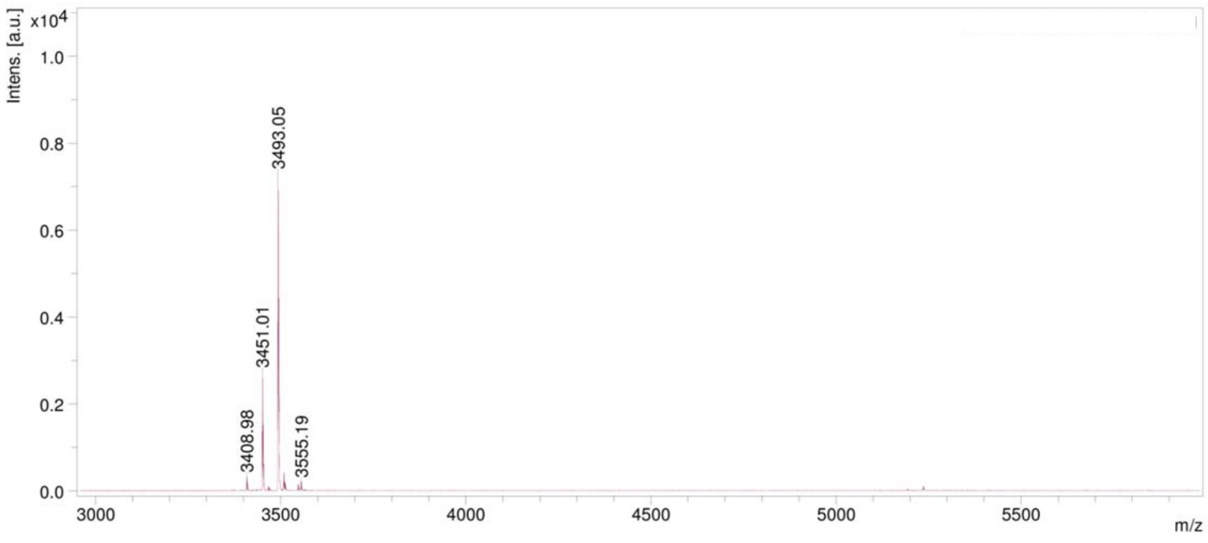

Figure S5: MALDI mass spectra of **43** (matrix: DHB, solvent for preparation: EtOAc).

Peracetylated cyclodextrin sequence *α*-*β*-$(OAc)_{37}$ (**44**)

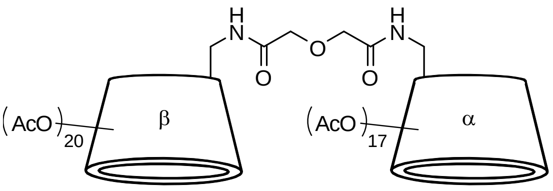

Under argon atmosphere **30** (55.9 mg, 33.2 µmol) was added to a solution of **23** (104 mg, 49.8 µmol), Pybop (26 mg, 50 µmol) and DIPEA (10 µL, 57 µmol) in DCM (p.a., 1 mL). After stirring for 4 d at room temperature the solution was diluted with DCM (75 mL) and the organic phase was washed with aqueous $KHSO_4$ (1 M, 75 mL), aqueous $NaHCO_3$ (sat., 75 mL) and brine (75 mL), dried over $MgSO_4$, filtered and evaporated. The crude product was purified by column chromatography (silica, DCM/MeOH 20:1) and size exclusion chromatography (*Sephadex® LH20*, MeOH (p.a.)) to obtain the pure product as white solid.

Yield: 66% (82.4 mg, 21.8 µmol).

Empirical formula (MW in g/mol): $C_{156}H_{208}N_2O_{103}$ (3759.27).

R_f-value: 0.16 (silica, DCM/MeOH 20:1).

^1H NMR (600 MHz, $CDCl_3$) δ 7.23 - 7.18 (m, 1H, H-a), 7.16 (t, J = 6.5 Hz, 1H, H-a), 5.61 - 5.38 (m, 6H, 1xH-1/7, 5xH-3/9), 5.33 - 5.20 (m, 7H, H-3/9), 5.17 (d, J = 3.9 Hz, 1H, H-1/7), 5.11 - 4.94 (m, 12H, 11xH-1/7, 1xH-3/9), 4.85 - 4.68 (m, 13H, H-2/8), 4.61 - 3.94 (m, 40H, H-5/11, 6, 14, 1xH-12), 3.87 - 3.59 (m, 15H, 13xH-4/10, 2xH-12), 3.51 - 3.42 (m, 2H, 1xH-4/10, 1xH-12), 2.18 - 1.93 (m, 111H, CH_3, OAc).

$^{13}C\{^1H\}$ NMR (151 MHz, $CDCl_3$) δ 171.34 - 170.30, 169.64 - 169.13 (C-13, C_q, OAc), 97.62 - 96.39 (C-1/7), 78.55 - 76.51 (C-4/10), 71.74 - 68.95 (C-2/8, 3/9, 5/11, 14), 63.29 - 62.46 (C-6), 40.21, 39.89 (C-12), 21.03 - 20.77 (CH_3, OAc).

MALDI-MS (+; DHB; EtOAc) (m/z): Calculated for $[C_{156}H_{208}N_2O_{103}Na]^+$: 3781.10; found: 3782.23.

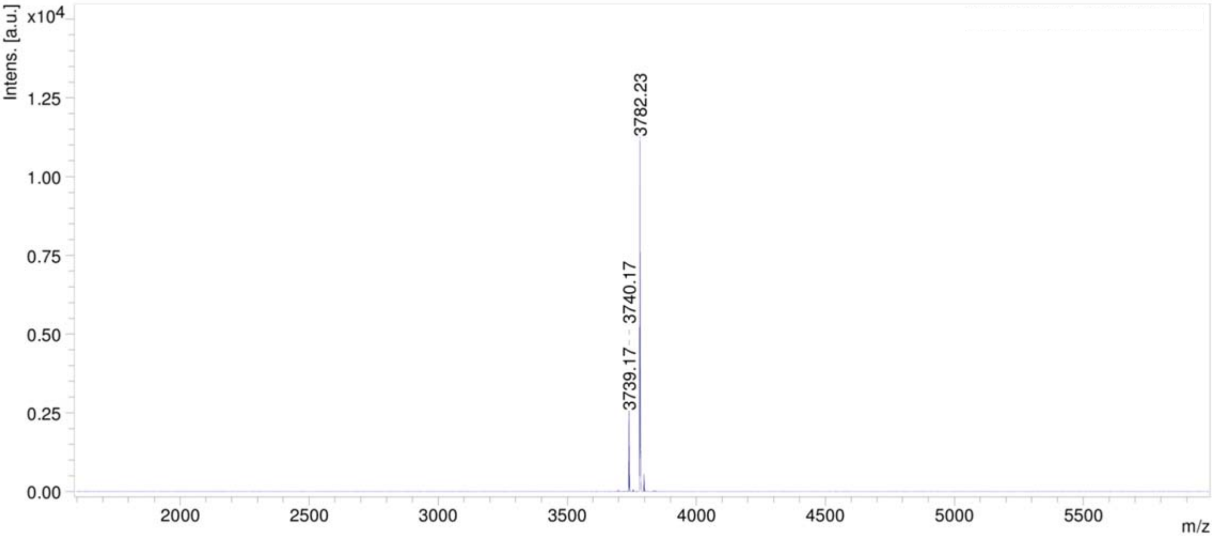

Figure S6: MALDI mass spectra of **44** (matrix: DHB, solvent for preparation: EtOAc).

Peracetylated cyclodextrin sequence *β*-*β*-$(OAc)_{40}$ (**45**)

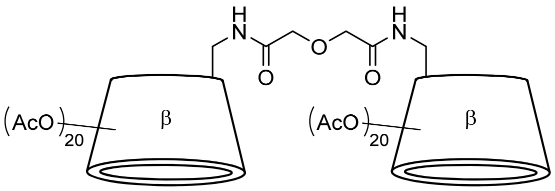

Under argon atmosphere **22** (102 mg, 51.7 µmol) was added to a solution of **23** (167 mg, 79.9 µmol), Pybop® (42 mg, 80 µmol) and DIPEA (14 µL, 80 µmol) in DCM (p.a., 2 mL). After stirring for 4 d at room temperature the solution was diluted with DCM (50 mL) and the organic phase was washed with aqueous $KHSO_4$ (1 M, 50 mL), aqueous $NaHCO_3$ (sat., 50 mL) and brine (50 mL), dried over $MgSO_4$, filtered and evaporated. The crude product was purified by column chromatography (silica, DCM/MeOH 20:1) and size exclusion chromatography (*Sephadex® LH20*, MeOH (p.a.)) to obtain the pure product as white solid.

Yield: 80% (168 mg, 41.5 µmol).

Empirical formula (MW in g/mol): $C_{168}H_{224}N_2O_{111}$ (4047.52).

R_f-value: 0.28 (silica, DCM/MeOH 20:1).

^{1}H NMR (600 MHz, $CDCl_3$) δ 7.19 (s, 2H, H-a), 5.37 - 5.17 (m, 14H, 2xH-1/7, 12xH-3/9), 5.17 - 5.10 (m, 2H, H-3/9), 5.10 - 4.94 (m, 12H, H-1/7), 4.82 - 4.65 (m, 14H, H-2/8), 4.56 - 3.89 (m, 42H, H-5/11, 6, 14), 3.78 - 3.46 (m, 18H, H-4/11, 12), 2.15 - 1.91 (m, 120H, C*H*$_3$, OAc).

^{13}C{^{1}H} NMR (151 MHz, $CDCl_3$) δ 170.97 - 170.40, 169.58 - 169.27 (C-13, C_q, OAc), 97.40 - 96.47 (C-1/7), 77.96 - 76.30 (C-4/10), 71.10 - 69.21 (C-2/8, 3/9, 5/11, 14), 62.94 - 62.49 (C-6), 39.73 (C-12), 20.88 - 20.71 (CH_3, OAc).

MALDI-MS (+, DHB, EtOAc) (m/z): Calculated for $[C_{168}H_{224}N_2O_{111}Na]^+$: 4069.19; found: 4070.30.

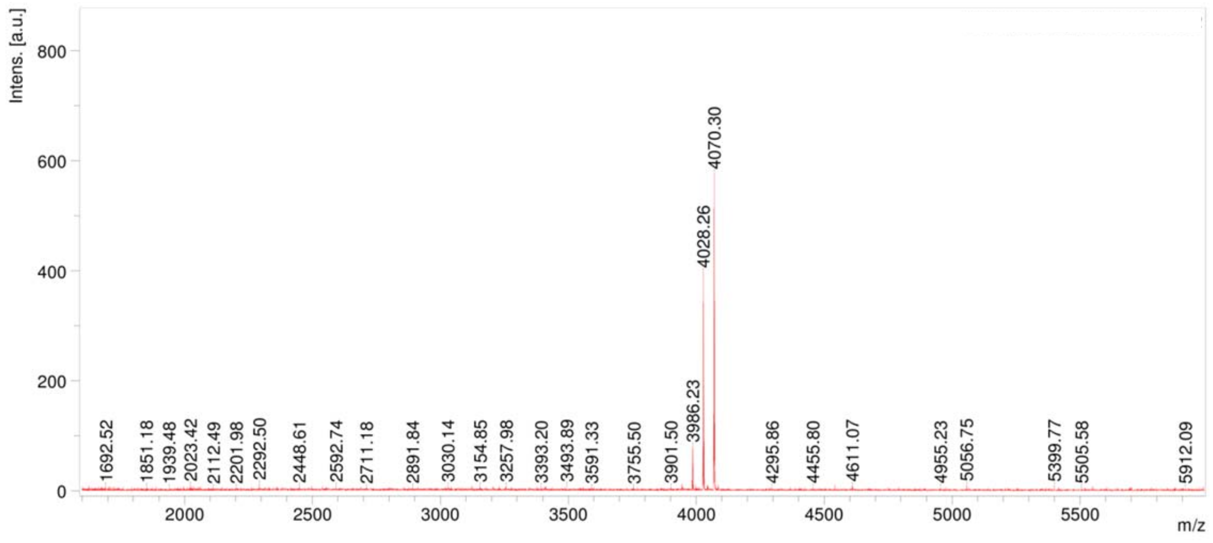

Figure S7: MALDI mass spectra of **45** (matrix: DHB, solvent for preparation: EtOAc).

Cyclodextrin sequence *α*-*α*-$(OH)_{34}$ (**1**)

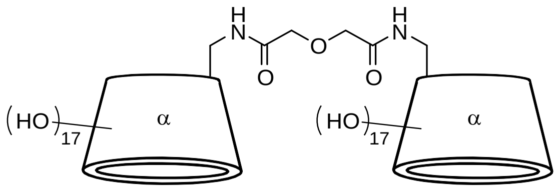

43 (104 mg, 30.0 µmol) was dissolved in MeOH (p.a., 5 mL) and sodium methoxide (cat.) was added. After stirring for 24 h at room temperature the solution was neutralized by addition of ion exchange resin (*Dowex® HCR-W2 hydrogen form*), stirred for 1 h, filtered and evaporated. After freeze drying the product was obtained as white solid.

Yield: quant. (61.3 mg, 30.0 µmol).

Empirical formula (MW in g/mol): $C_{76}H_{124}N_2O_{61}$ (2041.77).

^{1}H NMR (600 MHz, DMSO-d_6) δ 7.78 - 7.73 (d, *J* = 5.9 Hz, 2H, H-a), 5.45 (brs, 24H, 2/8-, 3/9-O*H*), 4.90 - 4.75 (m, 12H, H-1/7), 3.96 - 3.52 (m, 50H, H-3/9, 5/11, 6, 12, 14), 3.43 - 3.23 (m, 28H, H-2/8, 4/10, 12’).

$^{13}C\{^1H\}$ NMR (151 MHz, DMSO-d_6) δ 169.00 (C-13), 102.00 - 101.87 (C-1/7), 82.10 - 82.02 (C-4/10), 73.25 - 71.89 (C-2/8, 3/9, 5/11, 14), 60.02 - 59.93 (C-6), 39.62 (C-12).

MALDI-MS (+; DHB; H_2O/ACN) (m/z): Calculated for $[C_{76}H_{124}N_2O_{61}Na]^+$: 2063.66; found: 2063.65.

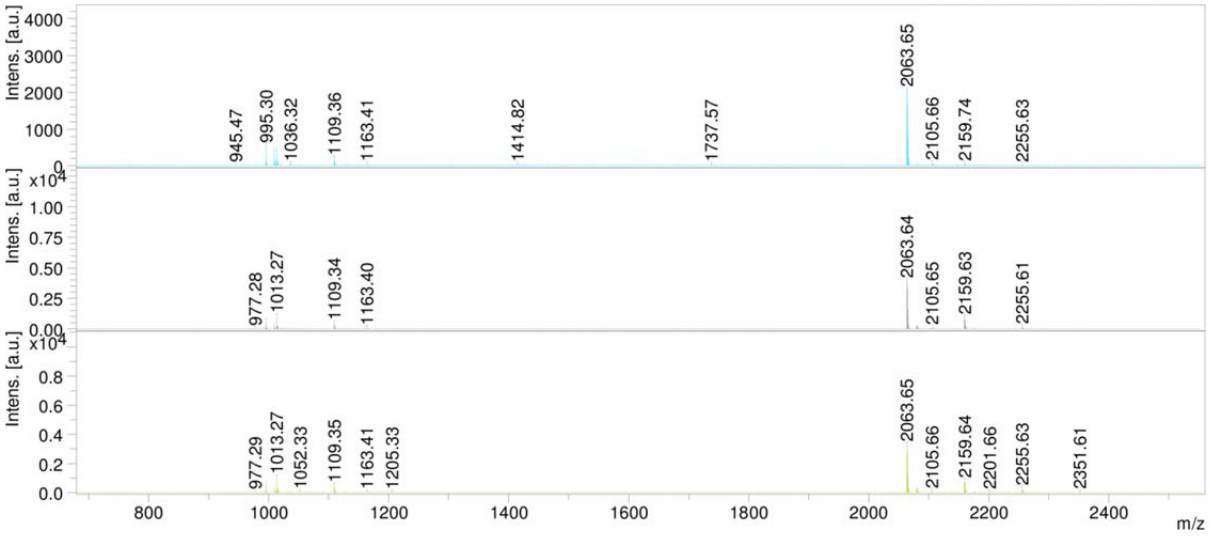

Figure S8: MALDI mass spectra of **1** (matrix: DHB, solvent for preparation: H_2O/ACN).

Cyclodextrin sequence *α*-*β*-$(OH)_{37}$ (**2**)

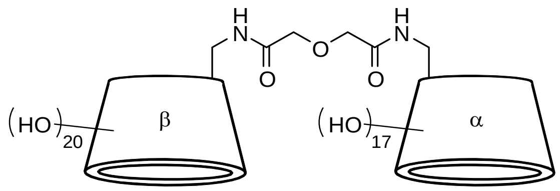

44 (82.4 mg, 21.8 µmol) was dissolved in MeOH (p.a., 5 mL) and sodium methoxide (cat.) was added. After stirring for 24 h at room temperature the solution was neutralized by addition of ion exchange resin (*Dowex® HCR-W2 hydrogen form*), stirred for 1 h, filtered and evaporated. After freeze drying the product was obtained as white solid.

Yield: quant. (47.9 mg, 21.7 µmol).

Empirical formula (MW in g/mol): $C_{82}H_{134}N_2O_{66}$ (2203.92).

^{1}H NMR (600 MHz, DMSO-d_6) δ 7.81 - 7.75 (m, 2H, H-a), 5.53 (s, 26H, 2/8-, 3/9-O*H*), 4.95 - 4.72 (m, 13H, H-1/7), 4.04 - 3.47 (m, 54H, H-3/9, 5/11, 6, 12, 14), 3.47 - 3.00 (m, 28H, H-2/8, 4/10, 12’).

^{13}C{^{1}H} NMR (151 MHz, DMSO-d_6) δ 168.88 (C-13), 101.98 - 101.62 (C-1/7), 82.10 - 81.35 (C-4/10), 73.25 - 69.69 (C-2/8, 3/9, 5/11, 14), 60.02 - 59.84 (C-6), 39.65 (C-12).

MALDI-MS (+; DHB; H_2O/ACN) (m/z): Calculated for $[C_{82}H_{134}N_2O_{66}Na]^+$: 2225.71; found: 2225.71.

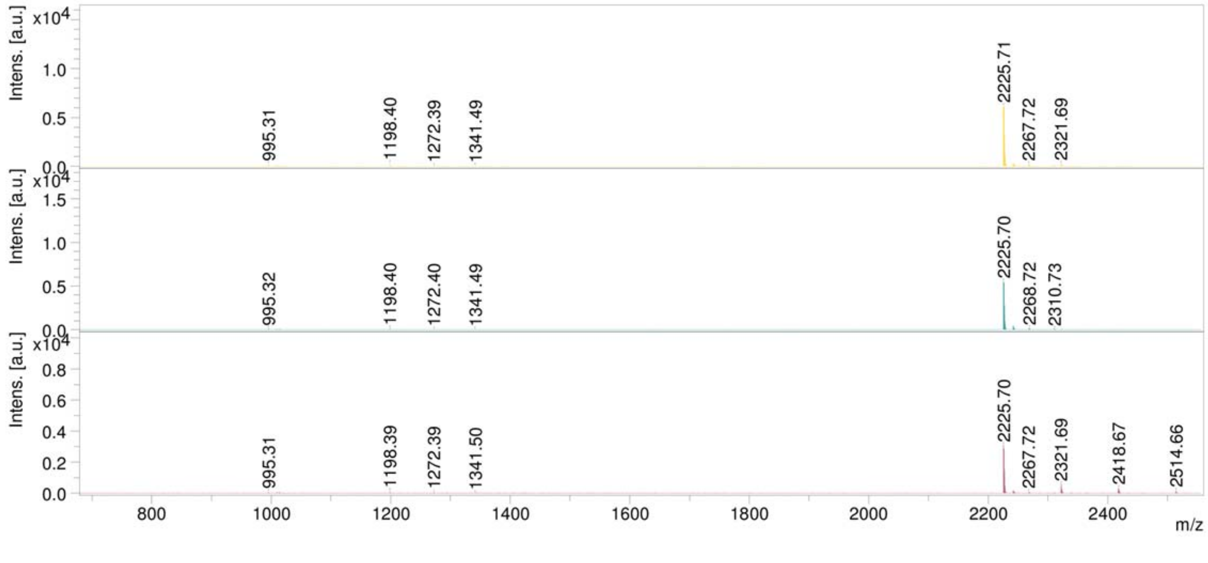

Figure S9: MALDI mass spectra of **2** (matrix: DHB, solvent for preparation: H_2O/ACN).

Cyclodextrin sequence *β*-*β*-$(OH)_{34}$ (**3**)

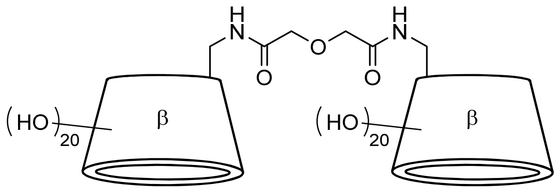

45 (168 mg, 41.5 μmol) was dissolved in MeOH (p.a., 5 mL) and sodium methoxide (cat.) was added. After stirring for 24 h at room temperature the solution was neutralized by addition of ion exchange resin (*Dowex® HCR-W2 hydrogen form*), stirred for 1 h, filtered and evaporated. After freeze drying the product was obtained as white solid.

Yield: quant. (98.4 mg, 41.6 μmol).

Empirical formula (MW in g/mol): $C_{88}H_{144}N_2O_{71}$ (2366.06).

^{1}H NMR (600 MHz, DMSO-d_6) δ 7.79 (t, J = 5.8 Hz, 2H, H-a), 5.69 (brs, 28H, 2/8-, 3/9-O*H*), 5.10 - 4.78 (m, 14H, H-1/7), 4.03 - 3.49 (m, 58H, H-3/9, 5/11, 6, 12, 14), 3.43 - 3.09 (m, 30H, H-2/8, 4/10, 12').

^{13}C{^{1}H} NMR (151 MHz, DMSO-d_6) δ 168.84 (C-13), 102.23 - 101.67 (C-1/7), 83.88 - 81.30 (C-4/11), 73.15 - 69.76 (C-2/8, 3/9, 5/11, 14), 60.02 - 59.75 (C-6), 39.69 (C-12).

MALDI-MS (+; DHB; H_2O/ACN) (m/z): Calculated for $[C_{88}H_{144}N_2O_{71}Na]^+$: 2387.76; found: 2388.77.

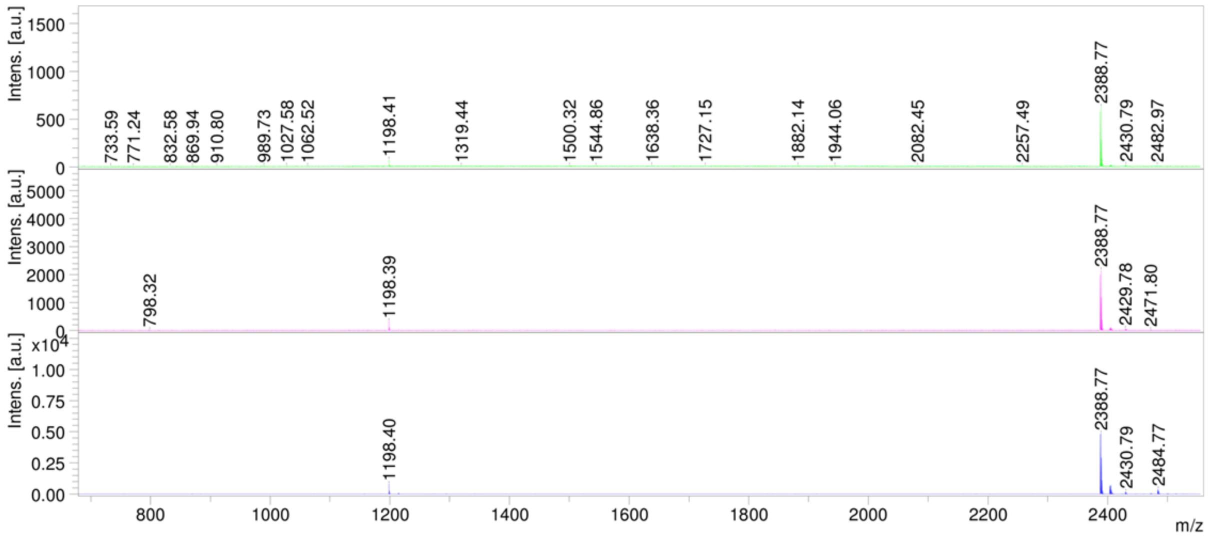

Figure S10: MALDI mass spectra of **3** (matrix: DHB, solvent for preparation: H_2O/ACN).

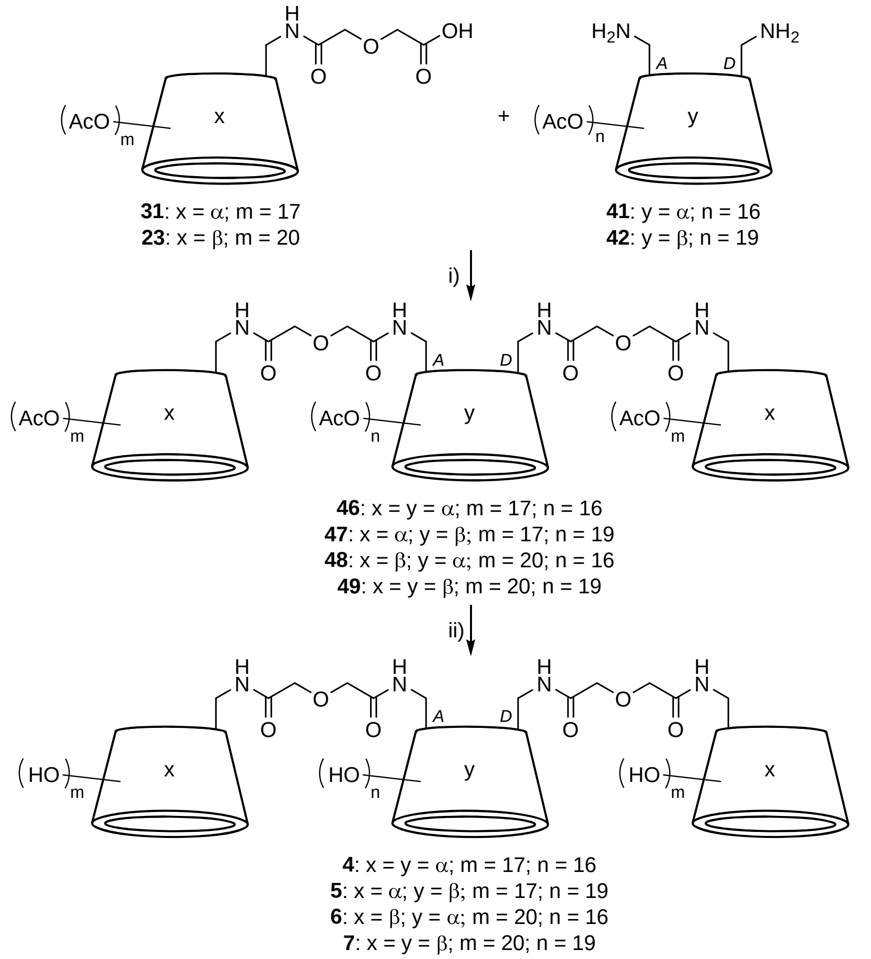

Figure S11: Synthesis of **4** - **7**: i) for **46**: **31**, **41**, Pybop®, DIPEA, DCM, 5 d, r.t., 29%; for **47**: **31**, **42**, Pybop®, DIPEA, DCM, 5 d, r.t., 26%; for **48**: **23**, **41**, Pybop®, DIPEA, DCM, 5 d, r.t., 29%; for **49**: **23**, **42**, Pybop®, DIPEA, DCM, 5 d, r.t., 31%; ii) **46**/**47**/**48**/**49**, NaOMe, MeOH, 36 h, r.t., quant. (**4**)/quant. (**5**)/quant. (**6**)/quant. (**7**).

Peracetylated cyclodextrin sequence *α-α-α*-(OAc)$_{50}$ (**46**)

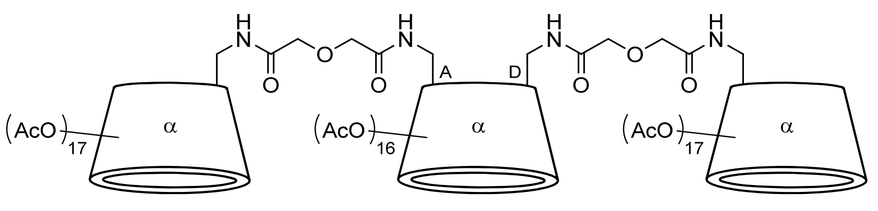

Under argon atmosphere **41** (65.5 mg, 39.9 µmol) was added to a solution of **31** (157 mg, 87.1 µmol), Pybop® (45.0 mg, 87.1 µmol) and DIPEA (20.0 µL, 115 µmol) in DCM (p.a., 1 mL). After stirring for 5 d at room temperature the solution was diluted with DCM (75 mL) and the organic phase was washed with aqueous $KHSO_4$ (1 M, 75 mL), aqueous $NaHCO_3$

(sat., 75 mL) and brine (75 mL), dried over $MgSO_4$, filtered and evaporated. The crude product was purified by size exclusion chromatography (*BioBeads*® *S-X1*, THF (p.a., distilled), pure fractions were identified by MALDI-MS) to obtain the pure product as white solid.

Yield: 29% (59.8 mg, 11.5 μmol).

Empirical formula (MW in g/mol): $C_{216}H_{288}N_4O_{142}$ (5212.54).

^{1}H NMR (600 MHz, $CDCl_3$) δ 7.10 - 7.04 (m, 2H, H-a), 7.04 - 6.99 (m, 2H, H-a), 5.66 - 5.39 (m, 18H, H-3/9), 5.39 - 5.35 (m, 2H, H-1/7), 5.30 (d, J = 3.8 Hz, 2H, H-1/7), 5.15 - 4.97 (m, 14H, H-1/7), 4.87 - 4.81 (m, 4H, H-2/8), 4.79 - 4.68 (m, 14H, H-2/8), 4.52 - 4.04 (m, 56H, H-5/11, 6, 14, 15, 2xH-12), 3.90 - 3.71 (m, 18H, 16xH-4/10, 2xH-12), 3.69 - 3.56 (m, 4H, 2xH-4/10, 2xH-12), 3.55 - 3.49 (m, 2H, H-12), 2.21 - 1.93 (m, 150H, CH_3, OAc).

^{13}C{^{1}H} NMR (151 MHz, $CDCl_3$) δ 171.16 - 170.24, 169.57 - 169.21 (C-13, 16, C_q, OAc), 97.12 - 96.19 (C-1/7), 77.77 - 76.45 (C-4/10), 71.84 - 68.65 (C-2/8, 3/9, 5/11, 14, 15), 63.46 - 63.11 (C-6), 40.18, 39.70 (C-12), 21.09 - 20.85 (CH_3, OAc).

MALDI-MS (+; DHB; EtOAc) (m/z): Calculated for $[C_{216}H_{288}N_4O_{142}Na]^+$: 5234.54; found: 5235.62.

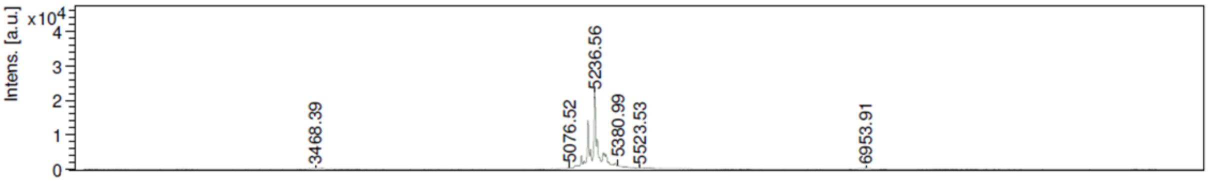

Figure S12: MALDI mass spectra of **46** (matrix: DHB, solvent for preparation: EtOAc).

Peracetylated cyclodextrin sequence *α-β-α*-(OAc)$_{53}$ (**47**)

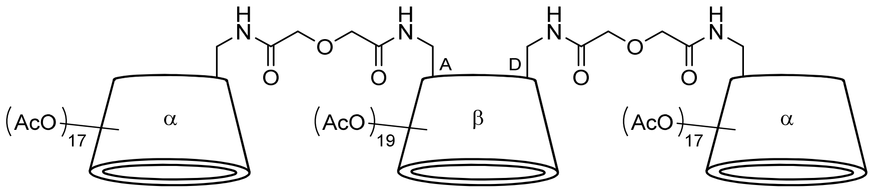

Under argon atmosphere **42** (80.0 mg, 41.4 μmol) was added to a solution of **23** (163 mg, 90.4 μmol), Pybop® (47.2 mg, 90.4 μmol) and DIPEA (20.0 μL, 115 μmol) in DCM (p.a., 1 mL). After stirring for 5 d at room temperature the solution was diluted with DCM (75 mL) and the organic phase was washed with aqueous $KHSO_4$ (1 M, 75 mL), aqueous $NaHCO_3$ (sat., 75 mL) and brine (75 mL), dried over $MgSO_4$, filtered and evaporated. The crude product was purified by size exclusion chromatography (*BioBeads® S-X1*, THF (p.a., distilled), pure fractions were identified by MALDI-MS) to obtain the pure product as white solid.

Yield: 26% (59.4 mg, 10.8 μmol).

Empirical formula (MW in g/mol): $C_{228}H_{304}N_4O_{150}$ (5500.79).

^{1}H NMR (600 MHz, $CDCl_3$) δ 7.24 - 7.05 (m, 4H, H-a), 5.67 - 5.35 (m, 12H, H-3/9), 5.35 - 5.15 (m, 7H, H-3/9), 5.14 - 4.96 (m, 19H, H-1/7), 4.89 - 4.67 (m, 19H, H-2/8), 4.63 - 3.95 (m, 51H, H-5/11, 6, 2xH-12), 3.88 - 3.43 (m, 25H, H-4/10, 6xH-12), 2.22 - 1.93 (m, 159H, C*H$_3$*, OAc).

^{13}C{^{1}H} NMR (151 MHz, $CDCl_3$) δ 171.06 - 170.47, 169.63 - 169.18 (C-13, 16, C$_q$, OAc), 97.06 - 96.40 (C-1/7), 78.81 - 76.53 (C-4/10), 71.71 - 68.95 (C-2/8, 3/9, 5/11, 14, 15), 63.36 - 62.69 (C-6), 39.96, 39.76 (C-12), 21.05 - 20.87 (CH_3, OAc).

MALDI-MS (+; DHB; EtOAc) (m/z): Calculated for $[C_{228}H_{304}N_4O_{150}Na]^+$: 5522.62; found: 5523.78.

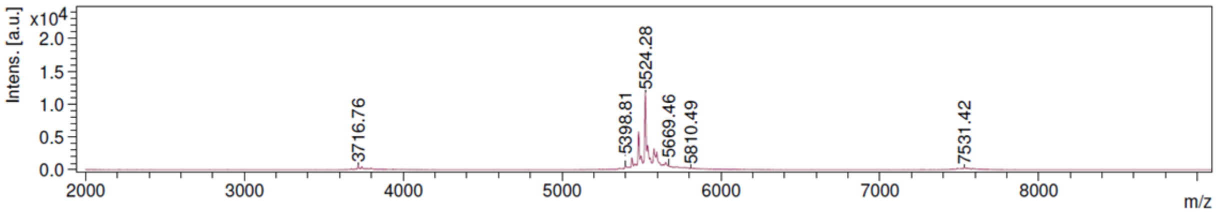

Figure S13: MALDI mass spectra of **47** (matrix: DHB, solvent for preparation: EtOAc).

Peracetylated cyclodextrin sequence *β-α-β*-$(OAc)_{56}$ (**48**)

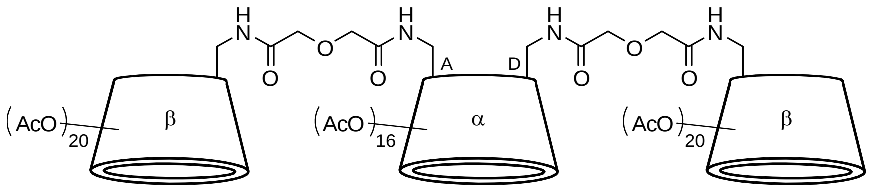

Under argon atmosphere **41** (103 mg, 62.7 µmol) was added to a solution of **23** (331 mg, 158 µmol), Pybop® (82.3 mg, 158 µmol) and DIPEA (28.0 µL, 158 µmol) in DCM (p.a., 1 mL). After stirring for 5 d at room temperature the solution was diluted with DCM (100 mL) and the organic phase was washed with aqueous $KHSO_4$ (1 M, 100 mL), aqueous $NaHCO_3$ (sat., 100 mL) and brine (100 mL), dried over $MgSO_4$, filtered and evaporated. The crude product was purified by size exclusion chromatography (*BioBeads*® *S-X1*, THF (p.a., distilled), pure fractions were identified by MALDI-MS) to obtain the pure product as white solid.

Yield: 29% (106 mg, 18.3 µmol).

Empirical formula (MW in g/mol): $C_{240}H_{320}N_4O_{158}$ (5789.04).

^{1}H NMR (600 MHz, $CDCl_3$) δ 7.32 (s, 2H, H-a), 7.19 (s, 2H, H-a), 5.58 - 5.51 (m, 4H, H-3/9), 5.47 (t, *J* = 9.4 Hz, 1H, H-3/9), 5.42 (t, *J* = 9.4 Hz, 1H, H-3/9), 5.33 - 5.21 (m, 12H, H-3/9), 5.16 (d, *J* = 4.0 Hz, 2H, H-1/7), 5.11 - 5.00 (m, 16H, 14xH-1/7, 2xH-3/9), 4.96 (d, *J* = 4.2 Hz, 2H, H-1/7), 4.88 - 4.64 (m, 20H, H-2/8), 4.63 - 3.94 (m, 61H,

H-5/11, 6, 14, 15, 1xH-12), 3.89 - 3.82 (m, 1H, H-12), 3.82 - 3.60 (m, 23H, 18xH-4/10, 5xH-12), 3.46 - 3.39 (m, 2H, H-4/10), 3.39 - 3.29 (m, 1H, H-12), 2.20 - 1.91 (m, 168H, CH_3, OAc).

$^{13}C\{^1H\}$ NMR (151 MHz, $CDCl_3$) δ 171.56 - 170.13, 169.81 - 169.03 (C-13, 16, C_q, OAc), 97.81 - 96.29 (C-1/7), 78.73 - 76.31 (C-4/10), 71.70 - 68.85 (C-2/8, 3/9, 5/11, 14, 15), 63.35 - 62.32 (C-6), 40.55, 39.74 (C-12), 21.01 - 20.71 (CH_3, OAc).

MALDI-MS (+; DHB; EtOAc) (m/z): Calculated for $[C_{240}H_{320}N_4O_{158}Na]^+$: 5810.71; found: 5810.75.

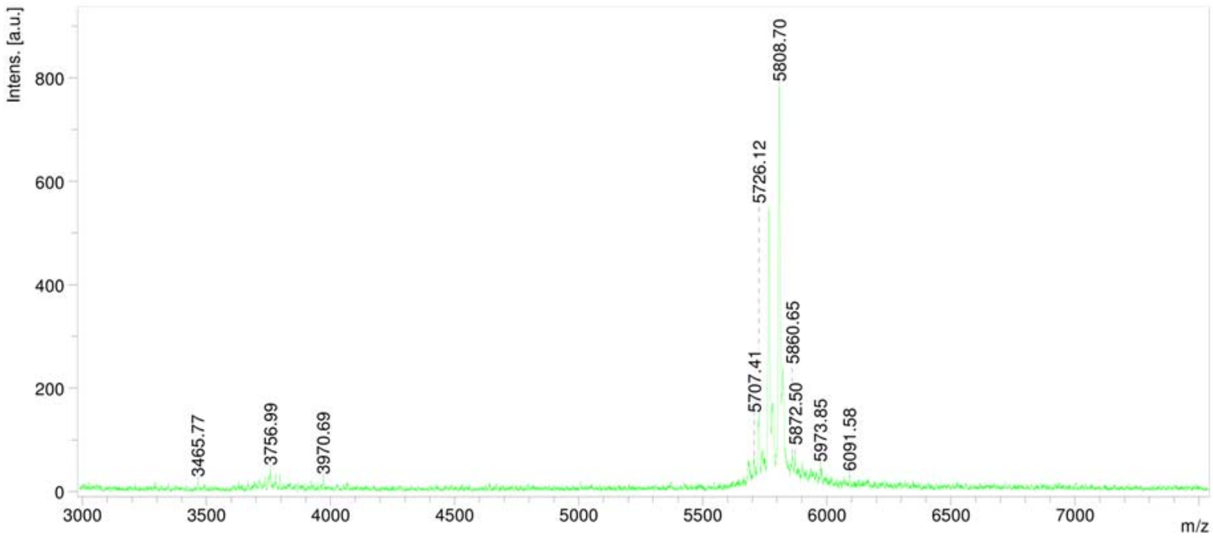

Figure S14: MALDI mass spectra of **48** (matrix: DHB, solvent for preparation: EtOAc).

Peracetylated cyclodextrin sequence *β-β-β*-(OAc)$_{59}$ (**49**)

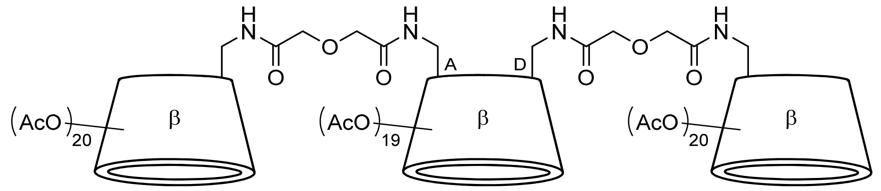

Under argon atmosphere **42** (101 mg, 52.3 μmol) was added to a solution of **23** (263 mg, 126 μmol), Pybop® (66.8 mg, 129 μmol) and DIPEA (22.0 μL, 129 μmol) in DCM (p.a., 1 mL). After stirring for 5 d at room temperature the solution was diluted with DCM (75 mL) and the organic phase was washed with aqueous $KHSO_4$ (1 M, 75 mL), aqueous $NaHCO_3$ (sat., 75 mL) and brine (75 mL), dried over $MgSO_4$, filtered and evaporated. The crude product was purified by size exclusion chromatography (*BioBeads® S-X1*, THF (p.a., distilled), pure fractions were identified by MALDI-MS) to obtain the pure product as white solid.

Yield: 31% (98.1 mg, 16.1 μmol).

Empirical formula (MW in g/mol): $C_{252}H_{336}N_4O_{166}$ (6077.29).

^{1}H NMR (600 MHz, $CDCl_3$) δ 7.25 - 7.11 (m, 4H, H-a), 5.39 - 5.10 (m, 24H, 3xH-1/7, 21xH-3/9), 5.10 - 4.95 (m, 18H, H-1/7), 4.86 - 4.68 (m, 21H, H-2/8), 4.60 - 3.87 (m, 64H, H-5/11, 6, 14, 15, 1xH-12), 3.80 - 3.38 (m, 28H, H-4/10, 7xH-12), 2.15 - 1.95 (m, 177H, CH_3, OAc).

^{13}C{^{1}H} NMR (151 MHz, $CDCl_3$) δ 171.20 - 170.45, 169.61 - 169.19 (C-13, 16, C_q, OAc), 97.61 - 96.44 (C-1/7), 78.03 - 76.39 (C-4/10), 71.23 - 69.16 (C-2/8, 3/9, 5/11, 14, 15), 63.11 - 62.46 (C-6), 40.08, 39.84, 39.80, 39.40 (C-12), 20.95 - 20.75 (CH_3, OAc).

MALDI-MS (+; DHB; EtOAc) (m/z): Calculated for $[C_{252}H_{336}N_4O_{166}Na]^+$: 6098.79; found: 6099.52.

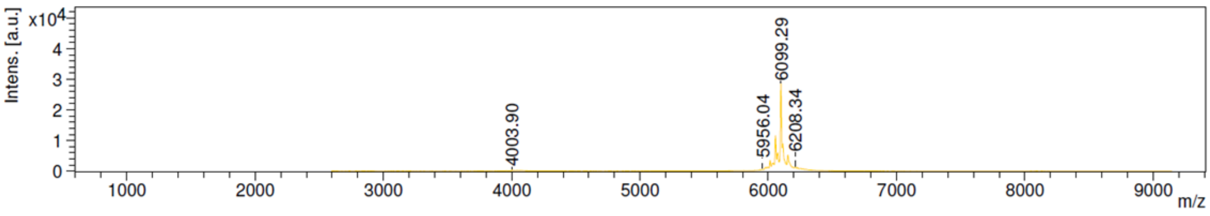

Figure S15: MALDI mass spectra of **49** (matrix: DHB, solvent for preparation: EtOAc).

Cyclodextrin sequence *α*-*α*-*α*-$(OH)_{50}$ (**4**)

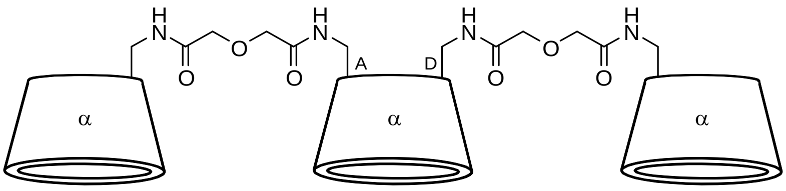

46 (56.1 mg, 10.8 µmol) was dissolved in MeOH (p.a., 5 mL) and sodium methoxide (cat.) was added. After stirring for 36 h at room temperature the solution was neutralized by addition of ion exchange resin (*Dowex® HCR-W2 hydrogen form*), stirred for 1 h, filtered and evaporated. After freeze drying the product was obtained as white solid.

Yield: quant. (33.6 mg, 10.8 µmol).

Empirical formula (MW in g/mol): $C_{116}H_{188}N_4O_{92}$ (3110.71).

^{1}H NMR (600 MHz, DMSO-d_6) δ 5.54 (s, 36H, 2/8, 3/9-O*H*), 4.95 - 4.72 (m, 18H, H-1/7), 4.55 (s, 14H, 6-O*H*), 4.01 - 3.52 (m, 72H, H-3/9, 5/11, 6, 12, 14, 15), 3.51 - 3.12 (m, 40H, H-2/8, 4/10, 12', overlapping with HDO from solvent).

^{13}C{^{1}H} NMR (151 MHz, DMSO-d_6) δ 169.08, 166.34 (C-13, 16), 101.97 - 101.66 (C-1/7), 83.93 - 82.03 (C-4/10), 73.30 - 69.83 (C-2/8, 3/9, 5/11, 14, 15), 60.20 - 59.95 (C-6), 39.00 (C-12).

MALDI-MS (+; DHB; H_2O/ACN) (m/z): Calculated for $[C_{116}H_{188}N_4O_{92}Na]^+$: 3133.01; found: 3133.02.

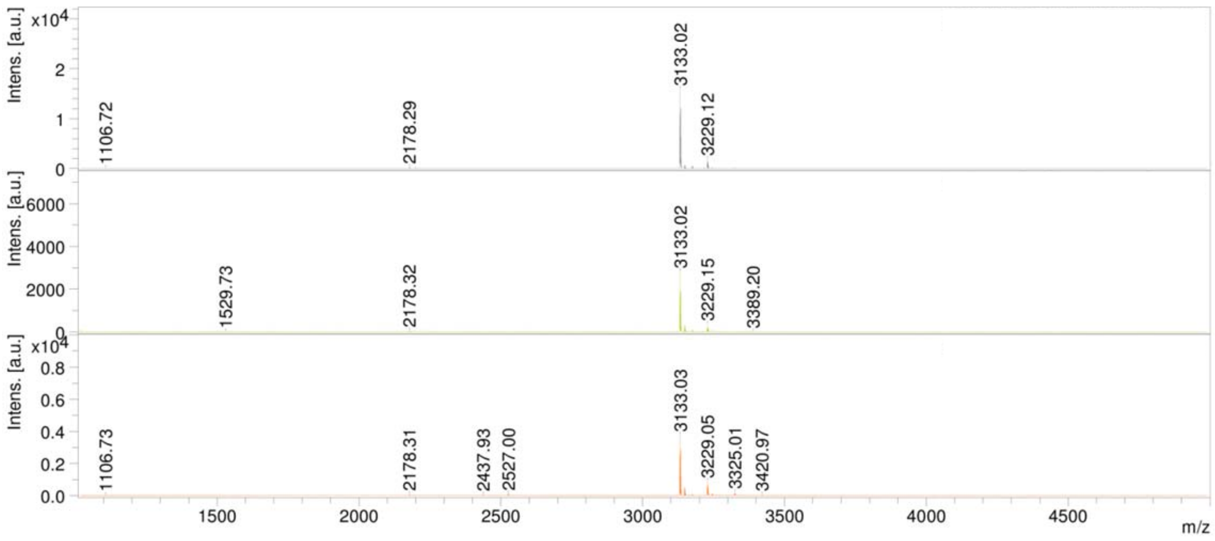

Figure S16: MALDI mass spectra of **4** (matrix: DHB, solvent for preparation: H_2O/ACN).

Cyclodextrin sequence *α-β-α*-$(OH)_{53}$ (**5**)

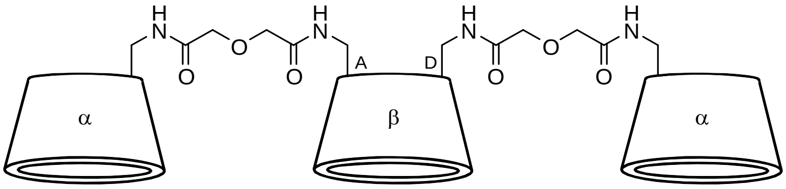

47 (59.4 mg, 10.8 µmol) was dissolved in MeOH (p.a., 5 mL) and sodium methoxide (cat.) was added. After stirring for 36 h at room temperature the solution was neutralized by addition of ion exchange resin (*Dowex*® *HCR-W2 hydrogen form*), stirred for 1 h, filtered and evaporated. After freeze drying the product was obtained as white solid.

Yield: quant. (35.7 mg, 10.9 µmol).

Empirical formula (MW in g/mol): $C_{122}H_{198}N_4O_{97}$ (3272.85).

^{1}H NMR (600 MHz, DMSO-d_6) δ 5.90 - 5.35 (m, 38H, 2/8, 3/9-O*H*), 4.94 - 4.71 (m, 19H, H-1/7), 4.58 - 4.34 (m, 15H, 6-O*H*), 4.07 - 3.49 (m, 80H, H-3/9, 5/11, 6, 12, 14, 15), 3.47 - 3.16 (m, 42H, H-2/8, 4/10, 12').

^{13}C{^{1}H} NMR (151 MHz, DMSO-d_6) δ 169.07, 168.97, 168.92 (C-13, 16), 102.00 - 101.62 (C-1/7), 82.24 - 81.89 (C-4/10), 73.25 - 69.80 (C-2/8, 3/9, 5/11, 14, 15), 60.05 - 59.64 (C-6), 39.00 (C-12).

MALDI-MS (+; DHB; H_2O/ACN) (m/z): Calculated for $[C_{122}H_{198}N_4O_{97}Na]^+$: 3295.06; found: 3295.05.

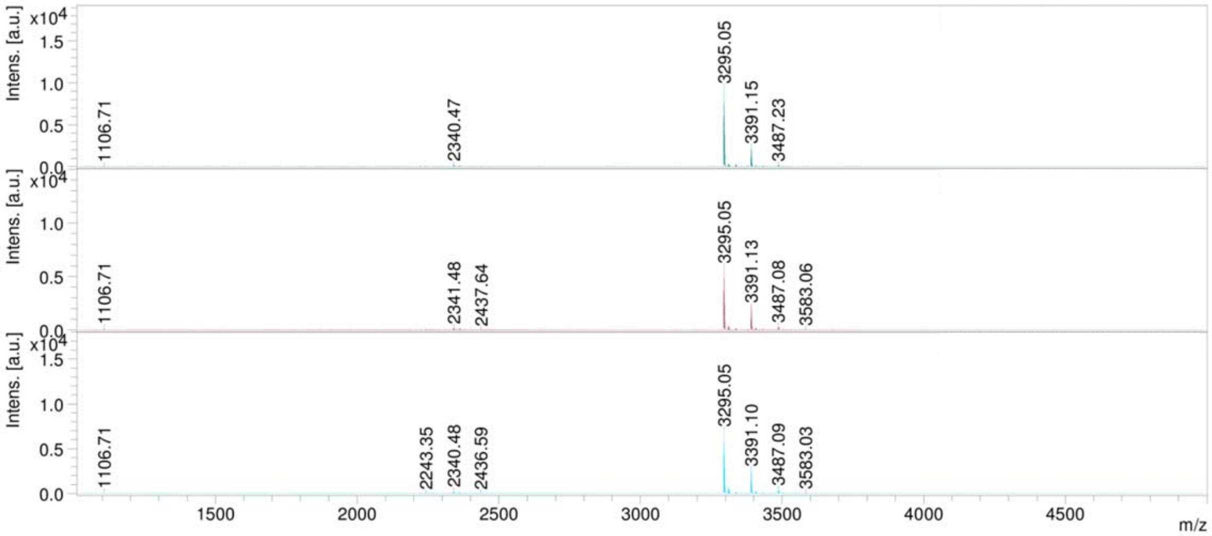

Figure S17: MALDI mass spectra of **5** (matrix: DHB, solvent for preparation: H_2O/ACN).

Cyclodextrin sequence *β-α-β*-(OH)$_{56}$ (**6**)

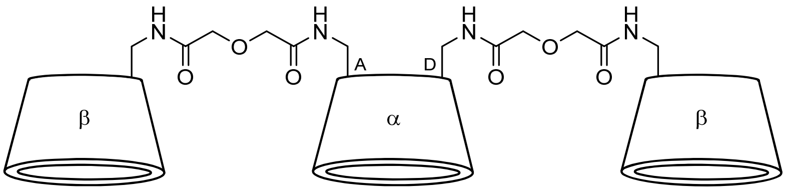

48 (106 mg, 18.3 µmol) was dissolved in MeOH (p.a., 5 mL) and sodium methoxide (cat.) was added. After stirring for 36 h at room temperature the solution was neutralized by addition of ion exchange resin (*Dowex® HCR-W2 hydrogen form*), stirred for 1 h, filtered and evaporated. After freeze drying the product was obtained as white solid.

Yield: quant. (62.9 mg, 18.3 µmol).

Empirical formula (MW in g/mol): $C_{128}H_{208}N_4O_{102}$ (3434.99).

^{1}H NMR (600 MHz, DMSO-d_6) δ 5.68 (s, 40H, 2/8, 3/9-O*H*), 4.97 - 4.76 (m, 20H, H-1/7), 4.06 - 3.48 (m, 84H, H-3/9, 5/11, 6, 12, 14, 15), 3.48 - 3.12 (m, 44H, H-2/8, 4/10, 12’).

^{13}C{^{1}H} NMR (151 MHz, DMSO-d_6) δ 169.54, 169.37 (C-13, 16), 102.65 - 102.12 (C-1/7), 84.29 - 81.75 (C-4/10), 73.72 - 70.14 (C-2/8, 3/9, 5/11, 14, 15), 60.43 - 60.23 (C-6), 39.13, 38.88 (C-12).

MALDI-MS (+; DHB; H_2O/ACN) (m/z): Calculated for $[C_{128}H_{208}N_4O_{102}Na]^+$: 3457.11; found: 3457.09.

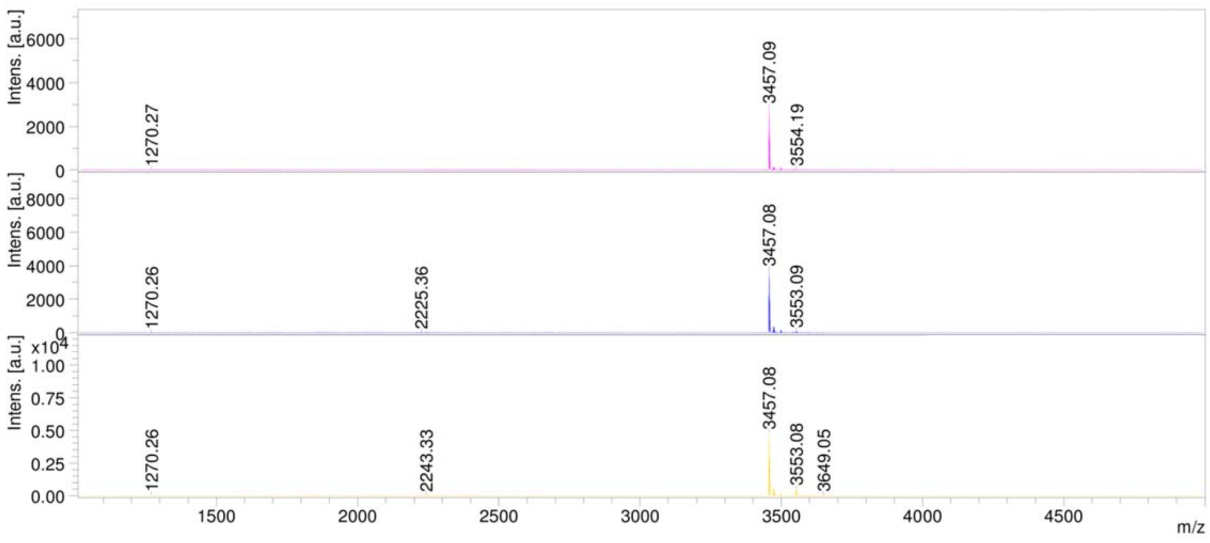

Figure S18: MALDI mass spectra of **6** (matrix: DHB, solvent for preparation: H_2O/ACN).

Cyclodextrin sequence *β*-*β*-*β*-$(OH)_{59}$ (**7**)

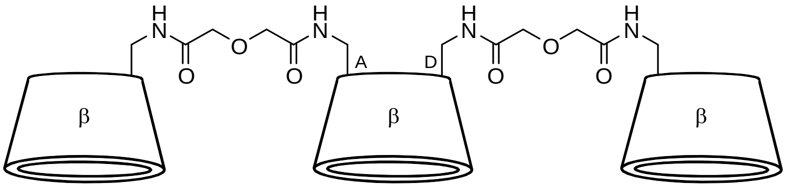

49 (98.1 mg, 16.1 µmol) was dissolved in MeOH (p.a., 5 mL) and sodium methoxide (cat.) was added. After stirring for 36 h at room temperature the solution was neutralized by addition of ion exchange resin (*Dowex® HCR-W2 hydrogen form*), stirred for 1 h, filtered and evaporated. After freeze drying the product was obtained as white solid.

Yield: quant. (58.0 mg, 16.1 µmol).

Empirical formula (MW in g/mol): $C_{134}H_{218}N_4O_{107}$ (3597.14).

^{1}H NMR (600 MHz, DMSO-d_6) δ 5.91 - 5.58 (m, 42H, 2/8, 3/9-O*H*), 4.94 - 4.75 (m, 21H, H-1/7), 4.43 (s, 17H, 6-O*H*), 4.06 - 3.47 (m, 88H, H-3/, 5/11, 6, 12, 14, 15), 3.47 - 3.08 (m, 46H, H-2/8, 4/10, 12').

$^{13}C\{^1H\}$ NMR (151 MHz, DMSO-d_6) δ 168.89 (C-13, 16), 102.20 - 101.71 (C-1/7), 83.81 - 81.30 (C-4/10), 73.16 - 69.71 (C-2/8, 3/9, 5/11, 14, 15), 60.01 - 59.75 (C-6), 39.13 (C-12).

MALDI-MS (+; DHB; H_2O/ACN) (m/z): Calculated for $[C_{134}H_{218}N_4O_{107}Na]^+$: 3619.17; found: 3619.14.

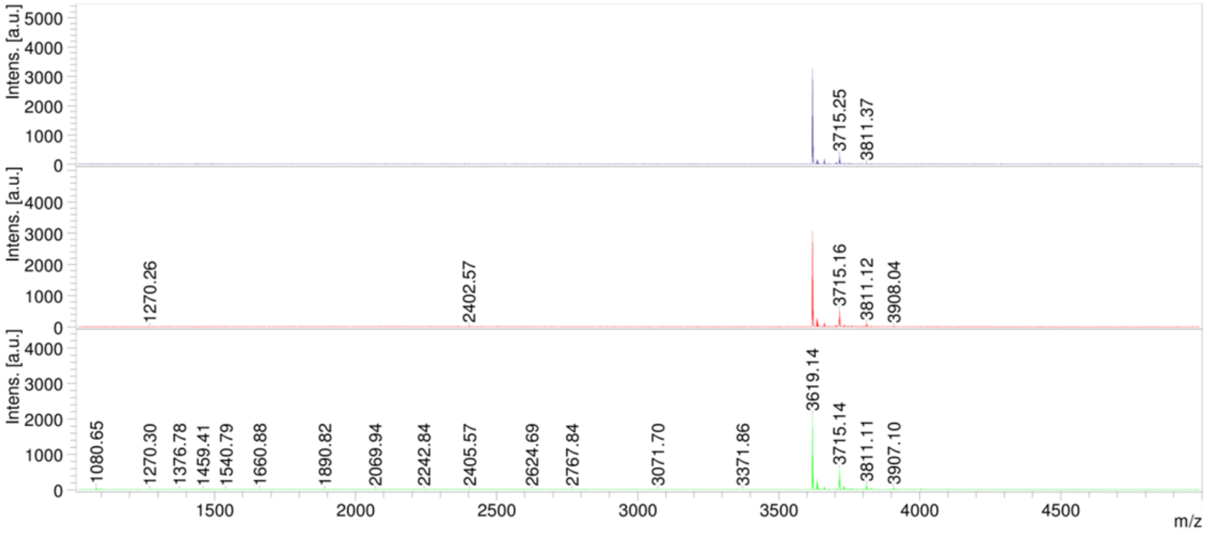

Figure S19: MALDI mass spectra of **7** (matrix: DHB, solvent for preparation: H_2O/ACN).

Synthesis of the di- and trivalent guest sequences

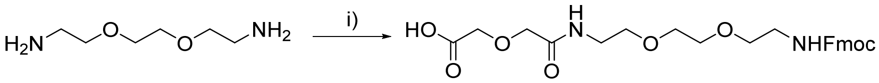

50

Figure S20: Synthesis of **50**: i) a) 2,2'-(Ethylenedioxy)bis(ethylamine), diglycolic anhydride, ACN, 3 h, r.t., b) Fmoc-OSu, DIPEA, ACN/H_2O, 16 h, r.t., 53%.

50 was prepared as described in literature[7].

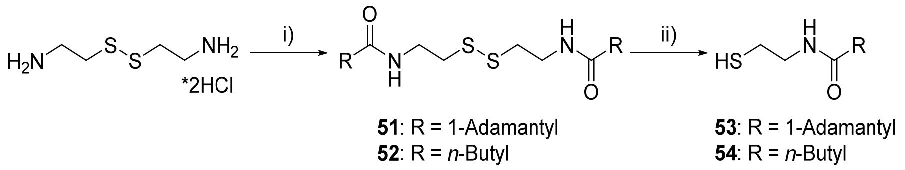

Figure S21: Synthesis of **53** and **54**: i) Cystamine dihydrochloride, 1-adamantanecarboxylic acid/valeric acid, EDC·HCl, HOBt·H_2O, DIPEA, DMF, 3 d/21 h, r.t., 89% (**51**)/78% (**52**); ii) **51**/**52**, (±)-DTT, NEt_3, EtOH, 2 d, reflux, 98% (**53**)/92% (**54**).

52 was prepared as described in literature[8].

(1*S*,1'*S*,3*S*,3'*S*)-*N,N'*-(Disulfanediylbis(ethane-2,1-diyl))bis(adamantane-1-carboxamide) (**51**)

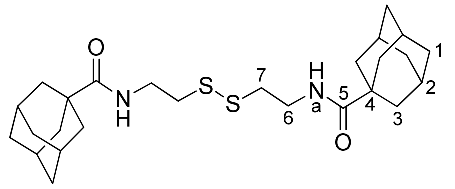

Cystamine dihydrochloride (1.13 g, 5.00 mmol) was suspended in DMF (SPPS grade, 40 mL) and degassed for 15 min by bubbling argon through it. Under stirring HOBt·H_2O (2.99 g, 19.5 mmol), EDC·HCl (3.77 g, 19.7 mmol), 1-adamantancarboxylic acid (3.61 g, 20.0 mmol) and DIPEA (5.24 mL, 30.0 mmol) were added and stirred for 3 d at room temperature. After addition of dH_2O (150 mL) and stirring for 15 min the resulting precipitate was collected by filtration and recrystallized from ethanol. After isolation and drying under reduced pressure the pure product was obtained as white solid.

Yield: 89% (2.13 g, 4.47 mmol).

Empirical formula (MW in g/mol): $C_{26}H_{40}N_2O_2S_2$ (476.74).

^{1}H NMR (400 MHz, $CDCl_3$) δ 6.26 (t, *J* = 5.1 Hz, 2H, H-a), 3.54 (q, *J* = 6.2 Hz, 4H, H-7), 2.81 (t, *J* = 6.4 Hz, 4H, H-6), 2.03 (s, 6H, H-2), 1.92 - 1.78 (m, 12H, H-3), 1.77 - 1.62 (m, 12H, H-1).

^{13}C{^{1}H} NMR (101 MHz, $CDCl_3$) δ 178.42 (C-5), 40.77 (C-4), 39.33 (C-3), 38.29 (C-7), 38.06 (C-6), 36.62 (C-1), 28.23 (C-2).

MS-ESI-EM (+) (m/z): Calculated for $[C_{26}H_{40}N_2O_2S_2Na]^+$: 499.2423; found: 499.2417. Calculated for $[(C_{26}H_{40}N_2O_2S_2)_2Na]^+$: 975.4955; found: 975.4924.

(1*S*,3*S*)-*N*-(2-Mercaptoethyl)adamantane-1-carboxamide (**53**)

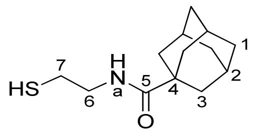

(±)-DTT (631 mg, 4.10 mmol) and NEt_3 (546 µL, 4.10 mmol) were added to a degassed suspension of **51** (1.50 g, 3.15 mmol) in ethanol (abs., 100 mL) and refluxed for 2 d. All volatile components were evaporated and the residue was taken up in DCM (300 mL), washed with aqueous $NaHCO_3$ (sat., 150 mL), dH_2O (150 mL) and brine (150 mL), dried over $MgSO_4$, filtered and evaporated to yield the pure thiol as white solid.

Yield: 98% (1.47 g, 6.16 mmol).

Empirical formula (MW in g/mol): $C_{13}H_{21}NOS$ (239.38).

^{1}H NMR (300 MHz, $CDCl_3$) δ 6.09 (s, 1H, H-a), 3.49 - 3.33 (m, 2H, H-6), 2.65 (dt, *J* = 8.5, 6.3 Hz, 2H, H-7), 2.03 (s, 3H, H-2), 1.85 (d, *J* = 2.8 Hz, 6H, H-3), 1.78 - 1.62 (m, 6H, H-1), 1.31 (t, *J* = 8.5 Hz, 1H, 7-S*H*).

$^{13}C\{^1H\}$ NMR (75 MHz, $CDCl_3$) δ 178.23 (C-5), 42.06 (C-6), 40.79 (C-4), 39.35, 36.58 (C-1, 3), 28.19 (C-2), 24.88 (C-7).

MS-ESI-EM (+) (m/z): Calculated for $[C_{13}H_{21}NOSH]^+$: 240.1417; found: 240.1419. Calculated for $[C_{13}H_{21}NOSNa]^+$: 262.1236; found: 262.1239.

N-(2-mercaptoethyl)pentanamide (**54**)[9]

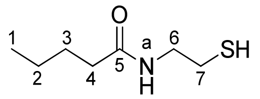

(±)-DTT (750 mg, 4.86 mmol) and NEt_3 (648 μL, 4.68 mmol) were added to a degassed solution of **52** (1.20 g, 3.74 mmol) in ethanol (abs., 100 mL) and refluxed for 2 d. All volatile components were evaporated and the residue was taken up in DCM (300 mL), washed with aqueous $NaHCO_3$ (sat., 150 mL), dH_2O (150 mL) and brine (150 mL), dried over $MgSO_4$, filtered and evaporated to yield the pure thiol as yellow liquid.

Yield: 92% (1.11 g, 6.86 mmol).

Empirical formula (MW in g/mol): $C_7H_{15}NOS$ (161.27).

^1H NMR (300 MHz, $CDCl_3$) δ 5.98 (s, 1H, H-a), 3.49 - 3.38 (m, 2H, H-6), 2.73 - 2.60 (m, 2H, H-7), 2.29 - 2.11 (m, 2H, H-4), 1.73 - 1.54 (m, 2H, H-3), 1.43 - 1.24 (m, 3H, H-2, 7-S*H*), 0.91 (t, J = 7.3 Hz, 3H, H-1).

$^{13}C\{^1H\}$ NMR (75 MHz, $CDCl_3$) δ 173.54 (C-5), 42.38 (C-6), 36.49 (C-4), 27.88 (C-3), 24.70 (C-7), 22.44 (C-2), 13.86 (C-1).

MS-ESI-EM (+) (m/z): Calculated for $[C_7H_{15}NOSH]^+$: 162.0947; found: 162.0946. Calculated for $[C_7H_{15}NOSNa]^+$: 184.0767; found: 184.0767.

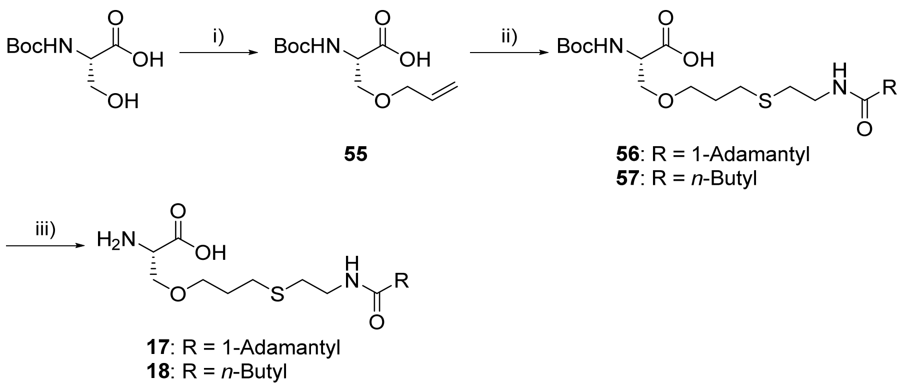

Figure S22: Synthesis of **17** and **18**: i) Boc-*L*-serine-OH, NaH, allyl bromide, 18 h, r.t., 82%; ii) **55**, **53**/**54**, DMPA, EtOH, h□ (365 nm), 21 h, r.t., 53% (**56**)/78% (**57**); iii) **56**/**57**, TFA, DCM, 2 h, R.T., 93% (**17**)/91% (**18**).

55 was prepared as described in literature[10].

O-(3-((2-((3*S*,5*S*,7*S*)-adamantane-1-carboxamido)ethyl)thio)propyl)-*N*-(*tert*-butoxycarbonyl)-*L*-serine (**56**)

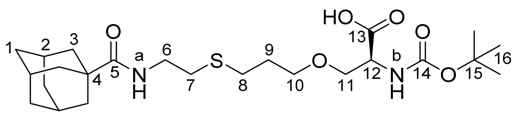

DMPA (42.1 mg, 0.16 mmol) was added to a degassed suspension of **53** (780 mg, 3.26 mmol) and **55** (200 mg, 0.82 mmol) in ethanol (abs., 10 mL). The solution was irradiated at 365 nm and stirred for 21 h at room temperature. The solvent was evaporated and the crude product was purified by column chromatography (silica, EtOAc → EtOAc/HCOOH 1:0.01) to obtain the product as pale yellow solid.

Yield: 53% (210 mg, 0.43 mmol).

Empirical formula (MW in g/mol): $C_{24}H_{40}N_2O_6S$ (484.65).

R_f-value: 0.61 (silica, EtOAc/HCOOH 1:0.01).

^{1}H NMR (400 MHz, $CDCl_3$) δ 6.11 (s, 1H, H-a), 5.44 (d, *J* = 8.4 Hz, 1H, H-b), 4.43 - 4.32 (m, 1H, H-12), 3.82 (dd, *J* = 9.0, 2.9 Hz, 1H, H-11), 3.65 - 3.49 (m, 2H, H-11', 10), 3.45 - 3.28 (m, 3H, H-7, 10'), 2.63 - 2.44 (m, 4H, H-6, 8), 2.02 - 1.93 (m, 3H, H-2), 1.79 (d, *J* = 2.9 Hz, 6H, H-3), 1.76 - 1.58 (m, 8H, H-1, 9), 1.38 (s, 9H, H-16).

$^{13}C\{^{1}H\}$ NMR (101 MHz, $CDCl_3$) δ 179.82 (C-5), 172.38 (C-13), 155.67 (C-14), 80.03 (C-15), 71.12 (C-11), 68.84 (C-10), 54.16 (C-12), 40.86 (C-4), 39.23 (C-7), 39.14 (C-3), 36.52 (C-1), 31.74 (C-6), 29.90 (C-9), 28.48 (C-16), 28.28 (C-8), 28.12 (C-2).

MS-ESI-EM (+) (m/z): Calculated for $[C_{24}H_{40}N_2O_6SNa]^+$: 507.2499; found: 507.2506. Calculated for $[(C_{24}H_{40}N_2O_6S)_2Na]^+$: 991.5106; found: 991.5099.

MS-ESI-EM (-) (m/z): Calculated for $[C_{24}H_{39}N_2O_6S]^-$: 483.2523; found: 483.2542.

N-(*tert*-butoxycarbonyl)-*O*-(3-((2-pentanamidoethyl)thio)propyl)-*L*-serine (**57**)

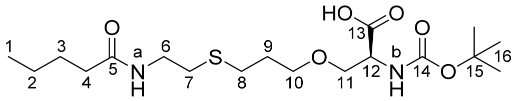

DMPA (88.2 mg, 0.34 mmol) was added to a degassed solution of **54** (1.10 g, 6.84 mmol) and **55** (419 mg, 1.71 mmol) in ethanol (abs., 20 mL). The solution was irradiated at 365 nm and stirred for 21 h at room temperature. The solvent was evaporated and the crude product was purified by column chromatography (silica, EtOAc → EtOAc/HCOOH 1:0.01) to obtain the product as pale yellow, viscous oil.

Yield: 78% (540 mg, 1.33 mmol).

Empirical formula (MW in g/mol): $C_{18}H_{34}N_2O_6S$ (406.54).

R_f-value: 0.56 (silica, EtOAc/HCOOH 1:0.01).

^1H NMR (600 MHz, $CDCl_3$) δ 6.33 (s, 1H, H-a), 5.52 (d, *J* = 8.6 Hz, 1H, H-b), 4.43 (dd, *J* = 7.9, 3.9 Hz, 1H, H-12), 3.90 - 3.84 (m, 1H, H-11), 3.66 (dd, *J* = 9.4, 3.2 Hz, 1H, H-11'), 3.62 - 3.53 (m, 1H, H-10), 3.51 - 3.45 (m, 1H, H-10'), 3.45 - 3.38 (m, 2H, H-7), 2.64 - 2.55 (m, 4H, H-6, 8), 2.25 - 2.19 (m, 2H, H-4), 1.83 - 1.75 (m, 2H, H-9), 1.63 - 1.55 (m, 2H, H-3), 1.44 (s, 9H, H-16), 1.37 - 1.29 (m, 2H, H-2), 0.90 (t, *J* = 7.4 Hz, 3H, H-1).

$^{13}C\{^1H\}$ NMR (151 MHz, $CDCl_3$) δ 175.09 (C-5), 173.47 (C-13), 155.83 (C-14), 80.32 (C-15), 70.87 (C-11), 69.29 (C-10), 54.11 (C-12), 39.09 (C-7), 36.46 (C-4), 31.69 (C-6), 29.60 (C-9), 28.44 (C-16), 28.24 (C-8), 27.82 (C-3), 22.41 (C-2), 13.85 (C-1).

MS-ESI-EM (+) (m/z): Calculated for $[C_{18}H_{34}N_2O_6SNa]^+$: 429.2030; found: 429.2025. Calculated for $[(C_{18}H_{34}N_2O_6S)_2Na]^+$: 835.4167; found: 835.4155.

MS-ESI-EM (-) (m/z): Calculated for $[C_{18}H_{33}N_2O_6S]^-$: 405.2054; found: 405.2062. Calculated for $[(C_{18}H_{33}N_2O_6S)_2H]^-$: 811.4191; found: 811.4212.

O-(3-((2-((3*S*,5*S*,7*S*)-adamantane-1-carboxamido)ethyl)thio)propyl)-*L*-serine (**17**)

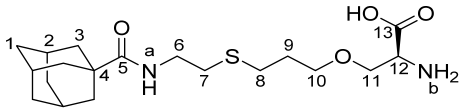

TFA (5 mL) was added to a solution of **56** (195 mg, 0.40 mmol) in DCM (p.a., 5 mL) and stirred for 2 h at room temperature. Toluene (p.a., 20 mL) was added and all volatile components were evaporated. The pure product was obtained after column chromatography (silica, ACN/H_2O 9:1 → 4:1) and freeze drying as white solid.

Yield: 93% (141 mg, 0.37 mmol).

Empirical formula (MW in g/mol): $C_{19}H_{32}N_2O_4S$ (384.53).

R_f-value: 0.32 (silica, ACN/H_2O 4:1).

^1H NMR (600 MHz, DMSO-d_6) δ 7.66 (t, J = 5.7 Hz, 1H, H-a), 7.48 (s, 2H, H-b), 3.68 (dd, J = 10.3, 3.3 Hz, 1H, H-11), 3.56 (dd, J = 10.2, 7.5 Hz, 1H, H-11'), 3.52 - 3.45 (m, 1H. H-10), 3.45 - 3.39 (m, 1H, H-10'), 3.39 - 3.25 (m, 1H, H-12, overlapping with HDO), 3.22 - 3.14 (m, 2H, H-6), 2.58 - 2.54 (m, 2H, H-8), 2.53 - 2.48 (m, 2H, H-7, overlapping with solvent signal), 1.98 - 1.93 (m, 3H, H-2), 1.78 - 1.70 (m, 8H, H-3, 9), 1.70 - 1.60 (m, 6H, H-1).

$^{13}C\{^1H\}$ NMR (151 MHz, DMSO-d_6) δ 176.87 (C-5), 167.12 (C-13), 69.78 (C-11), 68.56 (C-10), 54.37 (C-12), 45.06 (C-4), 38.70 (C-3), 38.68 (C-6), 36.15 (C-1), 30.30 (C-9), 29.29 (C-7), 27.64 (C-2), 27.29 (C-8).

MS-ESI-EM (+) (m/z): Calculated for $[C_{19}H_{32}N_2O_4SH]^+$: 385.2156; found: 385.2157. Calculated for $[C_{19}H_{32}N_2O_4SNa]^+$: 407.1975; found: 407.1975.

MS-ESI-EM (-) (m/z): Calculated for $[C_{19}H_{31}N_2O_4S]^-$: 383.2010; found: 383.2012.

O-(3-((2-pentanamidoethyl)thio)propyl)-*L*-serine (**18**)

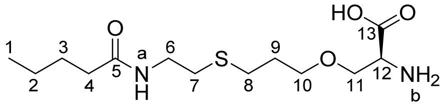

TFA (5 mL) was added to a solution of **57** (498 mg, 1.22 mmol) in DCM (p.a., 5 mL) and stirred for 2 h at room temperature. Toluene (p.a., 20 mL) was added and all volatile components were evaporated. The pure product was obtained after column chromatography (silica, ACN/H_2O 9:1 → 4:1) as white solid.

Yield: 91% (340 mg, 1.11 mmol).

Empirical formula (MW in g/mol): $C_{13}H_{26}N_2O_4S$ (306.42).

R_f-value: 0.28 (silica, ACN/H_2O 4:1).

^1H NMR (600 MHz, DMSO-d_6) δ 8.06 (t, J = 5.7 Hz, 1H, H-a), 7.47 (s, 2H, H-b), 3.68 (dd, J = 10.3, 3.4 Hz, 1H, H-11), 3.55 (dd, J = 10.3, 7.6 Hz, 1H, H-11'), 3.51 - 3.45 (m, 1H, H-10), 3.42 (dt, J = 9.7, 6.2 Hz, 1H, H-10'), 3.36 - 3.28 (m, 1H, H-12, overlapping with HDO), 3.21 - 3.16 (m, 2H, H-6), 2.58 - 2.54 (m, 2H, H-8), 2.54 - 2.50 (m, 2H, H-7, overlapping with solvent signal), 2.05 (t, J = 7.5 Hz, 2H, H-4), 1.76 - 1.70 (m, 2H, H-9), 1.50 - 1.43 (m, 2H, H-3), 1.29 - 1.21 (m, 2H, H-2), 0.85 (t, J = 7.4 Hz, 3H, H-1).

$^{13}C\{^1H\}$ NMR (151 MHz, DMSO-d_6) δ 172.10 (C-5), 166.96 (C-13), 69.74 (C-11), 68.57 (C-10), 54.35 (C-12), 38.55 (C-6), 35.08 (C-4), 30.56 (C-7), 29.24 (C-9), 27.38 (C-3, 8), 21.77 (C-2), 13.72 (C-1).

MS-ESI-EM (+) (m/z): Calculated for $[C_{13}H_{26}N_2O_4SH]^+$: 307.1686; found: 307.1685. Calculated for $[C_{13}H_{26}N_2O_4SNa]^+$: 329.1505; found: 329.1502.

MS-ESI-EM (-) (m/z): Calculated for $[C_{13}H_{25}N_2O_4S]^-$: 305.1541; found: 305.1531.

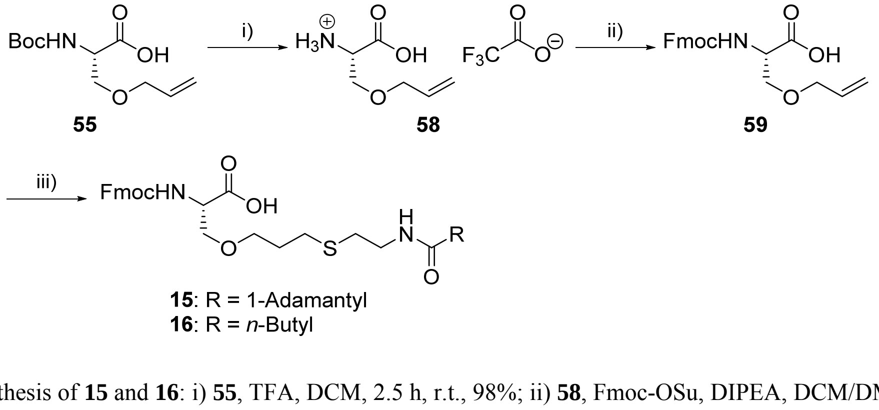

Figure S23: Synthesis of **15** and **16**: i) **55**, TFA, DCM, 2.5 h, r.t., 98%; ii) **58**, Fmoc-OSu, DIPEA, DCM/DMF, 30 min, 0°C, 14 h, r.t., 78%; iii) **59**, **53**/**54**, DMPA, EtOH, h□ (365 nm), 16 h, r.t., 68% (**15**)/65% (**16**).

58 and **59** were prepared as described in literature[11].

N-(((9*H*-fluoren-9-yl)methoxy)carbonyl)-*O*-(3-((2-((3*S*,5*S*,7*S*)-adamantane-1-carboxamido)ethyl)thio)propyl)-*L*-serine (**15**)

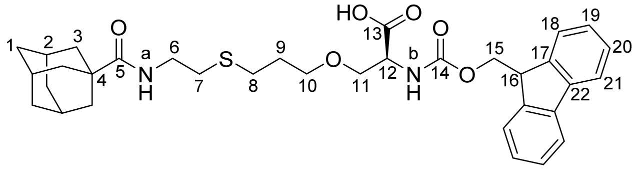

DMPA (140 mg, 0.55 mmol) was added to a degassed suspension of **53** (2.29 g, 9.57 mmol) and **59** (1.00 g, 2.73 mmol) in ethanol (abs., 40 mL). The solution was irradiated at 365 nm and stirred for 16 h at room temperature. The solvent was evaporated and the crude product was purified by column chromatography (silica, EtOAc → EtOAc/HCOOH 1:0.01) to obtain the product as light yellow solid.

Yield: 68% (1.12 g, 1.85 mmol).

Empirical formula (MW in g/mol): $C_{34}H_{42}N_2O_6S$ (606.78).

R_f-value: 0.50 (silica, EtOAc/HCOOH 1:0.01).

^{1}H NMR (600 MHz, DMSO-*d*$_6$) δ 7.89 (d, *J* = 7.6 Hz, 2H, H-21), 7.74 (d, *J* = 7.5 Hz, 2H, H-18), 7.62 (d, *J* = 8.2 Hz, 1H, H-b), 7.47 (t, *J* = 5.6 Hz, 1H, H-a), 7.44 - 7.39 (m, 2H, H-20), 7.35 - 7.30 (m, 2H, H-19), 4.30 - 4.26 (m, 2H, H-15), 4.24 - 4.19 (m, 2H, H-12, 16), 3.68 - 3.60 (m, 2H, H-11), 3.52 - 3.42 (m, 2H, H-10), 3.21 - 3.15 (m, 2H, H-6), 2.56 - 2.51 (m, 4H, H-7, 8, overlapping with solvent signal), 1.97 - 1.92 (m, 3H, H-2), 1.77 - 1.70 (m, 8H, H-3, 9), 1.69 - 1.59 (m, 6H, H-1).

$^{13}C\{^1H\}$ NMR (151 MHz, DMSO-d_6) δ 176.82 (C-5), 171.68 (C-13), 156.03 (C-14), 143.79 (C-17), 140.69 (C-22), 127.60 (C-20), 127.03 (C-19), 125.32 (C-18), 120.07 (C-21), 69.55 (C-11), 68.89 (C-10), 65.77 (C-15), 54.22 (C-12), 46.61 (C-16), 39.77 (C-4), 38.70 (C-3), 38.62 (C-6), 36.14 (C-1), 30.36 (C-7), 29.17 (C-9), 27.62 (C-2), 27.48 (C-8).

MS-ESI-EM (+) (m/z): Calculated for $[C_{34}H_{42}N_2O_6SH]^+$: 607.2836; found: 607.2834. Calculated for $[C_{34}H_{42}N_2O_6SNa]^+$: 629.2656; found: 629.2655.

N-(((9*H*-fluoren-9-yl)methoxy)carbonyl)-*O*-(3-((2-pentanamidoethyl)thio)propyl)-*L*-serine (**16**)

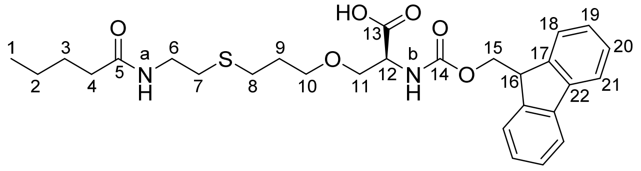

DMPA (79.1 mg, 0.31 mmol) was added to a degassed solution of **54** (876 mg, 5.44 mmol) and **59** (571 mg, 1.55 mmol) in ethanol (abs., 15 mL). The solution was irradiated at 365 nm and stirred for 16 h at room temperature. The solvent was evaporated and the crude product was purified by column chromatography (silica, EtOAc → EtOAc/HCOOH 1:0.01) to obtain the product as light yellow, sticky solid.

Yield: 65% (535 mg, 1.01 mmol).

Empirical formula (MW in g/mol): $C_{28}H_{36}N_2O_6S$ (528.66).

R_f-value: 0.42 (silica, EtOAc/HCOOH 1:0.01).

^1H NMR (600 MHz, DMSO-d_6) δ 7.89 (d, J = 7.4 Hz, 3H, H-21, a), 7.74 (d, J = 7.6 Hz, 2H, H-18), 7.63 (d, J = 8.2 Hz, 1H, H-b), 7.45 - 7.38 (m, 2H, H-20), 7.37 - 7.30 (m, 2H, H-19), 4.32 - 4.25 (m, 2H, H-15), 4.25 - 4.18 (m, 2H, H-12, 16), 3.69 - 3.60 (m, 2H,

H-11), 3.52 - 3.41 (m, 2H, H-10), 3.22 - 3.16 (m, 2H, H-6), 2.56 - 2.51 (m, 4H, H-7, 8, overlapping with solvent signal), 2.04 (t, *J* = 7.5 Hz, 2H, H-4), 1.76 - 1.70 (m, 2H, H-9), 1.49 - 1.41 (m, 2H, H-3), 1.29 - 1.21 (m, 2H, H-2), 0.84 (t, *J* = 7.3 Hz, 3H, H-1).

$^{13}C\{^{1}H\}$ NMR (151 MHz, DMSO-d_6) δ 172.09 (C-5), 171.69 (C-13), 156.04 (C-14), 143.79 (C-17), 140.70 (C-22), 127.62 (C-20), 127.04 (C-19), 125.29 (C-18), 120.08 (C-21), 69.55 (C-11), 68.89 (C-10), 65.77 (C-15), 54.21 (C-12), 46.61 (C-16), 38.49 (C-6), 35.07 (C-4), 30.62 (C-7), 29.17 (C-9), 27.54 (C-8), 27.37 (C-3), 21.76 (C-2), 13.70 (C-1).

MS-ESI-EM (+) (m/z): Calculated for $[C_{28}H_{36}N_2O_6SH]^+$: 529.2367; found: 529.2358. Calculated for $[C_{28}H_{36}N_2O_6SNa]^+$: 551.2186; found: 551.2179.

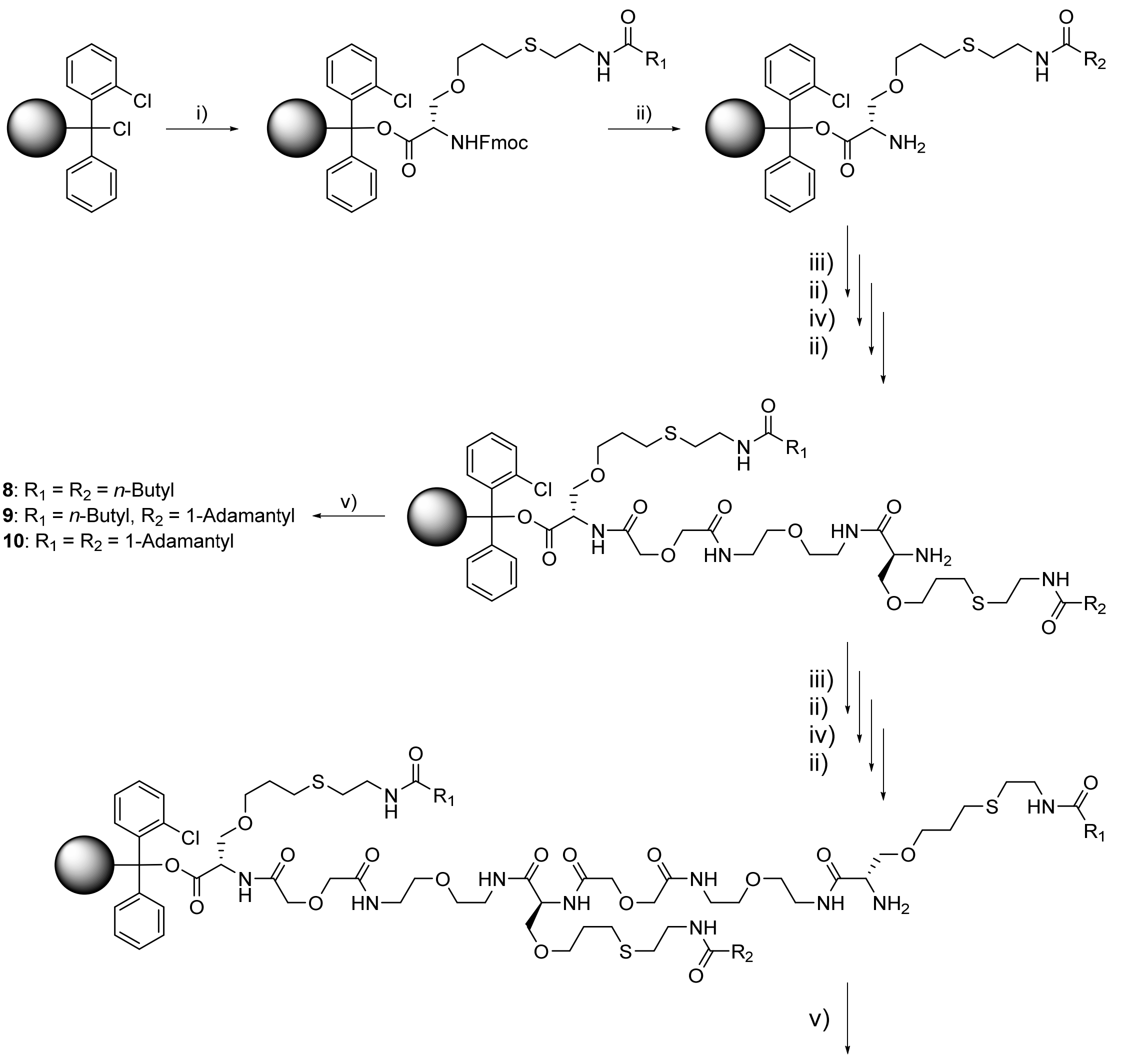

8: $R_1 = R_2 =$ *n*-Butyl
9: $R_1 =$ *n*-Butyl, $R_2 =$ 1-Adamantyl
10: $R_1 = R_2 =$ 1-Adamantyl

11: $R_1 = R_2 =$ *n*-Butyl
12: $R_1 =$ *n*-Butyl, $R_2 =$ 1-Adamantyl
13: $R_1 =$ 1-Adamantyl, $R_2 =$ *n*-Butyl
14: $R_1 = R_2 =$ 1-Adamantyl

Figure S24: Solid-phase peptide synthesis of **8** - **14**: i) 2-CTC resin, **15**/**16**, DIPEA, DCM, 2.5 h, r.t., 72 - 83%; ii) Piperidine, DMF, 20 min, r.t.; iii) **50**, DIPCDI, Oxyma Pure®, DMF, 3.5 - 21 h, r.t.; iv) **15**/**16**, DIPCDI, Oxyma Pure®, DMF, 3.5 - 21 h, r.t.; v) TFA, TIS, ddH_2O, 4 h, r.t.. The chemical structures and the yields of molecules 8 - 14 are given in Figure S25 and Figure S26.

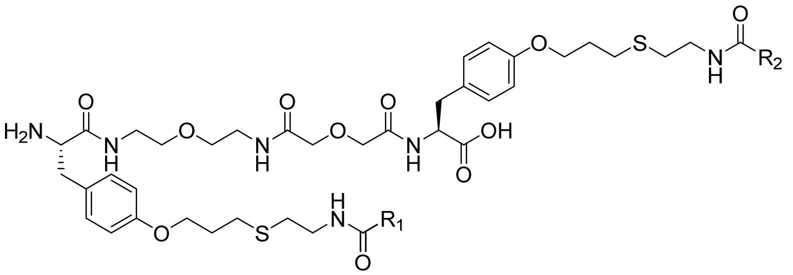

8: R_1 = R_2 = *n*-Butyl; yield: 63%
9: R_1 = 1-Adamantyl, R_2 = *n*-Butyl; yield: 48%
10: R_1 = R_2 = 1-Adamantyl; ; yield: 40%

Figure S25: Chemical structures of the divalent guest strands **8**, **9** and **10** and yields of the SPPS, based on the loading of the resin.

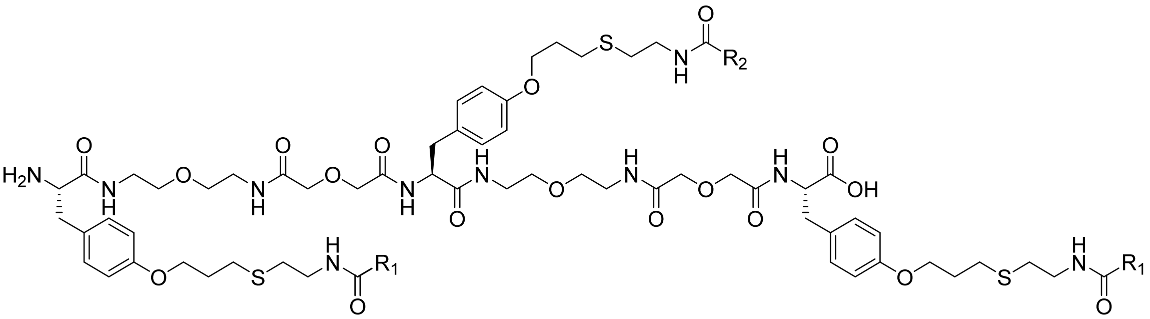

11: R_1 = R_2 = *n*-Butyll; yield: 36%
12: R_1 = *n*-Butyl, R_2 = 1-Adamantyl; yield: 38%
13: R_1 = 1-Adamantyl, R_2 = *n*-Butyl; yield: 44%
14: R_1 = R_2 = 1-Adamantyl; yield: 40%

Figure S26: Chemical structures of the trivalent guest strands **11**, **12**, **13** and **14** and yields of the SPPS, based on the loading of the resin.

Solid phase peptide synthesis (GP1)

Coupling of first amino acid to 2CTC-resin[12–15]: Under argon atmosphere the first amino acid (2.0 eq. relative to maximal loading of the resin) dissolved in dry DCM (5 mL/100 mg resin) was added to the 2CTC-resin (loading: 1.6 mmol/g) in a sintered glass funnel and agitated by a gentle stream of argon. DIPEA (1 eq. relative to maximal loading of the resin) was added and after 5 min a second amount of DIPEA (3 eq. relative to maximal loading of the resin) was put into the solution and the mixture was further agitated by an argon stream for 2.5 h. MeOH (p.a., 0.5 mL/100 mg resin) was added and after agitation for 20 min the solution was

drained. The resin was washed consecutively with DCM (p.a.), DMF (SPPS grade), DCM (p.a.) and MeOH (p.a.) three times and dried *in vacuo* to constant weight. The loading of the resin was determined gravimetrically as follows:

$$\mathbf{Loading\ [mol]} = \frac{\mathbf{m(resin\ after\ loading\ [g]) - m(resin\ before\ loading\ [g])}}{\mathbf{M\left(coupled\ amino\ acid\ [\frac{g}{mol}]\right) - M(HCl\ [\frac{g}{mol}])}}$$

Formula 1: Gravimetrical calculation of resin loading after coupling of the first amino acid.

<u>Elongation of the peptide[12,16–18]:</u> The SPPS was done following a standard Fmoc-protocol. The dried resin was swollen with DMF (p.a., 5 mL/100 mg resin) for 30 min. Afterwards the solvent was drained and the Fmoc-group of the first amino acid was cleaved. DMF/piperidin (80%/20%, v/v, 5 mL/100 mg resin) was added to the resin and gently shaken for 20 min. The solvent was drained and the resin was washed with DMF (p.a., 5 mL/100 mg resin) four times and consecutively with DCM (p.a., 5 mL/100 mg resin) and isopropanol (p.a., 5 mL/100 mg resin) two times. The successful removal of the Fmoc-group was verified by a "Kaiser-test"[12,19]. Therefore equal amounts (5 drops) of a ninhydrin solution (5%) in EtOH, a phenol solution (80%) in EtOH and a KCN solution (1 mM) in pyridine were added to a few beads of the resin and heated to 100 °C for 1 min. The successful deprotection of the Fmoc-group was shown by a blue coloring of the beads and the elongation was continued. If the beads showed no staining the deprotection was repeated as described.

After successful deprotection a solution of the next amino acid (2 - 3 eq. relative to maximal loading of the resin), Oxyma Pure® (3.6 eq. relative to maximal loading of the resin) and DIPCDI (3.3 eq. relative to maximal loading of the resin) dissolved in DMF (SPPS grade, 2.5 mL/100 mg resin) was added to the resin and shaken for 3.5-21 h. After draining the solution the resin was washed with DMF (SPPS grade, 5 mL/100 mg resin) four times. To verify complete coupling a "Kaiser-test" was done as described above. Here the beads mustn't

change their colour to show complete coupling. If some beads turned blue the coupling reaction was repeated as described.

After coupling of the last amino acid and cleavage of the Fmoc-group the resin was dried *in vacuo*.

<u>Cleavage of the peptide from the resin, isolation and purification[12,20,21]:</u> TFA (2.5 mL/100 mg resin) containing dH_2O (1.5%) and tri*iso*propylsilane (1.5%) was added to the resin and stirred for 4 h. The solution was isolated, the beads were washed with TFA (2.5 mL/100 mg resin) two times and the combined organic phase was evaporated until almost all TFA was removed. Ice-cooled diethyl ether was added to the remaining solution, yielding a white precipitate. The mixture was kept at -20 °C overnight and the precipitate was isolated by filtration, washed with ice-cooled diethyl ether and dried *in vacuo*. The desired pure peptide was obtained after individual work up.

H-Ser(*O*~*n*Bu)-**50**-Ser(*O*~*n*Bu)-*OH* (**8**)

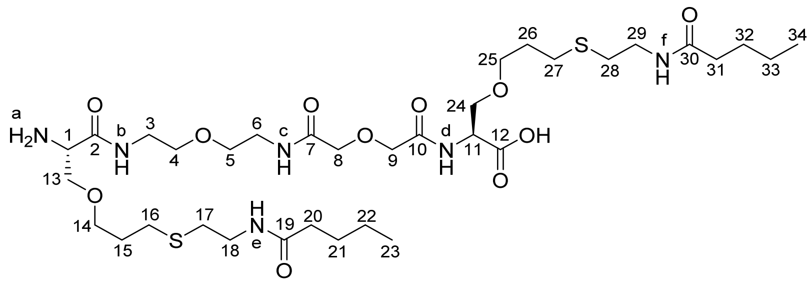

The synthesis was performed as described in **GP1** with following amounts:

1) <u>Coupling of first amino acid:</u> 2CTC-resin (237 mg, 0.38 mmol maximal loading), **16** (400 mg, 0.76 mmol), DIPEA (75 µL, 0.43 mmol and 200 µL, 1.15 mmol). Yield: 80% (0.30 mmol).

2) Elongation: 33% of the resin was used, calculated loading 0.10 mmol. **50** (88.5 mg, 0.20 mmol), **16** (106 mg, 0.20 mmol). For all reactions Oxyma Pure® (0.51 mg, 0.36 mmol) and DIPCDI (51 µL, 0.33 mmol) were used.

3) Purification: The pure peptide was obtained after column chromatography (silica, ACN/H_2O 9:1 → 4:1) and freeze drying as colorless sticky solid.

Yield: 69% (55.3 mg, 69.4 µmol) (based on resin loading in elongation).

Empirical formula (MW in g/mol): $C_{34}H_{64}N_6O_{11}S_2$ (797.04).

R_f-value: 0.24 (silica, ACN/H_2O 4:1).

^{1}H NMR (600 MHz, DMSO-d_6) δ 8.37 (brs, 1H, H-b), 8.10 (t, J = 5.7 Hz, 1H, H-e/f), 7.98 (t, J = 5.8 Hz, 1H, H-e/f), 7.93 (t, J = 5.7 Hz, 1H, H-c), 7.69 (d, J = 7.1 Hz, 1H, H-d), 4.02 (dd, J = 7.5, 4.1 Hz, 1H, H-11), 4.00 - 3.91 (m, 4H, H-8, 9), 3.67 - 3.61 (m, 2H, H-24), 3.56 - 3.34 (m, 11H, H-1, 4, 5, 13, 14, 25), 3.30 - 3.25 (m, 2H, H-6), 3.25 - 3.21 (m, 2H, H-3), 3.21 - 3.15 (m, 4H, H-18, 29), 2.55 - 2.46 (m, 8H, H-16, 17, 27, 28, overlapping with solvent signal), 2.07 - 2.02 (m, 4H, H-20, 31), 1.74 - 1.64 (m, 4H, H-15, 26), 1.50 - 1.42 (m, 4H, H-21, 32), 1.30 - 1.21 (m, 4H, H-22, 33), 0.87 - 0.83 (m, 6H, H-23, 34).

^{13}C{^{1}H} NMR (151 MHz, DMSO-d_6) δ 172.10 (C-19, 30), 172.08 (C-2), 171.39 (C-12), 168.56 (C-7), 167.55 (C-10), 71.90 (C-13), 70.78 (C-24), 70.30, 70.21 (C-8, 9), 68.81, 68.80, 68.70, 68.46 (C-4, 5, 14, 15), 53.97 (C-1), 53.87 (C-11), 38.62 (C-3), 38.55, 38.52 (C-18, 29), 38.20 (C-6), 35.08, 35.06 (C-20, 31), 30.61 (C-17, 28), 29.42, 29.18 (C-15, 26), 27.55, 27.44 (C-16, 27), 27.38 (C-21, 32), 21.78, 21.77 (C-22, 33), 13.72 (C-23, 34).

MALDI-MS (+; DHB; H_2O/ACN) (m/z): Calculated for $[C_{34}H_{64}N_6O_{11}S_2H]^+$: 797.41; found: 797.48. Calculated for $[C_{34}H_{64}N_6O_{11}S_2Na]^+$: 819.40; found: 819.39.

MS-ESI (+) (m/z): Calculated for $[C_{34}H_{64}N_6O_{11}S_2Na]^+$: 819.39667; found: 819.39716.

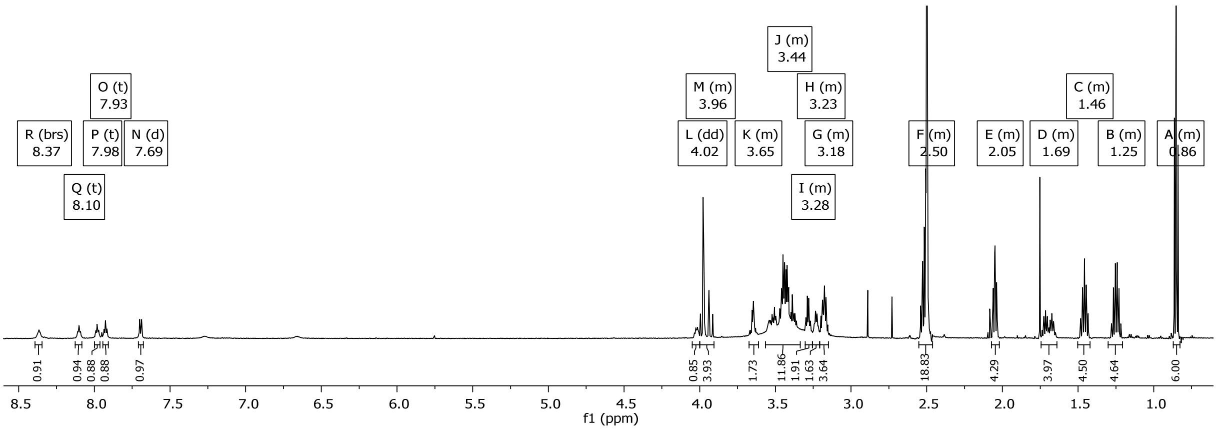

Figure S27: ^{1}H NMR spectrum of **8** (DMSO-d$_6$, 600 MHz, 298 K).

H-Ser(*O*~[1]Ad)-**50**-Ser(*O*~*n*Bu)-*OH* (**9**)

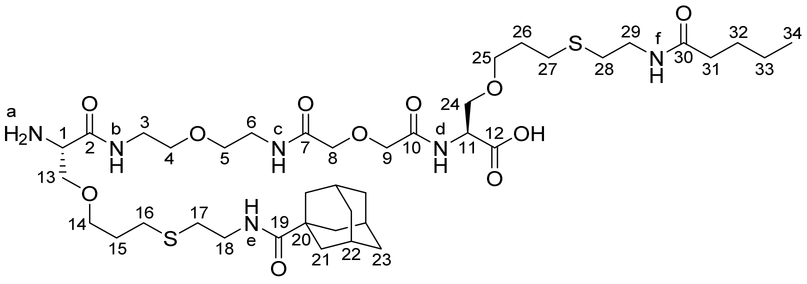

The synthesis was performed as described in **GP1** with following amounts:

1) Coupling of first amino acid: 2CTC-resin (237 mg, 0.38 mmol maximal loading), **16** (400 mg, 0.76 mmol), DIPEA (75 µL, 0.43 mmol and 200 µL, 1.15 mmol). Yield: 80% (0.30 mmol).

2) Elongation: 33% of the resin was used, calculated loading 0.10 mmol. **50** (88.5 mg, 0.20 mmol), **15** (121 mg, 0.20 mmol). For all reactions Oxyma Pure® (51 mg, 0.36 mmol) and DIPCDI (51 µL, 0.33 mmol) were used.

3) Purification: The pure peptide was obtained after column chromatography (silica, ACN/H_2O 9:1 → 4:1) and freeze drying as colorless sticky solid.

Yield: 63% (55.4 mg, 63.3 µmol) (based on resin loading in elongation).

Empirical formula (MW in g/mol): $C_{40}H_{70}N_6O_{11}S_2$ (875.15).

R_f-value: 0.28 (silica, ACN/H_2O 4:1).

^{1}H NMR (600 MHz, DMSO-d_6) δ 8.32 (t, J = 5.7 Hz, 1H, H-b), 8.13 (t, J = 5.6 Hz, 1H, H-f), 7.97 - 7.90 (m, 1H, H-c), 7.68 (d, J = 7.1 Hz, 1H, H-d), 7.54 (t, J = 5.7 Hz, 1H, H-e), 4.04 - 3.89 (m, 5H, H-1, 8, 9), 3.67 - 3.62 (m, 2H, H-24), 3.52 - 3.34 (m, 11H, H-1, 4, 5, 13, 14, 25, overlapping with HDO from solvent), 3.30 - 3.26 (m, 2H, H-6), 3.26 - 3.20 (m, sH, H-3), 3.20 - 3.15 (m, 4H, H-18, 29), 2.54 - 2.47 (m, 8H, H-16, 17, 27, 28, overlapping with solvent signal), 2.05 (t, J = 7.5 Hz, 2H, H-31), 1.97 - 1.93 (m, 3H, H-22), 1.76 - 1.69 (m, 10H, H-15, 21, 26), 1.68 - 1.60 (m, 6H, H-23), 1.49 - 1.43 (m, 2H, H-32), 1.29 - 1.21 (m, 2H, H-33), 0.85 (t, J = 7.4 Hz, 3H, H-34).

^{13}C{^{1}H} NMR (151 MHz, DMSO-d_6) δ 176.84 (C-19), 172.07 (C-30), 171.40 (C-2), 171.06 (C-12), 168.57 (C-7), 167.48 (C-10), 72.14 (C-13), 70.84 (C-24), 70.33, 70.21 (C-8, 9), 68.80, 68.68, 68.41 (C-4, 5, 14, 15), 54.10 (C-1), 53.96 (C-11), 40.45 (C-20), 38.71 (C-21), 38.65, 38.58, 38.56 (C-3, 18, 29), 38.19 (C-6), 36.15 (C-23), 35.08 (C-31), 30.61, 30.36 (C-17, 28), 29.45, 29.20 (C-15, 26), 27.63 (C-22), 27.50, 27.42 (C-16, 27), 27.38 (C-32), 21.78 (C-33), 13.72 (C-34).

MALDI-MS (+; DHB; H_2O/ACN) (m/z): Calculated for $[C_{40}H_{70}N_6O_{11}S_2H]^+$: 875.46; found: 875.51. Calculated for $[C_{40}H_{70}N_6O_{11}S_2Na]^+$: 897.44; found: 897.42.

MS-ESI (+) (m/z): Calculated for $[C_{40}H_{70}N_6O_{11}S_2H]^+$: 875.4617; found: 875.4598. Calculated for $[C_{46}H_{76}N_6O_{11}S_2Na]^+$: 897.4436; found: 897.4417.

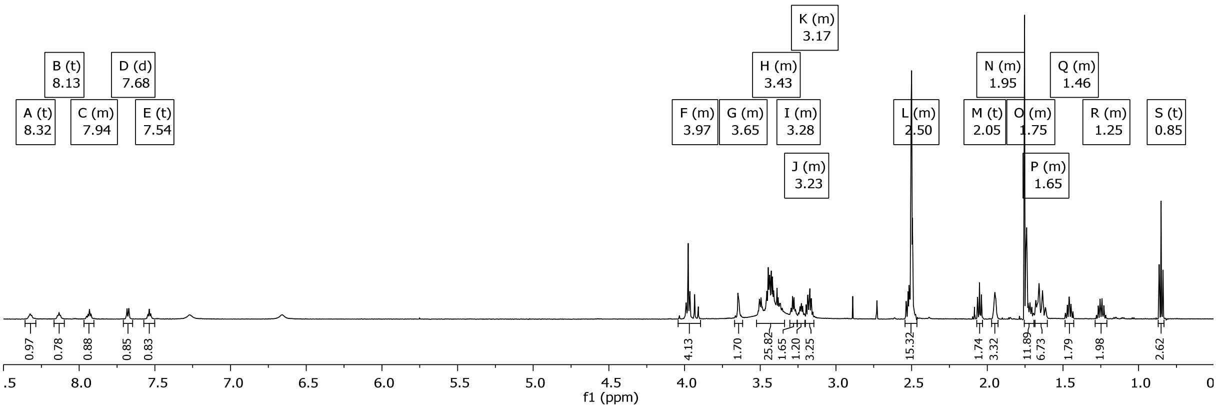

Figure S28: ^{1}H NMR spectrum of **9** (DMSO-d_6, 600 MHz, 298 K).

H-Ser(*O*~^{1}Ad)-**50**-Ser(*O*~^{1}Ad)-*OH* (**10**)

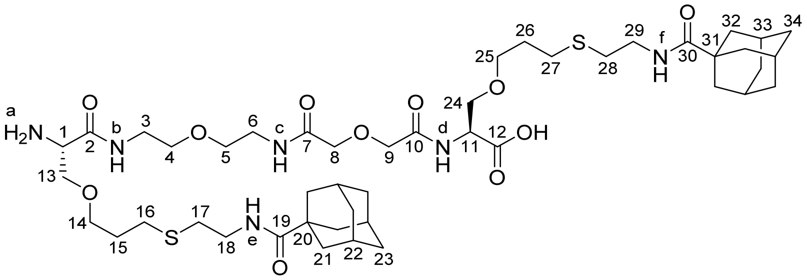

The synthesis was performed as described in **GP1** with following amounts:

1) Coupling of first amino acid: 2CTC-resin (88 mg, 0.14 mmol maximal loading), **15** (169 mg, 0.28 mmol), DIPEA (25 µL, 0.14 mmol and 70 µL, 0.40 mmol). Yield: 76% (0.11 mmol).

2) Elongation: Calculated loading 0.11 mmol. **50** (97.3 mg, 0.22 mmol), **15** (133 mg, 0.22 mmol). For all reactions Oxyma Pure® (56 mg, 0.40 mmol) and DIPCDI (57 µL, 0.36 mmol) were used.

3) Purification: The pure peptide was obtained after column chromatography (silica, ACN/H_2O 9:1 → 4:1) and freeze drying as white solid.

Yield: 57% (60.1 mg, 63.0 µmol) (based on resin loading in elongation).

Empirical formula (MW in g/mol): $C_{46}H_{76}N_6O_{11}S_2$ (953.27).

R_f-value: 0.34 (silica, ACN/H_2O 4:1).

^{1}H NMR (600 MHz, DMSO-d_6) δ 8.35 (brs, 1H, H-b), 7.94 (t, J = 5.9 Hz, 1H, H-c), 7.72 - 7.66 (m, 2H, H-d, e/f), 7.54 (t, J = 5.7 Hz, 1H, H-e/f), 4.04 - 4.00 (m, 1H, H-11), 4.00 - 3.91 (m, 4H, H-8, 9), 3.67 - 3.62 (m, 2H, H-24), 3.55 - 3.35 (m, 11H, H-1, 4, 5, 13, 14, 25), 3.30 - 3.26 (m, 2H, H-6), 3.25 - 3.21 (m, 2H, H-3), 3.20 - 3.14 (m, 4H, H-18, 29), 2.54 - 2.46 (m, 8H, H-16, 17, 27, 28, overlapping with solvent signal), 1.97 - 1.92 (m, 6H, H-22, 33), 1.76 - 1.69 (m, 16H, H-15, 21, 26, 32), 1.69 - 1.60 (m, 12H, H-23, 34).

^{13}C{^{1}H} NMR (151 MHz, DMSO-d_6) δ 176.85 (C-19, 30), 171.41 (C-2), 171.20 (C-12), 168.57 (C-7), 167.53 (C-10), 71.98 (C-13), 70.82 (C-24), 70.32, 70.21 (C-8, 9), 68.81, 68.70, 68.48, C-4, 5, 14, 25), 54.02 (C-1), 53.90 (C-11), 39.30 (C-20, 31), 38.71, 38.70 (C-21, 32), 38.65 (C-18, 29), 38.61 (C-3), 38.20 (C-6), 36.16, 36.15 (C-23, 34), 30.35 (C-17, 28), 29.46, 29.20 (C-15, 26), 27.65, 27.64 (C-22, 33), 27.50, 27.36 (C-16, 27).

MALDI-MS (+; CHCA; H_2O/ACN/TFA) (m/z): Calculated for $[C_{46}H_{76}N_6O_{11}S_2H]^+$: 953.51; found: 953.56. Calculated for $[C_{46}H_{76}N_6O_{11}S_2Na]^+$: 975.49; found: 975.48.

MS-ESI (+) (m/z): Calculated for $[C_{46}H_{76}N_6O_{11}S_2H]^+$: 953.5086; found: 953.5053. Calculated for $[C_{46}H_{76}N_6O_{11}S_2Na]^+$: 975.4906; found: 975.4876.

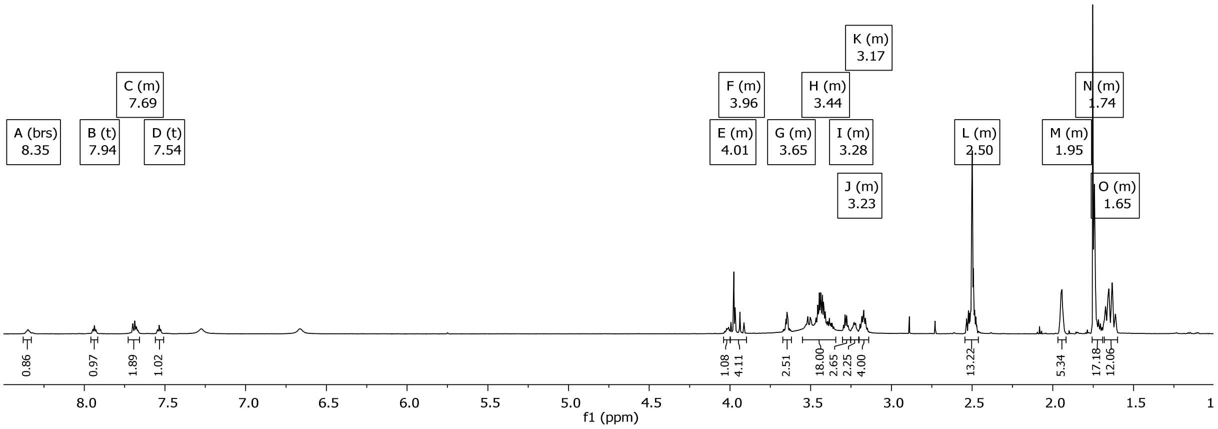

Figure S29: ^{1}H NMR spectrum of **10** (DMSO-d_6, 600 MHz, 298 K).

H-Ser(*O*~*n*Bu)-**50**-Ser(*O*~*n*Bu)-**50**-Ser(*O*~*n*Bu)-*OH* (**11**)

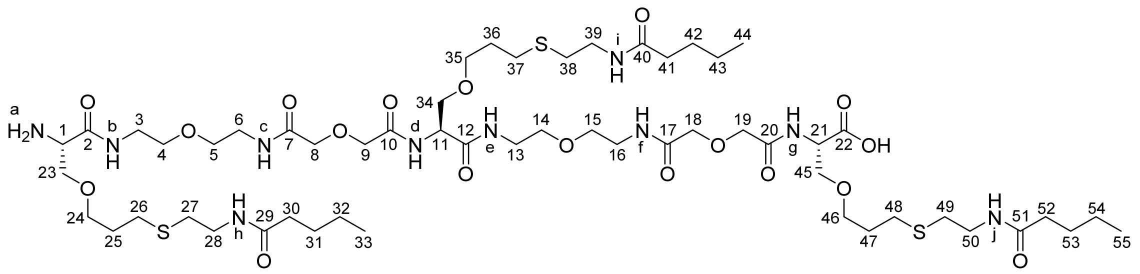

The synthesis was performed as described in **GP1** with following amounts:

1) <u>Coupling of first amino acid:</u> 2CTC-resin (237 mg, 0.38 mmol maximal loading), **16** (400 mg, 0.76 mmol), DIPEA (75 µL, 0.43 mmol and 200 µL, 1.15 mmol). Yield: 80% (0.30 mmol).

2) <u>Elongation:</u> 33% of the resin was used, calculated loading 0.10 mmol. **50** (88.5 mg, 0.20 mmol), **16** (106 mg, 0.20 mmol), **50** (88.5 mg, 0.20 mmol), **16** (106 mg, 0.20 mmol). For all reactions Oxyma Pure® (51 mg, 0.36 mmol) and DIPCDI (51 µL, 0.33 mmol) were used.

3) <u>Purification:</u> The pure peptide was obtained after column chromatography (silica, ACN/H_2O 9:1 → 4:1) and freeze drying as colorless sticky solid.

Yield: 49% (62.8 mg, 48.8 µmol) (based on resin loading in elongation).

Empirical formula (MW in g/mol): $C_{55}H_{102}N_{10}O_{18}S_3$ (1287.65).

R_f-value: 0.18 (silica, ACN/H_2O 4:1).

^{1}H NMR (600 MHz, DMSO-d_6) δ 8.69 (d, J = 8.4 Hz, 1H, H-b), 8.56 - 8.51 (m, 1H, H-c), 8.18 (t, J = 5.8 Hz, 1H, H-d), 8.07 (t, J = 5.8 Hz, 1H, H-e), 8.04 (t, J = 5.8 Hz, 1H, H-h/i/j), 7.98 - 7.93 (m, 1H, H-h/i/j), 7.93 (t, J = 5.8 Hz, 1H, H-h/i/j), 7.85 (t, J = 5.8 Hz, 1H, H-f), 7.72 (d, J = 7.1 Hz, 1H, H-g), 4.52 - 4.45 (m, 1H, H-21), 4.07 - 3.90 (m, 9H, H-8, 9, 11, 18, 19), 3.70 - 3.63 (m, 2H, H-34), 3.62 - 3.54 (m, 2H, H-45), 3.52 - 3.34 (m, 17H, H-1, 4, 5, 14, 15, 23, 24, 35, 46), 3.33 - 3.13 (m, 14H, H-3, 6, 13, 16, 28, 39, 50), 2.55 - 2.45 (m, 12H, H-26, 27, 37, 38, 48, 49, overlapping with solvent signal), 2.05 (t, J = 7.5 Hz, 6H, H-30, 41, 52), 1.74 - 1.63 (m, 6H, H-25, 36, 47), 1.50 - 1.42 (m, 6H, H-31, 42, 53), 1.29 - 1.20 (m, 6H, H-32, 43, 54), 0.85 (t, J = 7.4 Hz, 9H, H-33, 44, 55).

^{13}C{^{1}H} NMR (151 MHz, DMSO-d_6) δ 172.11, 172.08 (C-29, 40, 51), 171.41 (C-12), 169.47 (C-7), 168.93 (C-2), 168.89 (C-10), 168.44 (C-17), 167.50 (C-20), 72.40 (C-23), 70.81 (C-34), 70.28, 70.22, 70.18, 70.10, 70.07 (C-8, 9, 18, 19, 45), 68.90, 68.78, 68.74, 68.73, 68.70, 68.65, 68.59 (C-4, 5, 14, 15, 24, 35, 46), 54.28 (C-1), 53.89 (C-11), 52.92 (C-21), 38.88, 38.53, 38.51, 38.42, 38.32, 38.02 (C-3, 6, 13, 16, 28, 39, 50), 35.08, 35.07 (C-30, 41, 52), 29.38, 29.21, 29.19 (C-27, 38, 49), 27.57, 27.55, 27.48 (C-26, 37, 48), 27.38 (C-31, 42, 53), 21.77 (C-32, 43, 54), 13.71 (C-33, 44, 55).

MALDI-MS (+; DHB; H_2O/ACN) (m/z): Calculated for $[C_{55}H_{102}N_{10}O_{18}S_3H]^+$: 1287.66; found: 1287.65. Calculated for $[C_{55}H_{102}N_{10}O_{18}S_3Na]^+$: 1309.64; found: 1309.66.

MS-ESI (+) (m/z): Calculated for $[C_{55}H_{102}N_{10}O_{18}S_3HNa]^{2+}$: 655.3251; found: 655.3253. Calculated for $[C_{55}H_{102}N_{10}O_{18}S_3Na_2]^{2+}$: 666.3160; found: 666.3167. Calculated for $[C_{55}H_{102}N_{10}O_{18}S_3H]^+$: 1287.6608; found: 12287.6585. Calculated for $[C_{55}H_{102}N_{10}O_{18}S_3Na]^+$: 1309.6428; found: 1309.6406.

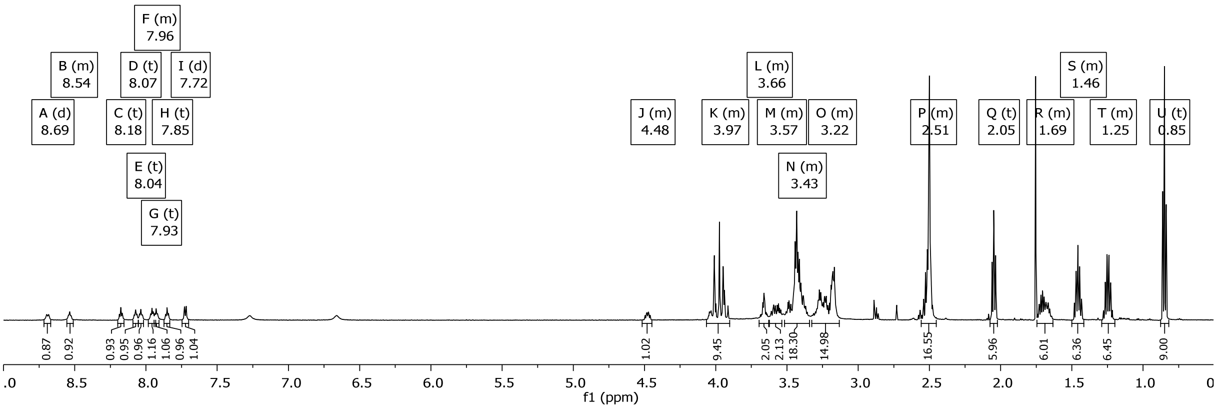

Figure S30: ^1H NMR spectrum of **11** (DMSO-d_6, 600 MHz, 298 K).

H-Ser(*O*~*n*Bu)-**50**-Ser(*O*~^{1}Ad)-**50**-Ser(*O*~*n*Bu)-*OH* (**12**)

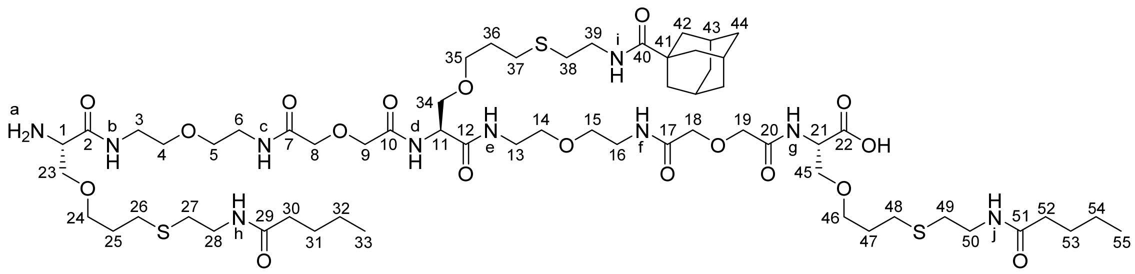

The synthesis was performed as described in **GP1** with following amounts:

1) Coupling of first amino acid: 2CTC-resin (82 mg, 0.13 mmol maximal loading), **16** (142 mg, 0.27 mmol), DIPEA (50 µL, 0.29 mmol and 130 µL, 0.75 mmol). Yield: 72% (0.09 mmol).

2) Elongation: Calculated loading 0.09 mmol. **50** (79.6 mg, 0.18 mmol), **15** (109 mg, 0.18 mmol), **50** (79.6 mg, 0.18 mmol), **16** (95.2 mg, 0.18 mmol). For all reactions Oxyma Pure® (46 mg, 0.32 mmol) and DIPCDI (46 µL, 0.30 mmol) were used.

3) Purification: The pure peptide was obtained after column chromatography (silica, ACN/H_2O 9:1 → 4:1) and freeze drying as colorless sticky solid.

Yield: 39% (48.5 mg, 35.5 µmol) (based on resin loading in elongation).

Empirical formula (MW in g/mol): $C_{61}H_{108}N_{10}O_{18}S_3$ (1365.77).

R_f-value: 0.22 (silica, ACN/H_2O 4:1).

^{1}H NMR (600 MHz, DMSO-d_6) δ 8.76 (s, 1H, H-b), 8.58 (s, 1H, H-c), 8.19 (t, J = 5.8 Hz, 1H, H-d), 8.08 - 8.02 (m, 2H, H-e, h/j), 7.93 (t, J = 5.7 Hz, 1H, H-h/j), 7.84 (t, J = 5.7 Hz, 1H, H-f), 7.71 (d, J = 7.0 Hz, 1H, H-g), 7.51 (t, J = 5.7 Hz, 1H, H-i), 4.51 - 4.45 (m, 1H, H-21), 4.04 - 3.90 (m, 9H, H-8, 9, 11, 18, 19), 3.69 - 3.63 (m, 2H, H-34), 3.63 - 3.54 (m, 2H, H-45), 3.50 - 3.13 (m, 31H, H-1, 3, 4, 5, 6, 13, 14, 154, 16, 23, 24, 28, 35, 39, 46, 50, overlapping with HDO from solvent), 2.55 - 2.47 (m, 12H, H-26, 27, 37, 38, 48, 49, overlapping with solvent signal), 2.07 - 2.03 (m, 4H, H-30, 52), 1.97 - 1.93 (m, 3H, H-43), 1.75 - 1.60 (m, 18H, H-25, 36, 42, 44, 47), 1.50 - 1.42 (m, 4H, H-31, 53), 1.28 - 1.21 (m, 4H, H-32, 54), 0.87 - 0.83 (m, 6H, H-33, 55).

^{13}C{^{1}H} NMR (151 MHz, DMSO-d_6) δ 176.84 (C-40), 172.10, 172.08 (C-29, 51), 171.40 (C-12), 169.49 (C-7), 168.94 (C-2), 168.88 (C-10), 168.41 (C-17), 167.42 (C-20), 72.60 (C-23), 70.86 (C-34), 70.28, 70.22, 70.17, 70.12, 70.07 (C-8, 9, 18, 19, 45), 68.93, 68.76, 68.74, 68.70, 68.65, 68.57 (C-4, 5, 14, 15, 24, 35, 46), 54.38 (C-1), 53.99 (C-11), 52.99 (C-21), 38.89 (C-3/6/13/16), 38.70 (C-42), 38.64, 38.53, 38.51 (C-28, 39, 50), 38.39, 38.34, 38.02 (C-3/6/13/16), 36.15 (C-44), 35.08, 35.07 (C-30, 52), 30.62, 30.35 (C-257, 38, 49), 29.39, 29.22, 29.20 (C-25, 36, 47), 27.64 (C-43), 27.58, 27.49, 27.48 (C-26, 37, 48), 27.38 (C-31, 53), 21.78, 21.77 (C-32, 54), 13.71 (C-33, 55).

MALDI-MS (+; DHB; H_2O/ACN) (m/z): Calculated for $[C_{61}H_{108}N_{10}O_{18}S_3H]^+$: 1365.71; found: 1365.71.

MS-ESI (+) (m/z): Calculated for $[C_{61}H_{108}N_{10}O_{18}S_3Na]^+$: 1387.68974; found: 1387.69129.

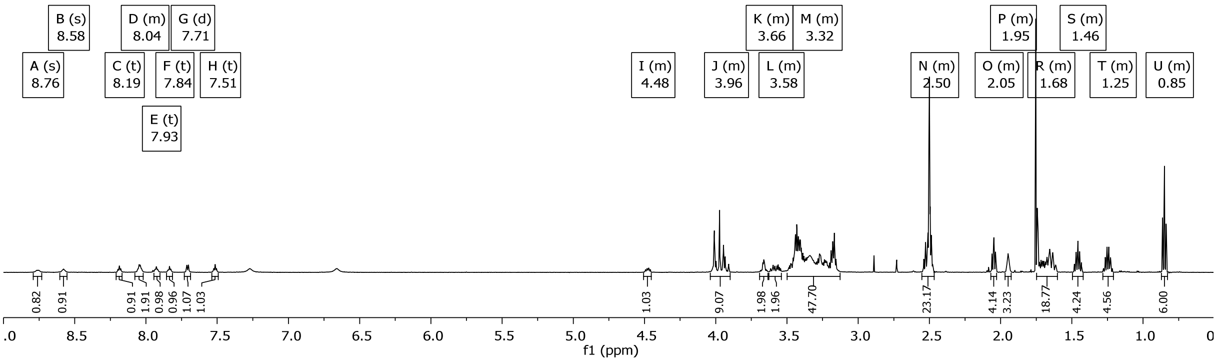

Figure S31: ^{1}H NMR spectrum of **12** (DMSO-d_6, 600 MHz, 298 K).

H-Ser(*O*~^{1}Ad)-**50**-Ser(*O*~*n*Bu)-**50**-Ser(*O*~^{1}Ad)-*OH* (**13**)

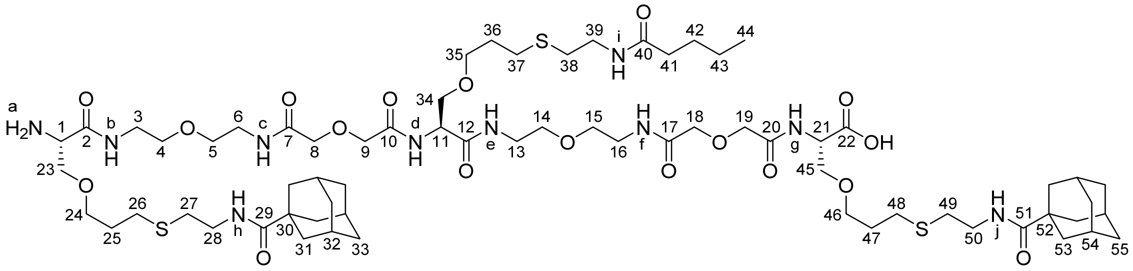

The synthesis was performed as described in **GP1** with following amounts:

1) <u>Coupling of first amino acid:</u> 2CTC-resin (158 mg, 0.25 mmol maximal loading), **15** (303 mg, 0.50 mmol), DIPEA (50 µL, 0.29 mmol and 130 µL, 0.75 mmol). Yield: 83% (0.21 mmol).

2) <u>Elongation:</u> 50% of the resin was used, calculated loading 0.11 mmol. **50** (97.3 mg, 0.22 mmol), **16** (116 mg, 0.22 mmol), **50** (97.3 mg, 0.22 mmol), **15** (133 mg, 0.22 mmol). For all reactions Oxyma Pure® (54 mg, 0.38 mmol) and DIPCDI (57 µL, 0.36 mmol) were used.

3) <u>Purification:</u> The pure peptide was obtained after column chromatography (silica, ACN/H_2O 9:1 → 4:1) and freeze drying as white solid.

Yield: 36% (55.1 mg, 38.1 µmol) (based on resin loading in elongation).

Empirical formula (MW in g/mol): $C_{67}H_{114}N_{10}O_{18}S_3$ (1443.88).

R_f-value: 0.26 (silica, ACN/H_2O 4:1).

^{1}H NMR (600 MHz, DMSO-d_6) δ 8.74 (d, J = 8.4 Hz, 1H, H-b), 8.56 (brs, 1H, H-c), 8.18 (t, J = 5.8 Hz, 1H, H-d), 8.06 (d, J = 6.0 Hz, 1H, H-e), 7.96 (t, J = 4.5 Hz, 1H, H-i), 7.84 (t, J = 5.7 Hz, 1H, H-f), 7.73 (d, J = 7.1 Hz, 1H, H-g), 7.61 (t, J = 5.7 Hz, 1H, H-h/j), 7.50 (t, J = 5.8 Hz, 1H, H-h/j), 4.53 - 4.41 (m, 1H, H-21), 4.05 - 3.91 (m, 9H, H-8, 9, 11, 18, 19), 3.70 - 3.64 (m, 2H, H-34), 3.62 - 3.54 (m, 2H, H-45), 3.53 - 3.13 (m, 31H, H-1, 3, 4, 5, 6, 13, 14, 15, 16, 23, 24, 28, 35, 39, 46, 50, overlapping with HDO from solvent), 2.55 - 2.46 (m, 12H, H-26, 27, 37, 38, 48, 49, overlapping with solvent signal), 2.05 (t, J = 7.5 Hz, 2H, H-41), 1.97 - 1.92 (m, 6H, H-32, 54), 1.76 - 1.69 (m, 18H, H-25, 31, 36, 47, 53), 1.69 - 1.60 (m, 12H, H-33, 55), 1.49 - 1.42 (m, 2H, H-42), 1.28 - 1.21 (m, 2H, H-43), 0.85 (t, J = 7.4 Hz, 3H, H-44).

^{13}C{^{1}H} NMR (151 MHz, DMSO-d_6) δ 176.84 (C-29, 51), 172.10 (C-12, 40), 169.48 (C-7), 168.93 (C-2), 168.88 (C-10), 168.41 (C-17), 167.47 (C-20), 72.44 (C-23), 70.80 (C-34), 70.28, 70.21, 70.18, 70.11, 70.07 (C-8, 9, 18, 19, 45), 68.93, 68.78, 68.74, 68.70, 68.65, 68.61 (C-4, 5, 14, 15, 24, 35, 46), 54.30 (C-1), 53.90 (C-11), 52.98 (C-21), 38.89 (C-3/6/13/16), 38.71, 38.70 (C-31, 53), 38.65, 38.64, 38.51 (C-28, 39, 50), 38.41, 38.34, 38.02 (C-3/6/13/16), 36.16, 36.15 (C-33, 55), 35.07 (C-41), 30.62, 30.37, 30.34 (C-27, 38, 49), 29.40, 29.22, 29.20 (C-25, 36, 47), 27.65, 27.63 (C-32, 54), 27.55, 27.52, 27.39 (C-26, 37, 48), 27.38 (C-42), 21.77 (C-43), 13.71 (C-44).

MALDI-MS (+; DHB; H_2O/ACN) (m/z): Calculated for $[C_{67}H_{114}N_{10}O_{18}S_3H]^+$: 1443.75; found: 1443.76.

MS-ESI (+) (m/z): Calculated for $[C_{67}H_{114}N_{10}O_{18}S_3HNa]^{2+}$: 733.3720; found: 733.3694. Calculated for $[C_{67}H_{114}N_{10}O_{18}S_3Na_2]^{2+}$: 744.3630; found: 744.3607. Calculated for

$[C_{67}H_{114}N_{10}O_{18}S_3H]^+$: 1443.7547; found: 1443.7522. Calculated for $[C_{67}H_{114}N_{10}O_{18}S_3Na]^+$: 1465.7367; found: 1465.7330.

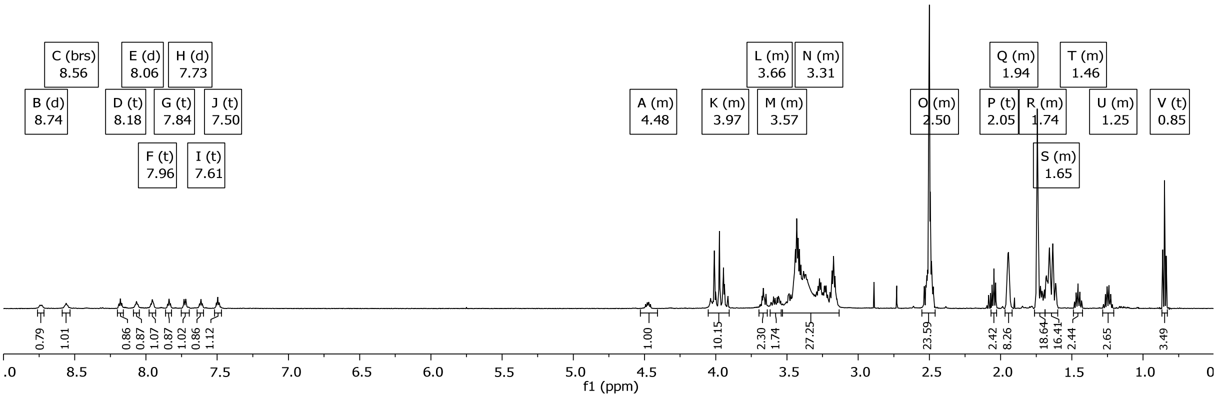

Figure S32: ^{1}H NMR spectrum of **13** (DMSO-d_6, 600 MHz, 298 K).

H-Ser(O~^{1}Ad)-**50**-Ser(O~^{1}Ad)-**50**-Ser(O~^{1}Ad)-OH (**14**)

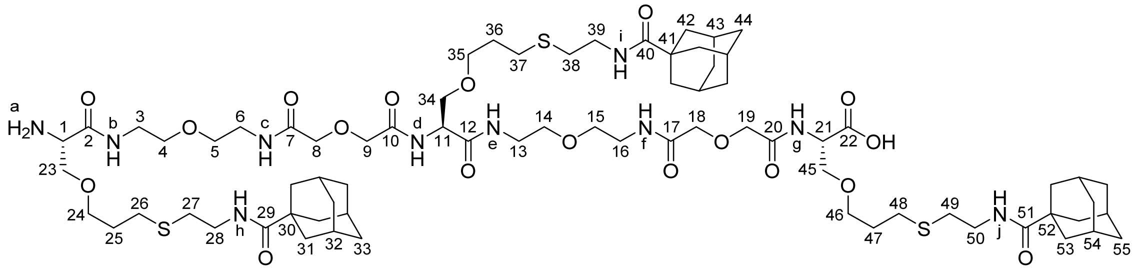

The synthesis was performed as described in **GP1** with following amounts:

1) Coupling of first amino acid: 2CTC-resin (158 mg, 0.25 mmol maximal loading), **15** (303 mg, 0.50 mmol), DIPEA (50 µL, 0.29 mmol and 130 µL, 0.75 mmol). Yield: 83% (0.21 mmol).

2) Elongation: 50% of the resin was used, calculated loading 0.11 mmol. **50** (97.3 mg, 0.22 mmol), **15** (133 mg, 0.22 mmol), **50** (97.3 mg, 0.22 mmol), **15** (133 mg, 0.22 mmol). For all reactions Oxyma Pure® (0.54 mg, 0.38 mmol) and DIPCDI (57 µL, 0.36 mmol) were used.

3) Purification: The pure peptide was obtained after column chromatography (silica, ACN/H_2O 9:1 → 4:1) and freeze drying as white solid.

Yield: 35% (56.2 mg, 36.9 µmol) (based on resin loading in elongation).

Empirical formula (MW in g/mol): $C_{73}H_{120}N_{10}O_{18}S_3$ (1522.00).

R_f-value: 0.30 (silica, ACN/H_2O 4:1).

^{1}H NMR (600 MHz, DMSO-*d*$_6$) δ 8.75 (d, *J* = 8.2 Hz, 1H, H-b), 8.56 (brs, 1H, H-c), 8.18 (t, *J* = 5.8 Hz, 1H, H-d), 8.07 (t, *J* = 5.7 Hz, 1H, H-e), 7.84 (t, *J* = 5.7 Hz, 1H, H-f), 7.73 (d, *J* = 7.1 Hz, 1H, H-g), 7.61 (t, *J* = 5.8 Hz, 1H, H-h/i/j), 7.53 - 7.47 (m, 2H, H-h/i/j), 4.50 - 4.45 (m, 1H, H-21), 4.04 (dd, *J* = 4.3, 2.8 Hz, 1H, H-11), 4.02 - 3.90 (m, 8H, H-8, 9, 18, 19), 3.70 - 3.64 (m, 2H, H-34), 3.62 - 3.54 (m, 2H, H-45), 3.52 - 3.14 (m, 31H, H-1, 3, 4, 5, 6, 13, 14, 15, 16, 23, 24, 28, 35, 39, 46, 50, overlapping with HDO from solvent), 2.55 - 2.46 (m, 14H, H-26, 27, 37, 38, 48, 49, overlapping with solvent signal), 1.98 - 1.92 (m, 9H, H-32, 43, 54), 1.76 - 1.69 (m, 24H, H-25, 31, 36, 42, 47, 53), 1.68 - 1.60 (m, 18H, H-33, 44, 55).

^{13}C{^{1}H} NMR (151 MHz, DMSO-*d*$_6$) δ 176.83 (C-29, 40, 51), 171.61 (C-12), 169.48 (C-7), 168.94 (C-2), 168.88 (C-10), 168.41 (C-17), 167.48 (C-20), 72.40 (C-23), 70.80 (C-34), 70.28, 70.21, 70.18, 70.11, 70.07 (C-8, 9, 18, 19, 45), 68.92, 68.79, 68.76, 68.73, 68.70, 68.65, 68.61 (C-4, 5, 14, 15, 24, 35, 46), 54.28 (C-1), 53.88 (C-11), 52.98 (C-21), 38.89 (C-3/6/13/16), 38.71, 38.70 (C-31, 42, 53), 38.65, 38.64 (C-28, 39, 50), 38.42, 38.34, 38.02 (C-3/6/13/16), 36.16, 36.15 (C-33, 44, 55), 30.37, 30.35, 30.34 (C-27, 38, 49), 29.40, 29.22, 29.20 (C-25, 36, 47), 27.65, 27.64 (C-32, 43, 54), 27.52, 27.49, 27.40 (C-26, 37, 48).

MALDI-MS (+; DHB; H_2O/ACN) (m/z): Calculated for $[C_{73}H_{120}N_{10}O_{18}S_3H]^+$: 1521.80; found: 1521.81. Calculated for $[C_{73}H_{120}N_{10}O_{18}S_3Na]^+$: 1543.78; found: 1543.82.

MS-ESI (+) (m/z): Calculated for $[C_{73}H_{120}N_{10}O_{18}S_3HNa]^{2+}$: 772.3955; found: 772.3945. Calculated for $[C_{73}H_{120}N_{10}O_{18}S_3Na_2]^{2+}$: 783.3864; found: 783.3851. Calculated for $[C_{73}H_{120}N_{10}O_{18}S_3H]^{+}$: 1521.8017; found: 1521.7934. Calculated for $[C_{73}H_{120}N_{10}O_{18}S_3Na]^{+}$: 1543.7836; found: 1543.7730.

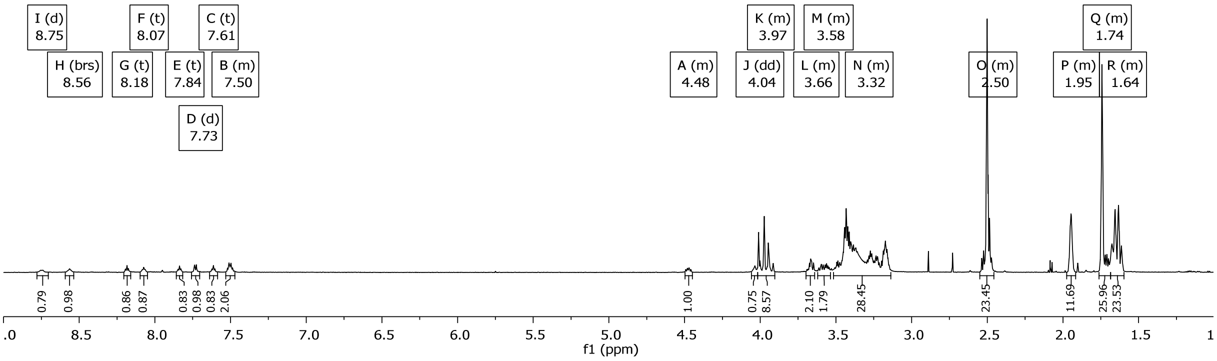

Figure S33: ^{1}H NMR spectrum of **14** (DMSO-d_6, 600 MHz, 298 K).

References

1. Brady, B.; Lynam, N.; O'Sullivan, T.; Ahern, C.; Darcy, R. *Org. Synth.* 2000, *77*, 220–222.

2. Petter, R. C.; Salek, J. S.; Sikorski, C. T.; Kumaravel, G.; Lin, F. T. *J. Am. Chem. Soc.* 1990, *112*, 3860–3868.

3. Sallas, F.; Marsura, A.; Petot, V.; Pintér, I.; Kovács, J.; Jicsinszky, L. *Helv. Chim. Acta* 1998, *81*, 632–645.

4. Lecourt, T.; Herault, A.; Pearce, A. J.; Sollogoub, M.; Sinaÿ, P. *Chem. – Eur. J.* 2004, *10*, 2960–2971.

5. Rousseau, C.; Ortega-Caballero, F.; Nordstrøm, L. U.; Christensen, B.; Petersen, T. E.; Bols, M. *Chem. – Eur. J.* 2005, *11*, 5094–5101.

6. Rawal, G. K.; Zhang, P.; Ling, C.-C. *Org. Lett.* 2010, *12*, 3096–3099.

7. Song, A.; Wang, X.; Zhang, J.; Mařík, J.; Lebrilla, C. B.; Lam, K. S. *Bioorg. Med. Chem. Lett.* 2004, *14*, 161–165.

8. McCaughan, B.; Kay, G.; Knott, R. M.; Cairns, D. *Bioorg. Med. Chem. Lett.* 2008, *18*, 1716–1719.

9. Schuster, M. C.; Mann, D. A.; Buchholz, T. J.; Johnson, K. M.; Thomas, W. D.; Kiessling, L. L. *Org. Lett.* 2003, *5*, 1407–1410.

10. Wagner, H.; Brinks, M. K.; Hirtz, M.; Schäfer, A.; Chi, L.; Studer, A. *Chem. – Eur. J.* 2011, *17*, 9107–9112.

11. Peters, C.; Bacher, M.; Buenemann, C. L.; Kricek, F.; Rondeau, J.-M.; Weigand, K. *ChemBioChem* 2007, *8*, 1785–1789.

12. Chan, W. C.; White, P. D. *Fmoc solid phase peptide synthesis: a practical approach*; Oxford University Press: New York, 2000.

13. *Novabiochem 2004/5 Catalog*; Merck KGaA, Darmstadt, Germany, 2004.

14. Barlos, K.; Gatos, D.; Kallitsis, J.; Papaphotiu, G.; Sotiriu, P.; Wenqing, Y.; Schäfer, W. *Tetrahedron Lett.* 1989, *30*, 3943–3946.

15. Bernhardt, A.; Drewello, M.; Schutkowski, M. *J. Pept. Res.* 1997, *50*, 143–152.

16. Albericio, F.; Carpino, L. A. In *Methods in Enzymology*; Gregg B. Fields, Ed.; Solid-Phase Peptide Synthesis; Academic Press, 1997; Vol. Volume 289, pp. 104–126.

17. Subirós-Funosas, R.; Prohens, R.; Barbas, R.; El-Faham, A.; Albericio, F. *Chem. – Eur. J.* 2009, *15*, 9394–9403.

18. Hudson, D. *J. Org. Chem.* 1988, *53*, 617–624.

19. Kaiser, E.; Colescott, R. L.; Bossinger, C. D.; Cook, P. I. *Anal. Biochem.* 1970, *34*, 595–598.

20. Fields, G. B. In *Peptide Synthesis Protocols*; Pennington, M. W.; Dunn, B. M., Eds.; Methods in Molecular Biology; Humana Press, 1995; pp. 17–27.

21. *Novabiochem Fmoc resin cleavage and deprotection*; Merck KGaA, Darmstadt, Germany, 2007.
