# Supplementary material for: Towards the sequence-specific multivalent molecular recognition of cyclodextrin oligomers
Source: Beilstein J Org Chem. 2014 Oct 20;10:2428–40. doi: 10.3762/bjoc.10.253 (PMC4222322; doi:10.3762/bjoc.10.253)
Supplement: File 2 — ITC Measurements. [file Beilstein_J_Org_Chem-10-2428-s002.pdf]

Supporting Information File 2

for

Towards the sequence-specific multivalent molecular recognition of cyclodextrin oligomers

Michael Kurlemann and Bart Jan Ravoo*

Address: Organic Chemistry Institute, Westfälische Wilhelms-Universität Münster, Corrensstrasse 40, 48149 Münster, Germany

Email: b.j.ravoo@uni-muenster.de

*Corresponding author

ITC Measurements

General

ITC measurements were performed with a *TA Instruments Nano ITC Low Volume* (*Waters Corp.*, Milford, Massachusetts, USA) with a cell volume of 170 µL using *ITCRun Version 2.1.7.0 Firmware version 1.31* (*TA Instruments, Waters Corp.*, Milford, Massachusetts, USA) as software. All titrations were done using a 50 µL syringe and 20 injections of 2.5 µL at a temperature of 25 °C and a stirring rate of 350 rpm. All samples were prepared in 100 mM phosphate buffer pH 7.4 and degassed for 10 minutes before use. The data were analyzed using *NanoAnalyse Data Analysis version 2.36* (*TA Instruments, Waters Corp.*, Milford, Massachusetts, USA), *Microsoft® Excel version 14.07113.5005* as part of *Microsoft® Office Professional Plus 2010* (*Microsoft Corp.*, Redmond, Washington, USA) and *OriginPro 9.1.0G* (*OriginLab Corp.*, Northampton, Massachusetts, USA). Before analysis all data were corrected by subtraction of a dilution measurement of the titrated component into pure solvent.

Basic equations of binding models for analysis of ITC data

1:1 receptor-ligand interaction

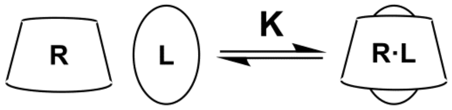

$$K = \frac{[R \cdot L]}{[R][L]}$$

$$[R]_0 = [R] + [R \cdot L] = [R] + K[R][L]$$

$$[L]_0 = [L] + [R \cdot L] = [L] + K[R][L]$$

The binding constant of the 1:1 receptor-ligand interaction is used as intrinsic binding constant K_i in the models of the multivalent systems.

Multivalent interaction of a divalent receptor and a divalent ligand

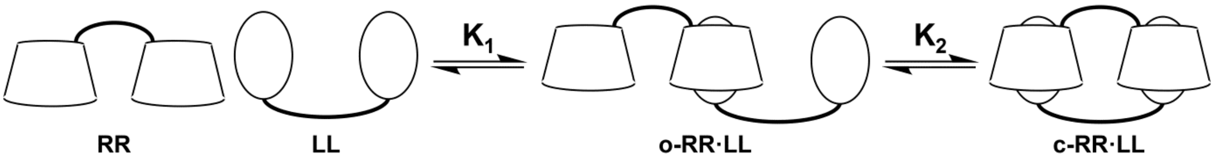

$$K_1 = 4K_i \qquad K_2 = \frac{1}{2}K_i EM$$

$$[RR]_0 = [RR] + [o - RR \cdot LL] + [c - RR \cdot LL] = [RR] + K_1[RR][LL] + K_1K_2[RR][LL]$$

$$[LL]_0 = [LL] + [o - RR \cdot LL] + [c - RR \cdot LL] = [LL] + K_1[RR][LL] + K_1K_2[RR][LL]$$

1:1 overall complexation model of a divalent receptor and a divalent ligand

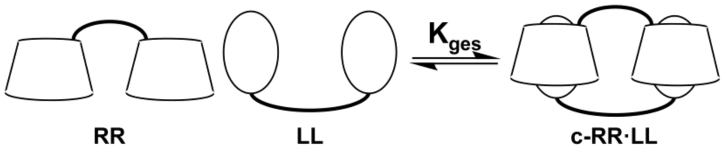

$$K_{overall} = \frac{[c - RR \cdot LL]}{[RR][LL]}$$

$$[RR]_0 = [RR] + [c - RR \cdot LL]$$

$$= [RR] + K_{overall}[RR][LL]$$

$$[LL]_0 = [LL] + [c - RR \cdot LL]$$

$$= [LL] + K_{overall}[RR][LL]$$

2:1 interaction of a divalent receptor and a divalent ligand

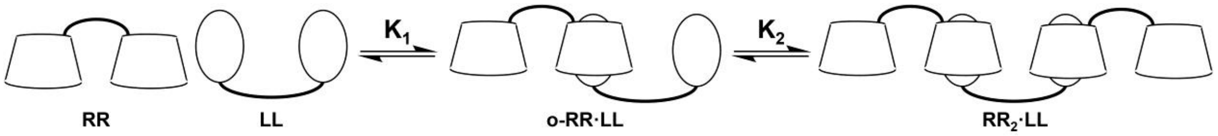

$$K_1 = 4K_i \qquad K_2 = K_i$$

$$[RR]_0 = [RR] + [o - RR \cdot LL] + 2[RR_2 \cdot LL] = [RR] + K_1[RR][LL] + 2K_1K_2[RR]^2[LL]$$

$$[LL]_0 = [LL] + [o - RR \cdot LL] + [RR_2 \cdot LL] = [LL] + K_1[RR][LL] + K_1K_2[RR]^2[LL]$$

2:1 interaction of a trivalent receptor and a trivalent ligand

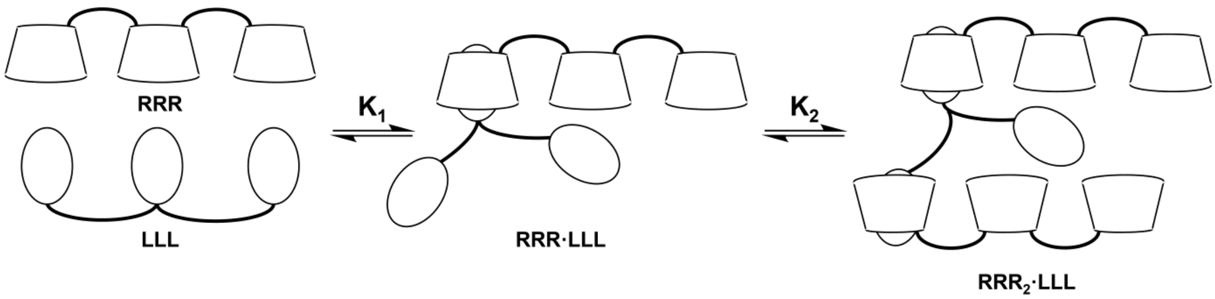

$$K_1 = 9K_i \qquad K_2 = 3K_i$$

$$[RRR]_0 = [RRR] + [RRR \cdot LLL] + 2[RRR_2 \cdot LLL]$$
$$= [RRR] + K_1[RRR][LLL] + 2K_1K_2[RRR]^2[LLL]$$

$$[LLL]_0 = [LLL] + [RRR \cdot LLL] + [RRR_2 \cdot LLL]$$
$$= [LLL] + K_1[RRR][LLL] + K_1K_2[RRR]^2[LLL]$$

3:1 interaction of a trivalent receptor and a trivalent ligand

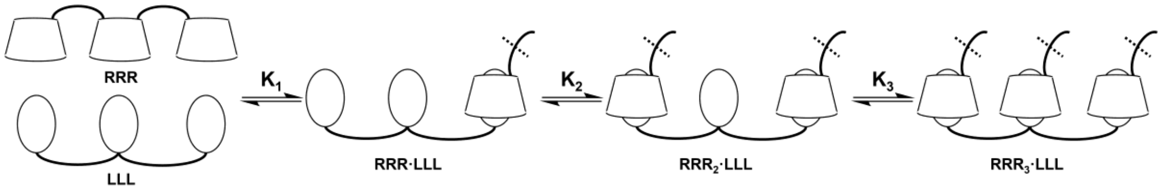

$$K_1 = 9K_i \qquad K_2 = 3K_i \qquad K_3 = K_i$$

$$[RRR]_0 = [RRR] + [RRR \cdot LLL] + 2[RRR_2 \cdot LLL] + 3[RRR_3 \cdot LLL]$$
$$= [RRR] + K_1[RRR][LLL] + 2K_1K_2[RRR]^2[LLL] + 3K_1K_2K_3[RRR]^3[LLL]$$

$$[LLL]_0 = [LLL] + [RRR \cdot LLL] + [RRR_2 \cdot LLL] + [RRR_3 \cdot LLL]$$
$$= [LLL] + K_1[RRR][LLL] + K_1K_2[RRR]^2[LLL] + K_1K_2K_3[RRR]^3[LLL]$$

1:1 overall complexation model of a trivalent receptor and a trivalent ligand

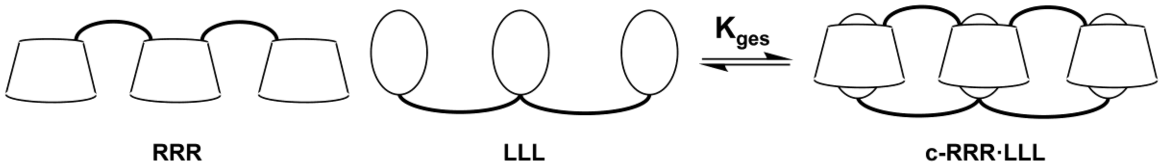

$$K_{ges} = \frac{[c - RRR \cdot LLL]}{[RRR][LLL]}$$

$$[RRR]_0 = [RRR] + [c - RRR \cdot LLL]$$

$$= [RRR] + K_{ges}[RRR][LLL]$$

$$[\boldsymbol{LLL}]_{\mathbf{0}} = [\boldsymbol{LLL}] + [\boldsymbol{c} - \boldsymbol{RRR} \cdot \boldsymbol{LLL}]$$

$$= [\boldsymbol{LLL}] + \boldsymbol{K_{ges}}[\boldsymbol{RRR}][\boldsymbol{LLL}]$$

ITC measurements

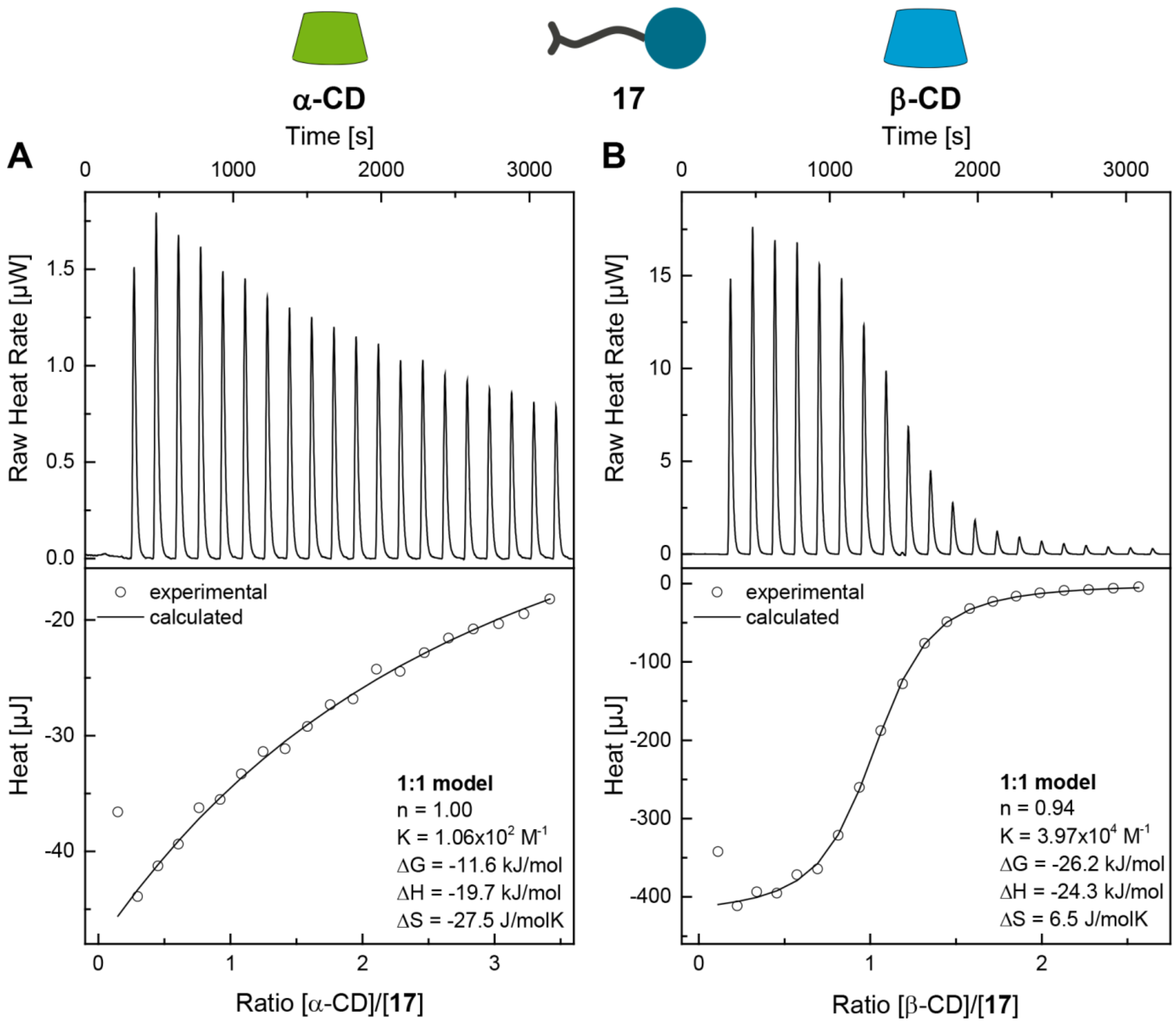

Figure 1: ITC measurements of the interactions of **17** with α-CD (A) and β-CD (B): α-CD (c = 10 mM) resp.. β-CD (c = 7.0 mM) added to **17** (c = 1.0 mM), measured in 100 mM phosphate buffer pH 7.4 at 25 °C. Top: Experimental raw heat rate of host-guest complexation. Down: Integrals of the peaks (o), fit for determination of thermodynamic parameters (–) and resulting values with the given model.

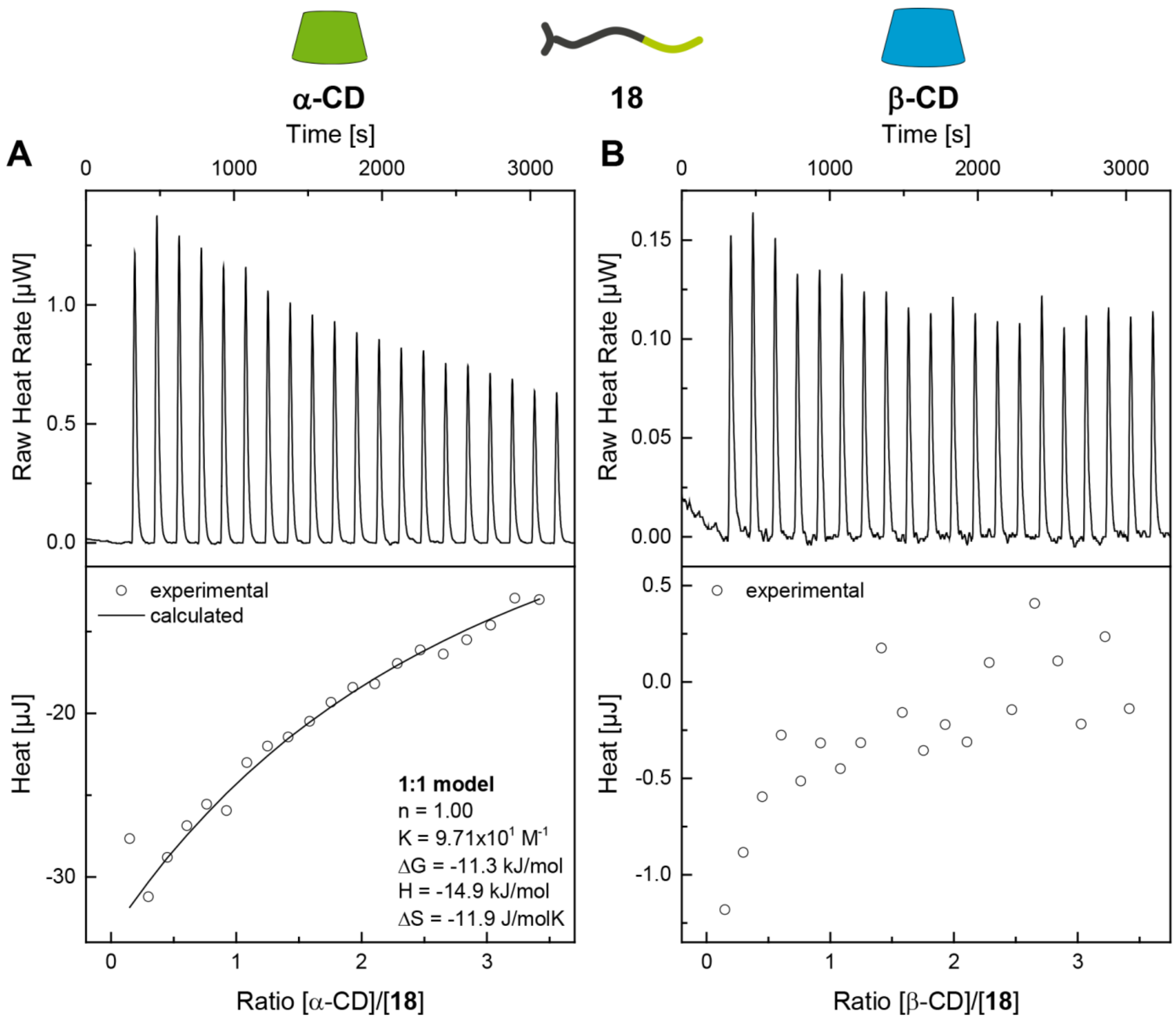

Figure 2: ITC measurements of the interactions of **18** with α-CD (A) and β-CD (B): α-CD (c = 10 mM) resp.. β-CD (c = 10.0 mM) added to **18** (c = 1.0 mM), measured in 100 mM phosphate buffer pH 7.4 at 25 °C. Top: Experimental raw heat rate of host-guest complexation. Down: Integrals of the peaks (o), fit for determination of thermodynamic parameters (–) and resulting values with the given model.

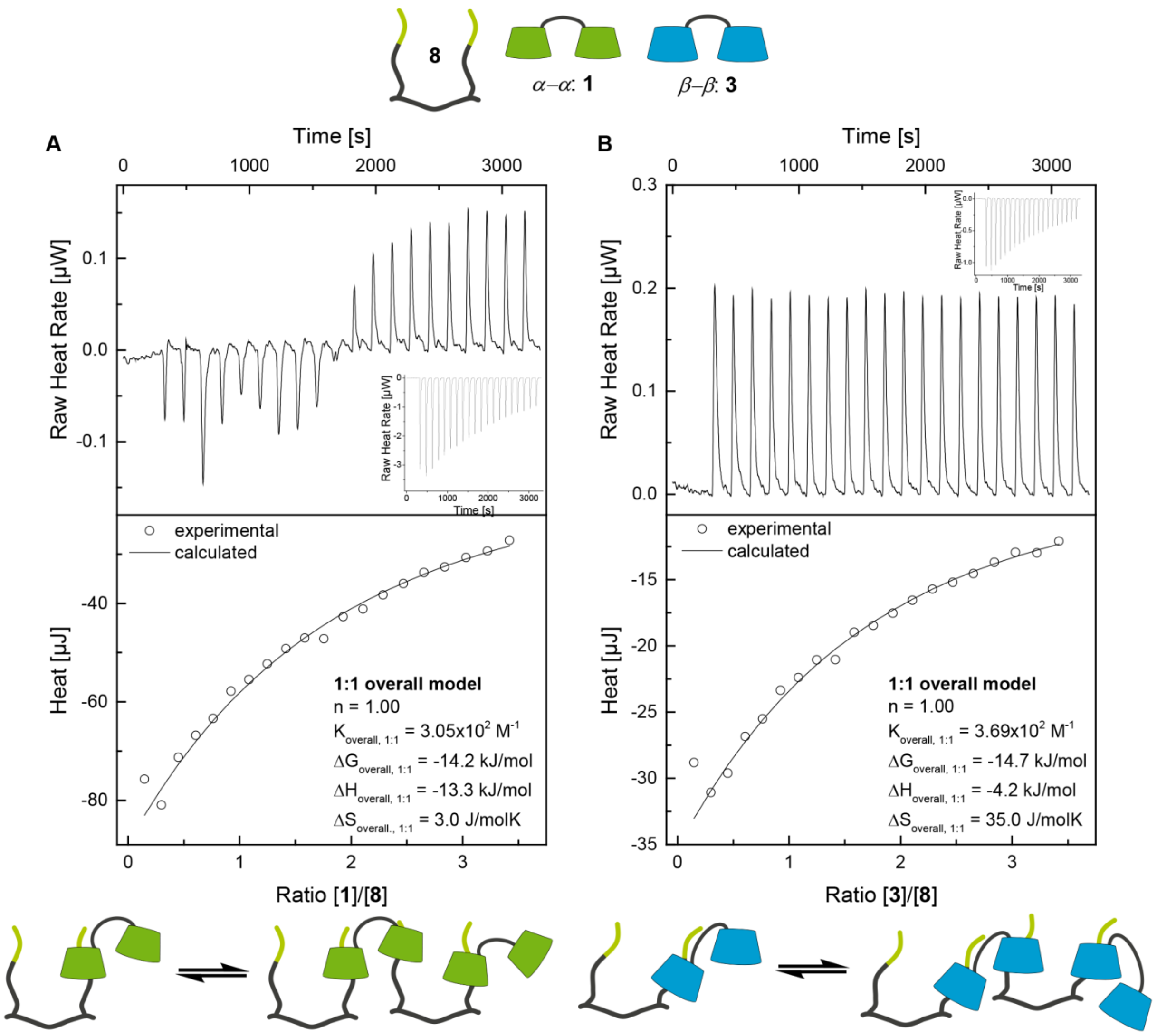

Figure 3: ITC measurements of the interactions of **8** with **1** (A) and **3** (B): **1** (c = 10 mM) resp.. **3** (c = 7.0 mM) added to **8** (c = 1.0 mM), measured in 100 mM phosphate buffer pH 7.4 at 25 °C. Top: Experimental raw heat rate of host-guest complexation. Small graphs show the dilution measurements of the CD dimers. Down: Integrals of the peaks (o), fit for determination of thermodynamic parameters (–) and resulting values with the given model. Additionally, the structures of the host-guest systems are schematically drawn.

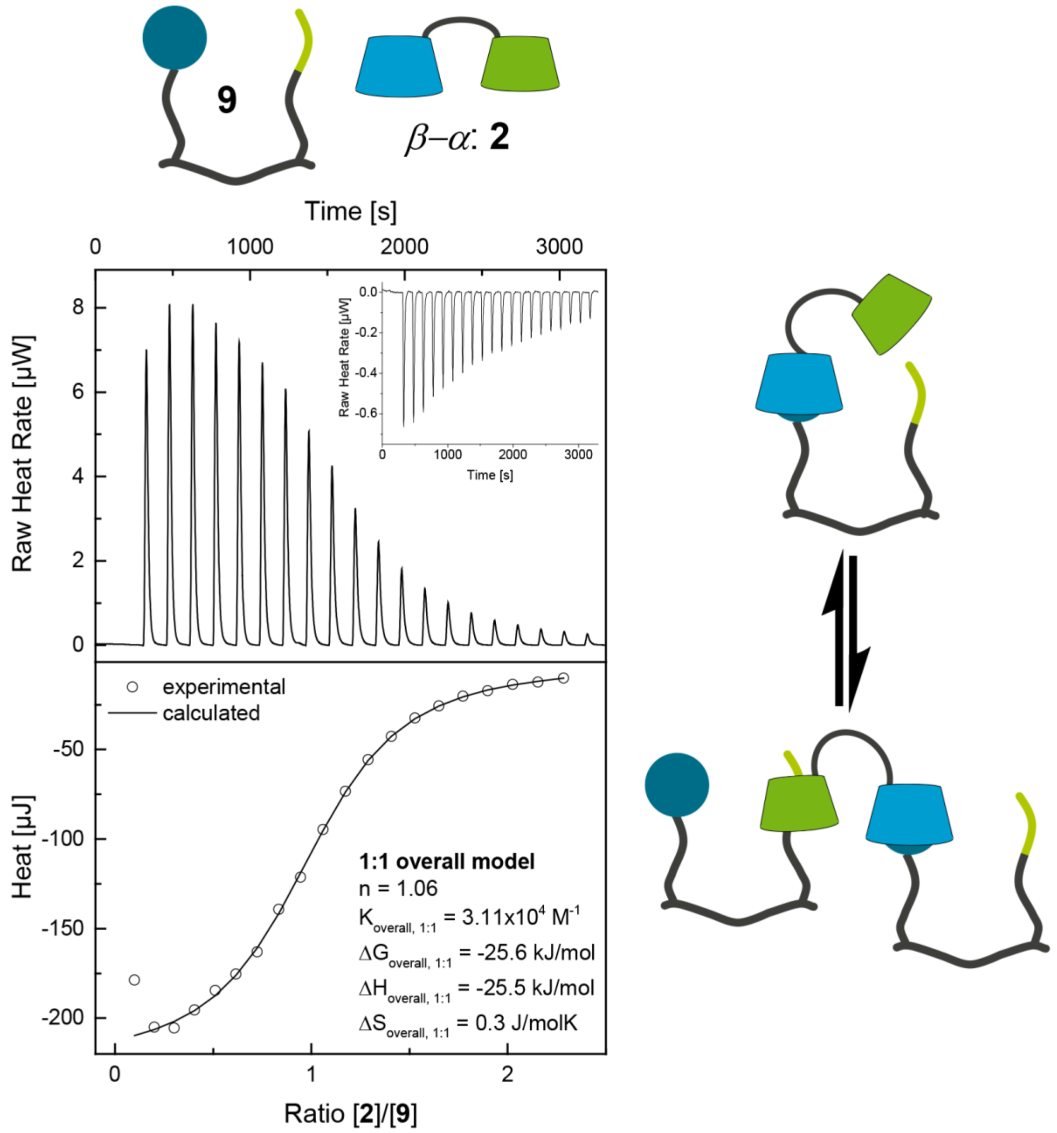

Figure 4: ITC measurements of the interaction of **9** with **2**: **2** (c = 3.5 mM) added to **9** (c = 0.5 mM), measured in 100 mM phosphate buffer pH 7.4 at 25 °C. Top: Experimental raw heat rate of host-guest complexation. Small graphs show the dilution measurements of the CD dimers. Down: Integrals of the peaks (o), fit for determination of thermodynamic parameters (–) and resulting values with the given model. Additionally, the structures of the host-guest systems are schematically drawn.

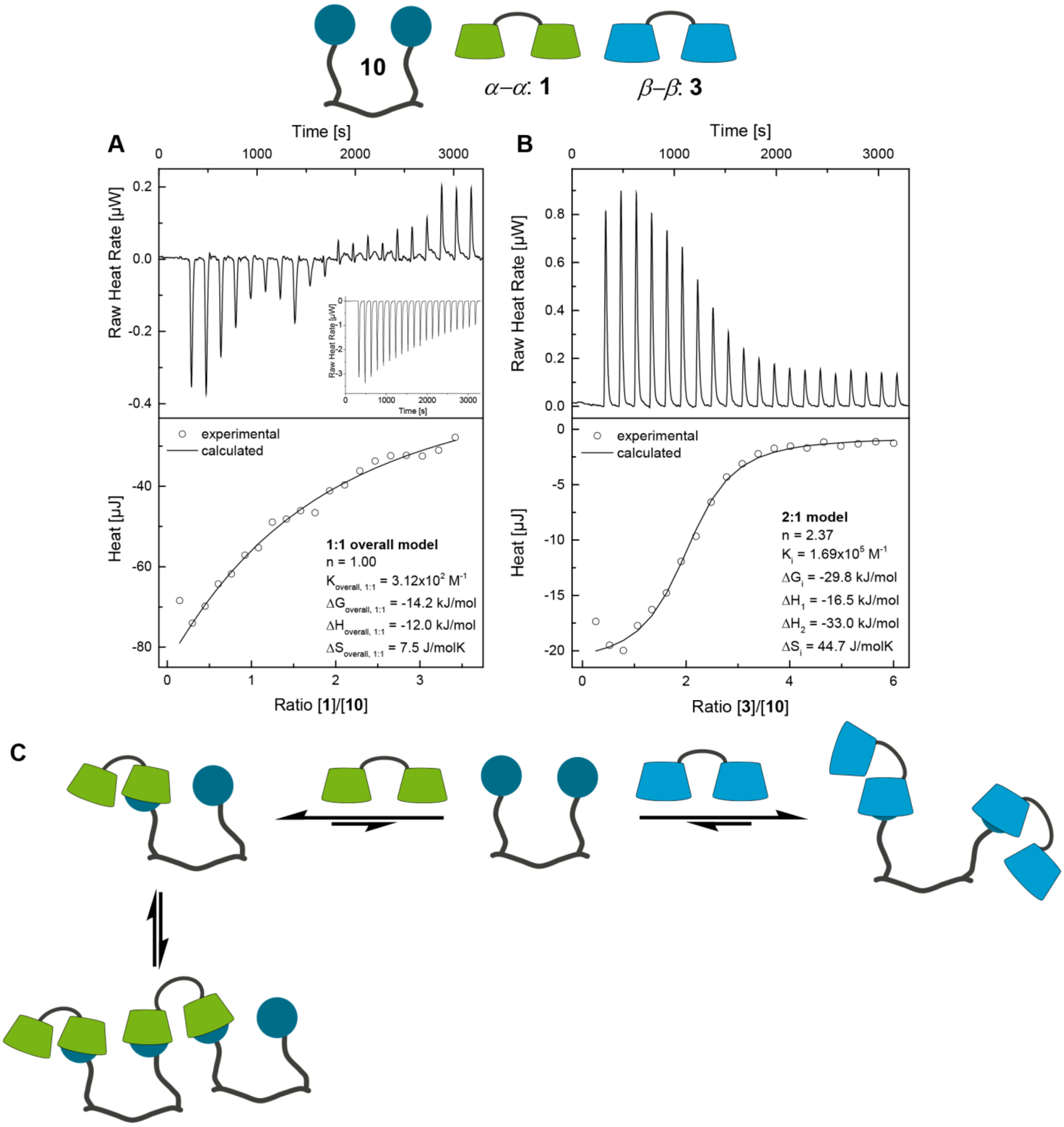

Figure 5: ITC measurements of the interactions of **10** with **1** (A) and **3** (B): **1** (c = 10 mM) added to **10** (c = 1.0 mM) resp.. **3** (c = 0.5 mM) added to **8** (c = 0.25 µM), measured in 100 mM phosphate buffer pH 7.4 at 25 °C. Top: Experimental raw heat rate of host-guest complexation. Small graphs show the dilution measurements of the CD dimers. Down: Integrals of the peaks (o), fit for determination of thermodynamic parameters (–) and resulting values with the given model. Additionally, the structures of the host-guest systems are schematically drawn (C).

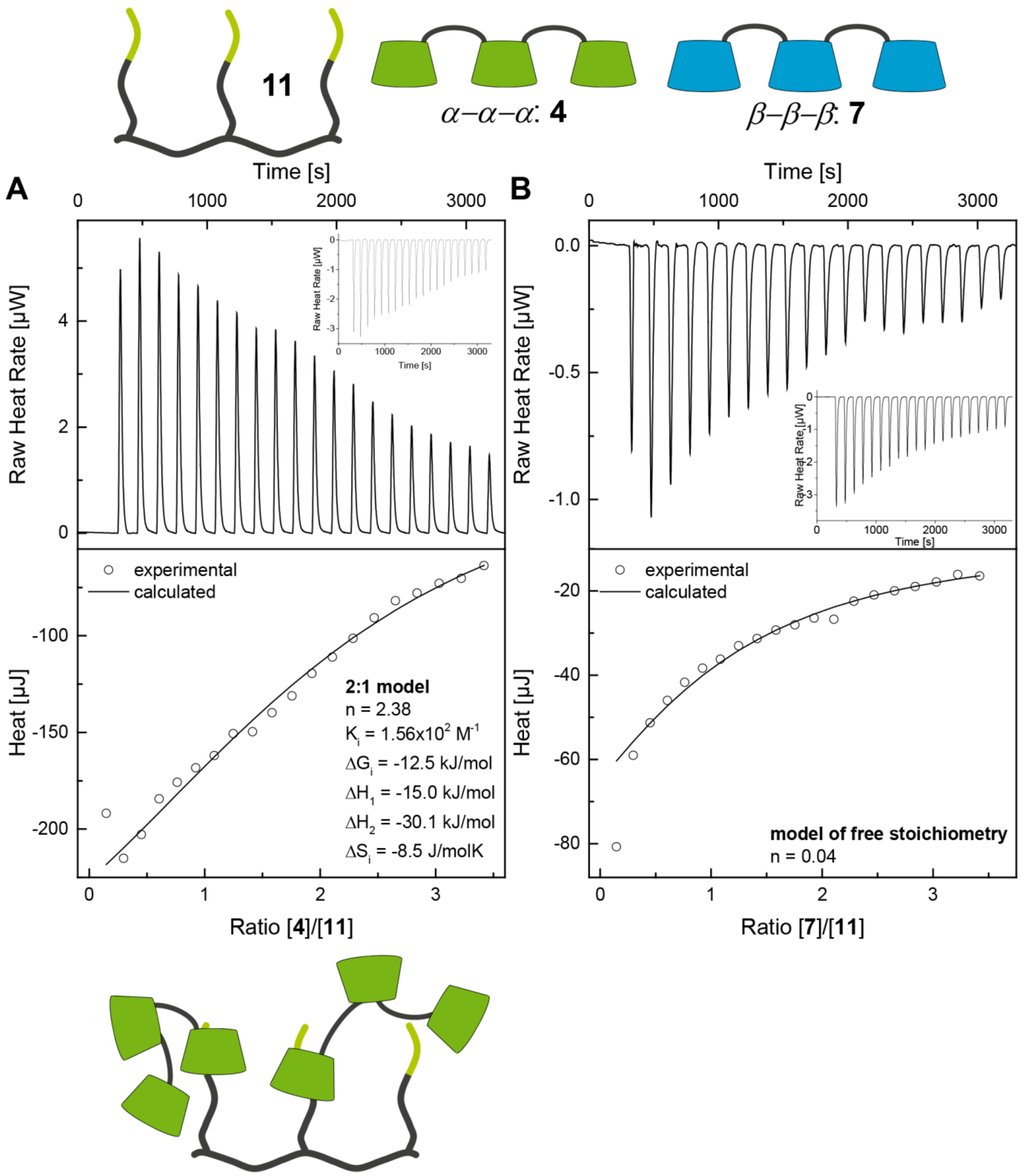

Figure 6: ITC measurements of the interactions of **11** with **4** (A) and **7** (B): **4** (c = 10 mM) resp.. **7** (c = 10 mM) added to **11** (c = 1.0 mM), measured in 100 mM phosphate buffer pH 7.4 at 25 °C. Top: Experimental raw heat rate of host-guest complexation. Small graphs show the dilution measurements of the CD trimers. Down: Integrals of the peaks (o), fit for determination of thermodynamic parameters (–) and resulting values with the given model. Additionally, the structures of the host-guest systems are schematically drawn.

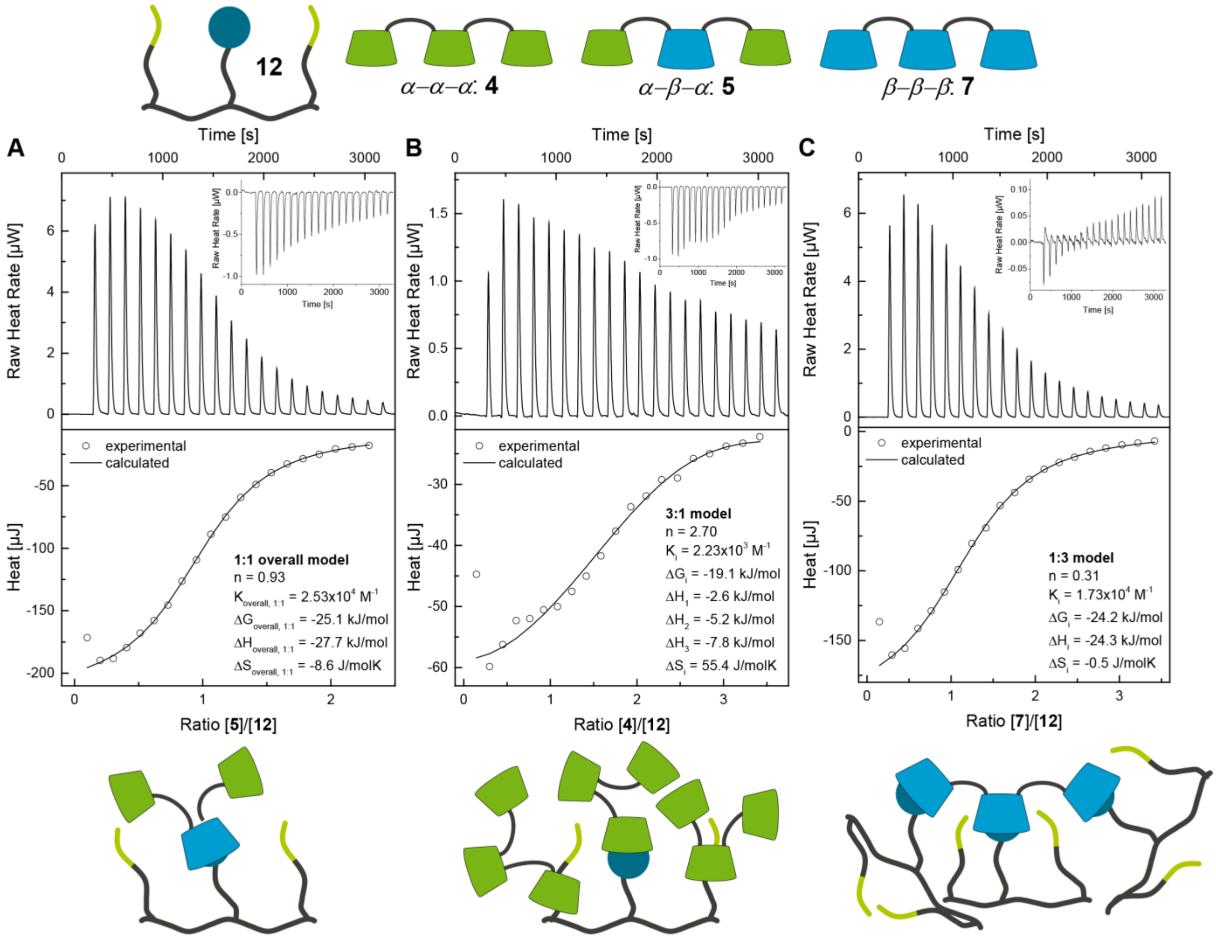

Figure 7: ITC measurements of the interactions of **12** with **6** (A), **4** (B) and **7** (C): **5** (c = 3.0 mM) added to **12** (c = 0.5 mM), **4** (c = 5.0 mM) added to **12** (c = 0.5 mM), **7** (c = 1.0 mM) added to **12** (c = 0.4 mM); measured in 100 mM phosphate buffer pH 7.4 at 25 °C. Top: Experimental raw heat rate of host-guest complexation. Small graphs show the dilution measurements of the CD dimers. Down: Integrals of the peaks (o), fit for determination of thermodynamic parameters (–) and resulting values with the given model. Additionally, the structures of the host-guest systems are schematically drawn.

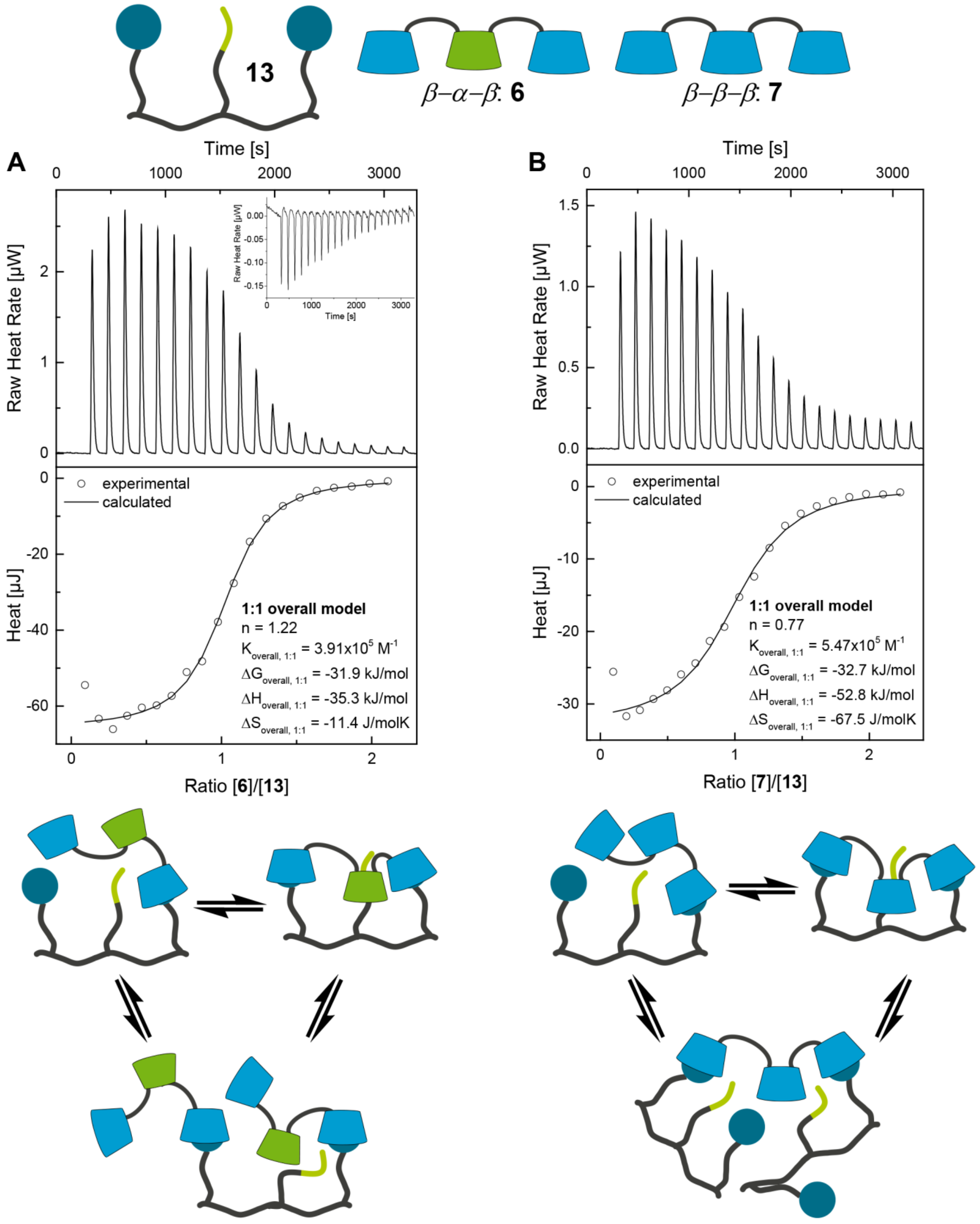

Figure 8: ITC measurements of the interactions of **13** with **6** (A) and **7** (B): **6** (c = 0.75 mM) added to **13** (c = 0.1 mM), **7** (c = 0.25 mM) added to **13** (c = 50 µM); measured in 100 mM phosphate buffer pH 7.4 at 25 °C. Top: Experimental raw heat rate of host-guest complexation. Small graphs show the dilution measurements of the CD dimers. Down: Integrals of the peaks (o), fit for determination of thermodynamic parameters (–) and resulting values with the given model. Additionally, the structures of the host-guest systems are schematically drawn.

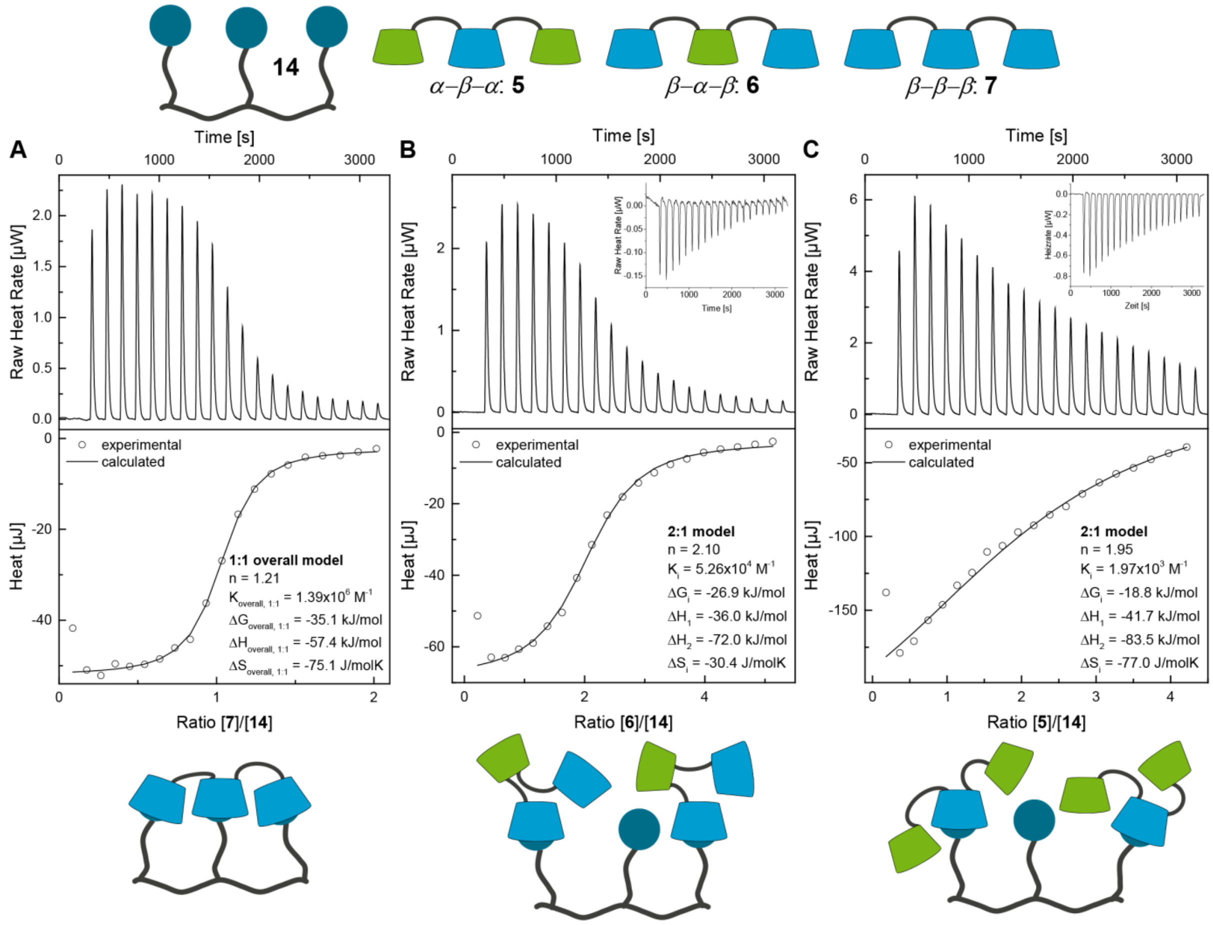

Figure 9: ITC measurements of the interactions of **14** with **7** (A), **6** (B) and **5** (C): **7** (c = 0.35 mM) added to **14** (c = 50 µM), **6** (c = 0.75 mM) added to **14** (c = 50 µM), **5** (c = 3.0 mM) added to **14** (c = 0.25 mM); measured in 100 mM phosphate buffer pH 7.4 at 25 °C. Top: Experimental raw heat rate of host-guest complexation. Small graphs show the dilution measurements of the CD dimers. Down: Integrals of the peaks (o), fit for determination of thermodynamic parameters (–) and resulting values with the given model. Additionally, the structures of the host-guest systems are schematically drawn.
